# Supplementary material for: A comparative analysis of CD70-directed CAR-T cells for glioblastoma treatment demonstrates a superior efficacy of the ligand-based construct
Source: Mol Ther Oncol. 2026 Jan 19;34(1):201134. doi: 10.1016/j.omton.2026.201134 (PMC12915223; doi:10.1016/j.omton.2026.201134)
Supplement: Document S1. Figures S1–S9, Table S1, and supplemental methods [file mmc1.pdf]

OMTON, Volume *34*

Supplemental information

A comparative analysis of CD70-directed CAR-T cells for glioblastoma treatment demonstrates a superior efficacy of the ligand-based construct

Alexandros Kourtesakis, Hiu Nam Hannah Chow, Eileen Bailey, Sandra Horschitz, Ammar Jabali, Rainer Will, Christoph Schifflers, Abigail K. Suwala, Hannah Rohdjess, Melissa Hahn, Yu-Chan Chih, Ling Hai, Denise Reibold, Sonja Pusch, Manuel Fischer, Ralph Sinn, Dennis Alexander Agardy, Dirk Carsten Frieder Hoffmann, Michael O. Breckwoldt, Robin Wagener, Leon Kaulen, Philipp Koch, Andreas von Deimling, Lukas Bunse, Michael Platten, Felix Sahm, Carsten Müller-Tidow, Michael Schmitt, Wolfgang Wick, Tim Sauer, and Tobias Kessler

Supplemental Figures

A)

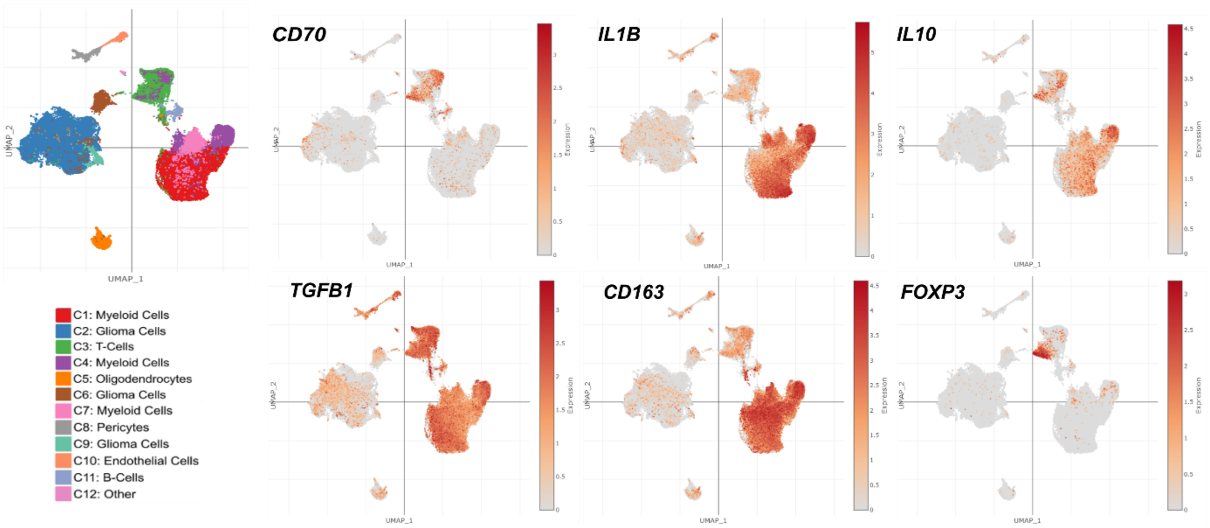

B)

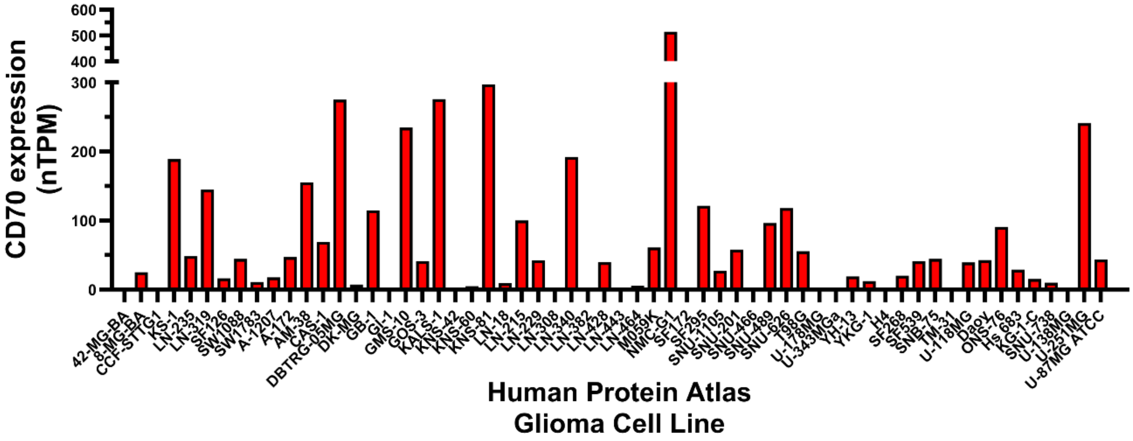

C)

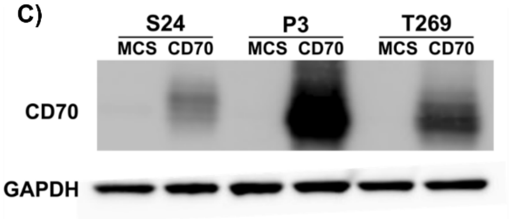

Figure S1: CD70 validation as a therapeutic target in GB. A) Expression of *CD70* and selected markers from a published single cell RNAseq dataset. **B)** *CD70* gene expression levels in selected glioma cell lines from Human Protein Atlas (version 24.0), determined by RNAseq. **C)** Investigation of CD70 expression in generated GB OE models by Western blot.

A)

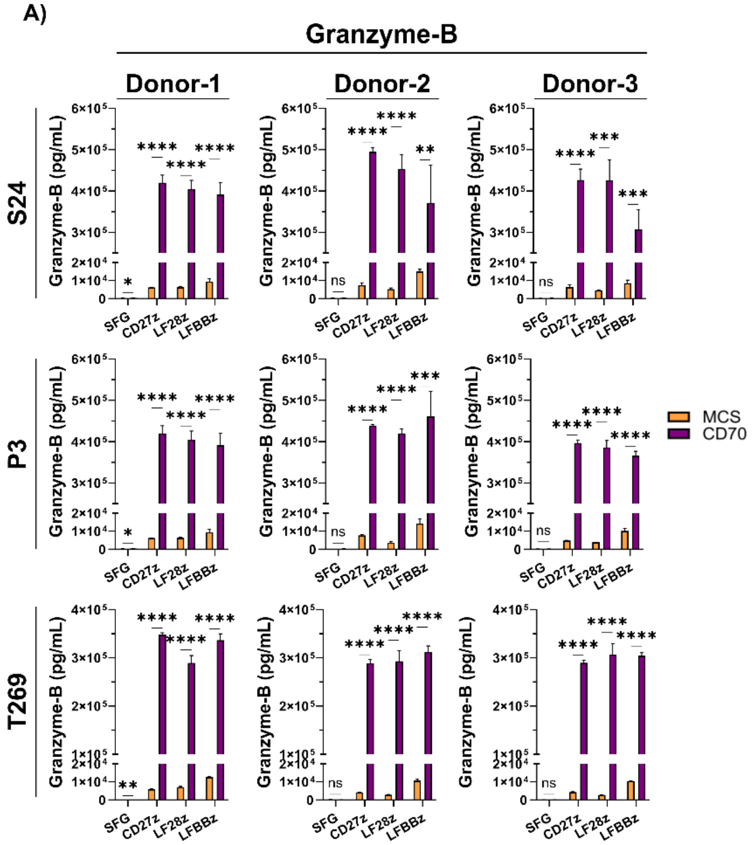

	S24/CD70			P3/CD70			T269/CD70		
	Donor-1	Donor-2	Donor-3	Donor-1	Donor-2	Donor-3	Donor-1	Donor-2	Donor-3
CD27z vs LF28z	ns	ns	ns	ns	ns	ns	***	ns	ns
CD27z vs LFBBz	*	*	*	ns	ns	*	ns	ns	ns
LF28z vs LFBBz	*	ns	*	ns	ns	ns	**	ns	ns

B)

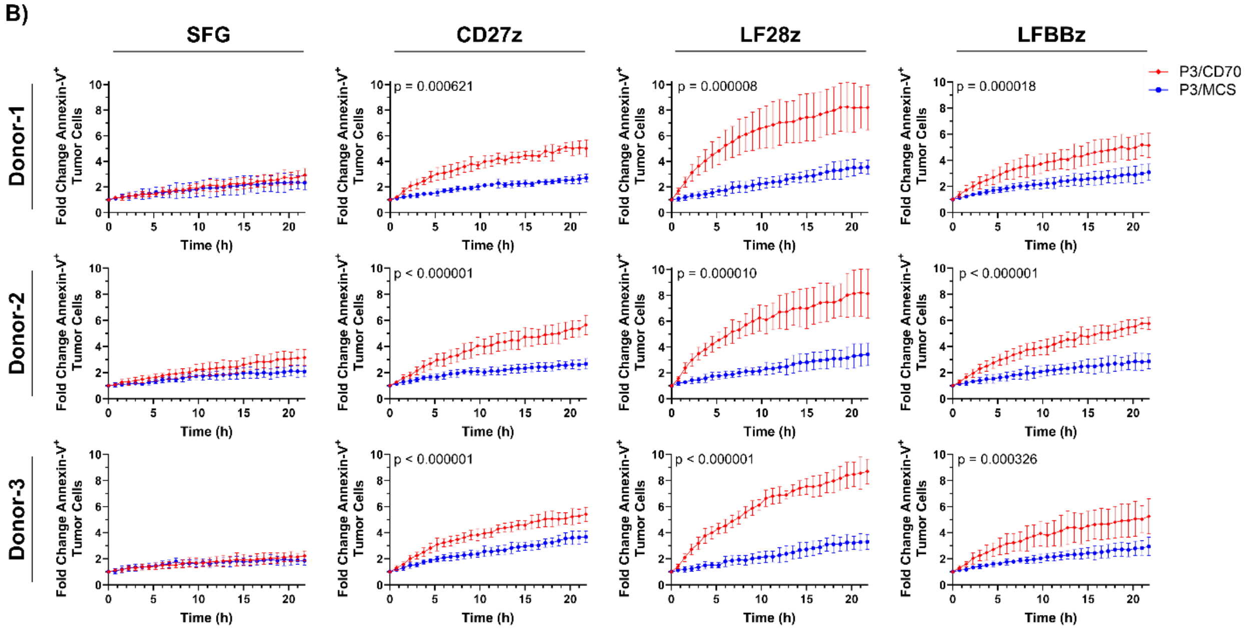

Figure S2: Assessment of Granzyme-B and apoptosis in co-culture between GB cells and CD70-directed CAR-T cells. A) Measurement of secreted Granzyme-B in the SN of GB/CAR-T cell co-cultures by ELISA. N = 3 biological replicates per group. For comparison between MCS and CD70 (upper barplots), an unpaired two-tailed t-test was used. For comparisons among constructs (bottom panel), a one-way ANOVA followed by a post-hoc Holm-Šídák multiple comparisons test was used. **B)** Quantification of Annexin-V binding by tumor cells during co-culture with CD70-directed CAR-T cells on the Incucyte platform. N = 2 biological replicates per group. Every biological replicate is the mean of N = 5 technical replicates. Timepoint intervals = 45 min. A two-tailed student's t-test was performed using the values of the last measured timepoint to determine statistical significance. For A) and B), data presented as mean (SD). *p <0.05, **p<0.01, ***p<0.001, ****p<0.0001; n.s., not significant.

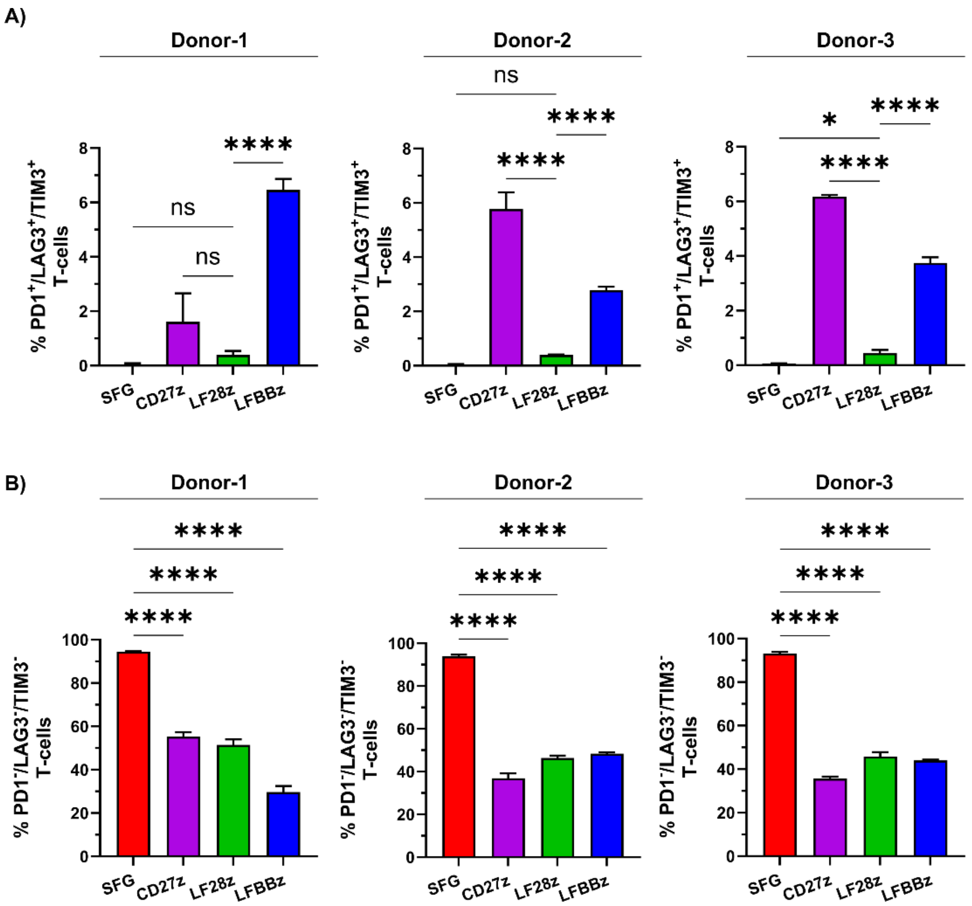

<u>Figure S3</u>: Assessment of exhaustion markers on the CAR-T cell surface after engagement with CD70-positive GB cells. A) Quantification of PD1/LAG3/TIM3 triple-positive effector cells after co-culture with P3/CD70 cells, determined by flow cytometry. **B)** Quantification of PD1/LAG3/TIM3 triple-negative effector cells after co-culture with P3/CD70 cells, determined by flow cytometry. For A) and B), isotype controls were used for gating. N=3 biological replicates per group. Data gated on single live $CD3^+$/$tdTomato^+$ cells. A one-way ANOVA followed by a Dunnett's multiple comparisons test was performed to assess significance. Data presented as mean (SD). *p <0.05, **p<0.01, ***p<0.001, ****p<0.0001; n.s., not significant.

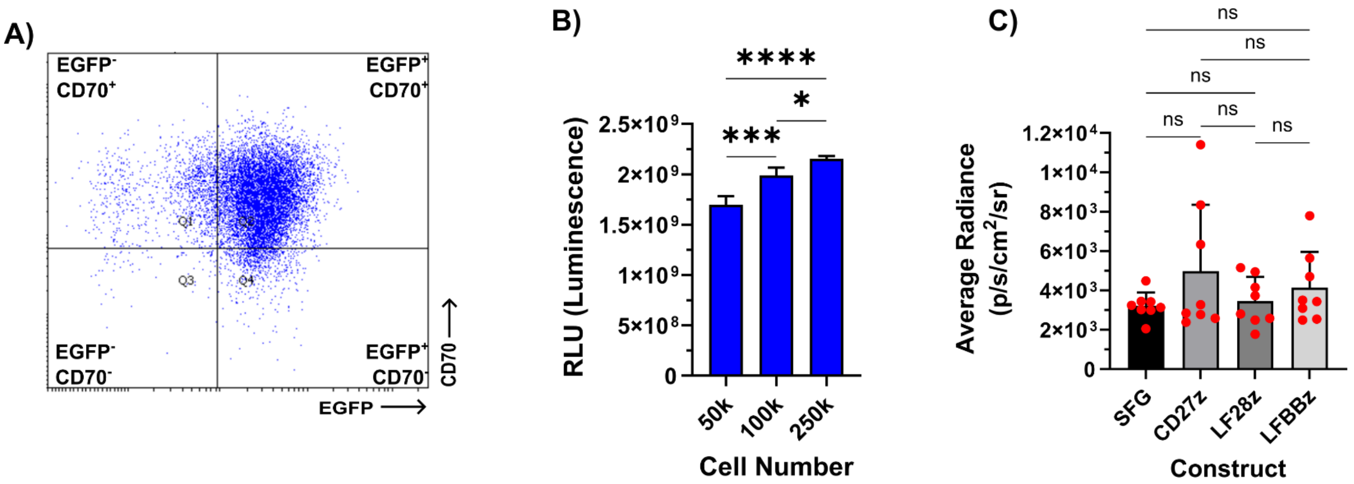

<u>Figure S4</u>: Verification of the NLuc/EGFP gene functionality before orthotopic implantation and animal stratification into treatment groups. A) Evaluation of the EGFP and CD70 signal of P3/CD70 GB cells transduced with the pCDH_NLuc/EGFP lentiviral construct by flow cytometry. An isotype and unstained control was used for gating. Data gated on single live cells. **B)** *In vitro* luminescence assay using GB cells from A). N = 3 biological replicates per group. A one-way ANOVA with a post-hoc Tukey's multiple comparisons test was performed for statistical significance. **C)** Comparison of tumor cell signal in NSG mice orthotopically implanted with GB cells one day before CAR-T cell treatment, stratified in treatment groups, determined by BLI. Each dot represents an animal. N = 8 animals per group. A Welch's ANOVA with a post-hoc Dunnett's T3 multiple comparisons test was perform to assess statistical significance. For B) and C), data presented as mean (SD). *p <0.05, **p<0.01, ***p<0.001, ****p<0.0001; n.s., not significant.

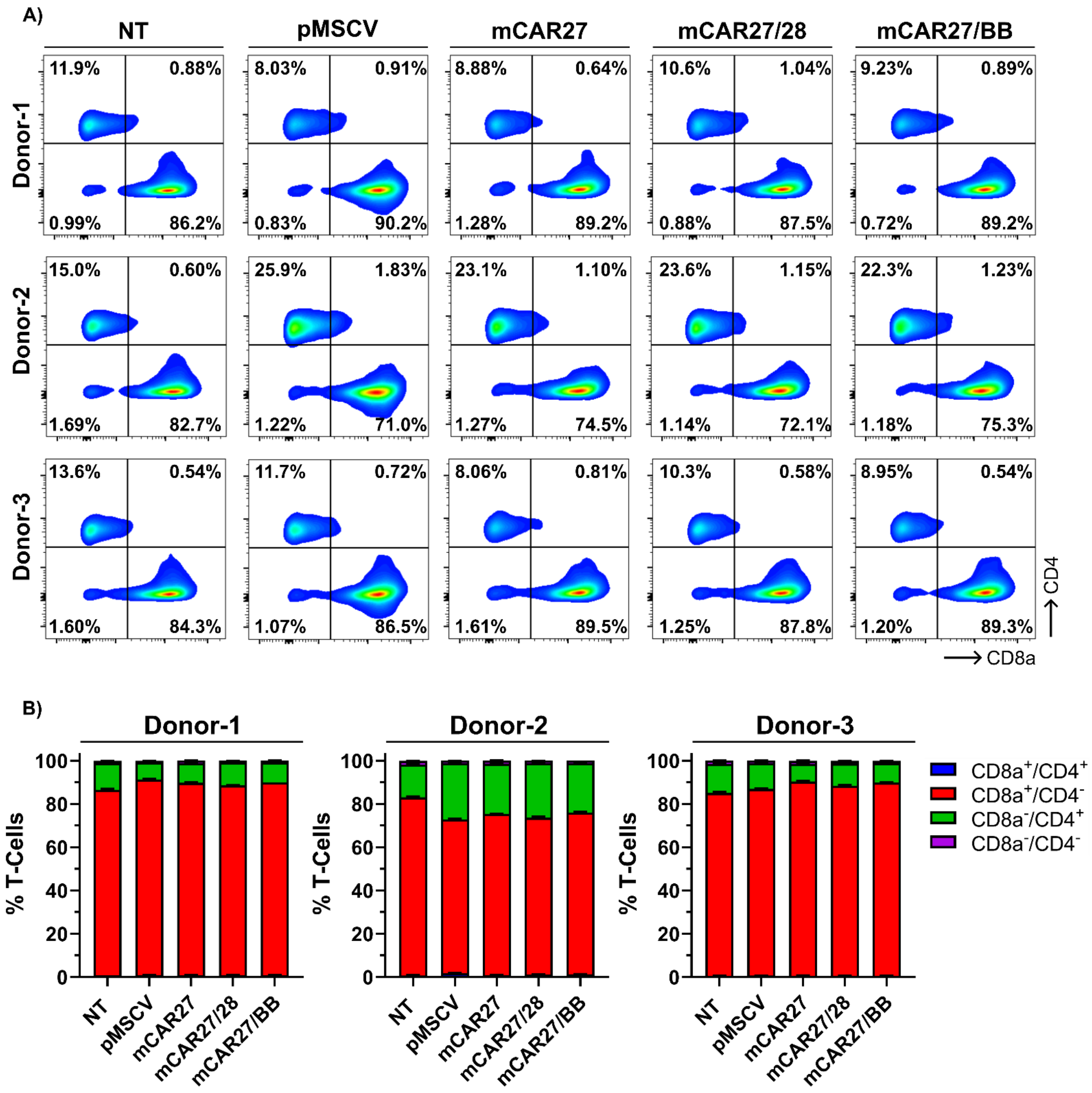

	Donor-1					Donor-2					Donor-3				
	NT	pMSCV	mCAR27	mCAR27/28	mCAR27/BB	NT	pMSCV	mCAR27	mCAR27/28	mCAR27/BB	NT	pMSCV	mCAR27	mCAR27/28	mCAR27/BB
CD8a$^+$/CD4$^-$ vs CD8a$^+$/CD4$^+$	****	****	****	****	****	****	****	****	****	****	****	****	****	****	****
CD8a$^+$/CD4$^-$ vs CD8a$^-$/CD4$^+$	****	****	****	****	****	****	****	****	****	****	****	****	****	****	****
CD8a$^+$/CD4$^-$ vs CD8a$^-$/CD4$^-$	****	****	****	****	****	****	****	****	****	****	****	****	****	****	****

<u>Figure S5</u>: Phenotyping of freshly produced mCD27-based mCD70-targeting CAR-T cells. A) Expression of mCD8a and mCD4 on transduced murine T-cells, determined by flow cytometry. Indicative results from one out of N=3 biological replicates per mouse donor per group. Isotype controls were used for gating. Data gated on single live $CD3^{+}$ cells (NT) or single live $CD3^{+}$/$tdTomato^{+}$ cells (pMSCV, mCAR27, mCAR27/28, mCAR27/BB). **B)** Quantification and comparison of different fractions from A). A one-way ANOVA followed by a Dunnett's multiple comparisons test was performed to assess significance. Data presented as mean (SD). *p <0.05, **p<0.01, ***p<0.001, ****p<0.0001; n.s., not significant.

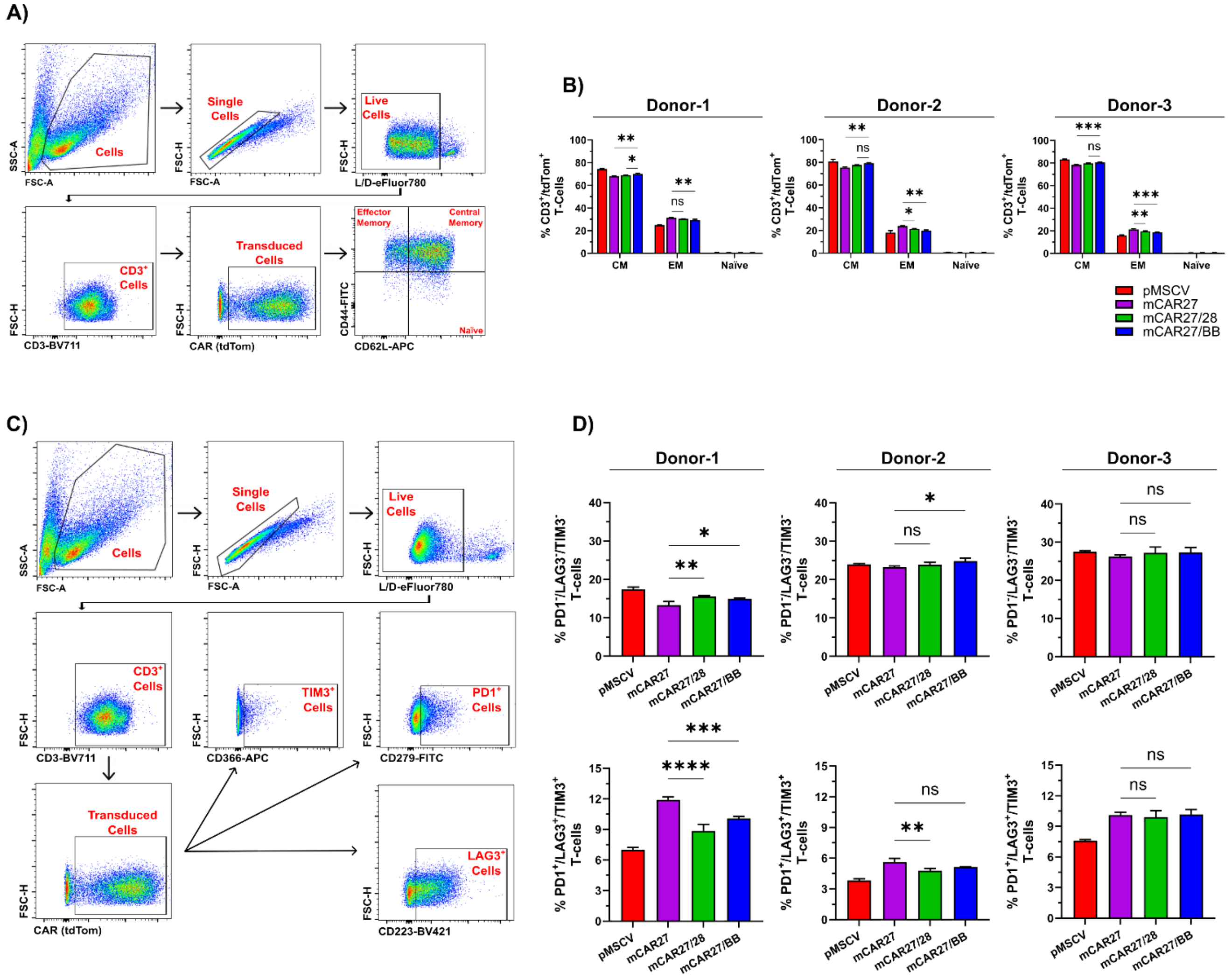

Figure S6: Assessment of memory markers and inhibitory receptors on mCAR-T cells. A) Gating strategy for immune cell memory marker quantification. **B)** Assessment of immune cell memory markers on the surface of mCAR-T cells by flow cytometry. **C)** Gating strategy for exhaustion marker quantification. **D)** Assessment of exhaustion markers on the surface of mCAR-T cells by flow cytometry. For B) and D), N=3 biological replicates per group. Data gated on single live CD3^{+}/tdTomato^{+} cells. Isotype control antibodies were used for gating. A one-way ANOVA with a post-hoc Holm-Šídák test was used for significance. Data presented as mean (SD). *p <0.05, **p<0.01, ***p<0.001, ****p<0.0001; n.s., not significant.

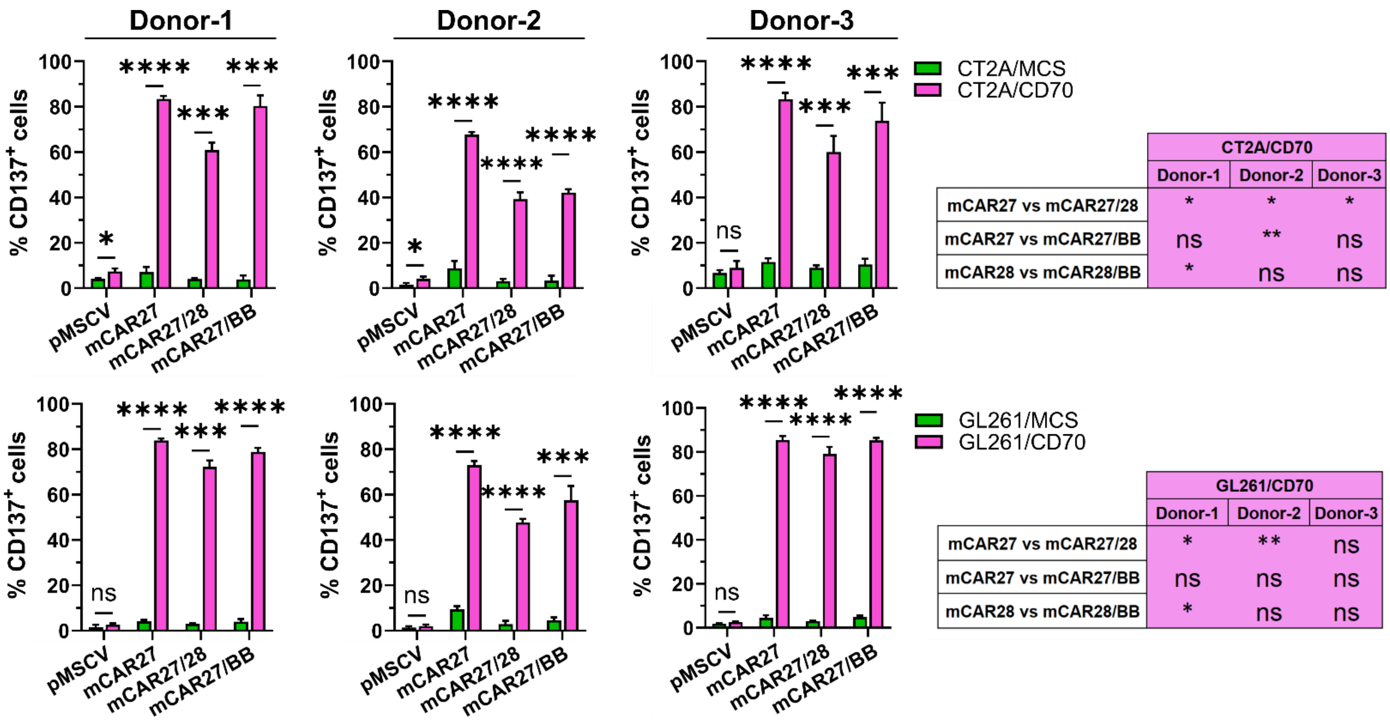

<u>Figure S7</u>: Assessment of mCD70-targeting CAR-T cell activation upon co-culture with target murine GB cells. A) Measurement of activation marker mCD137 on the surface of murine CAR-T cells after O/N co-culture with the generated murine GB models by flow cytometry. N=3 biological replicates per group. Data gated on single live $CD3^+$ cells (NT) or single live $CD3^+$/$tdTomato^+$ cells (pMSCV, mCAR27, mCAR27/28, mCAR27/BB). Isotype control antibodies were used. An unpaired two-tailed t-test was used for comparisons. A one-way ANOVA with a post-hoc Holm-Šídák test was used for significance between selected construct pairs (upper right and bottom right panels). Data presented as mean (SD). *p <0.05, **p<0.01, ***p<0.001, ****p<0.0001; n.s., not significant.

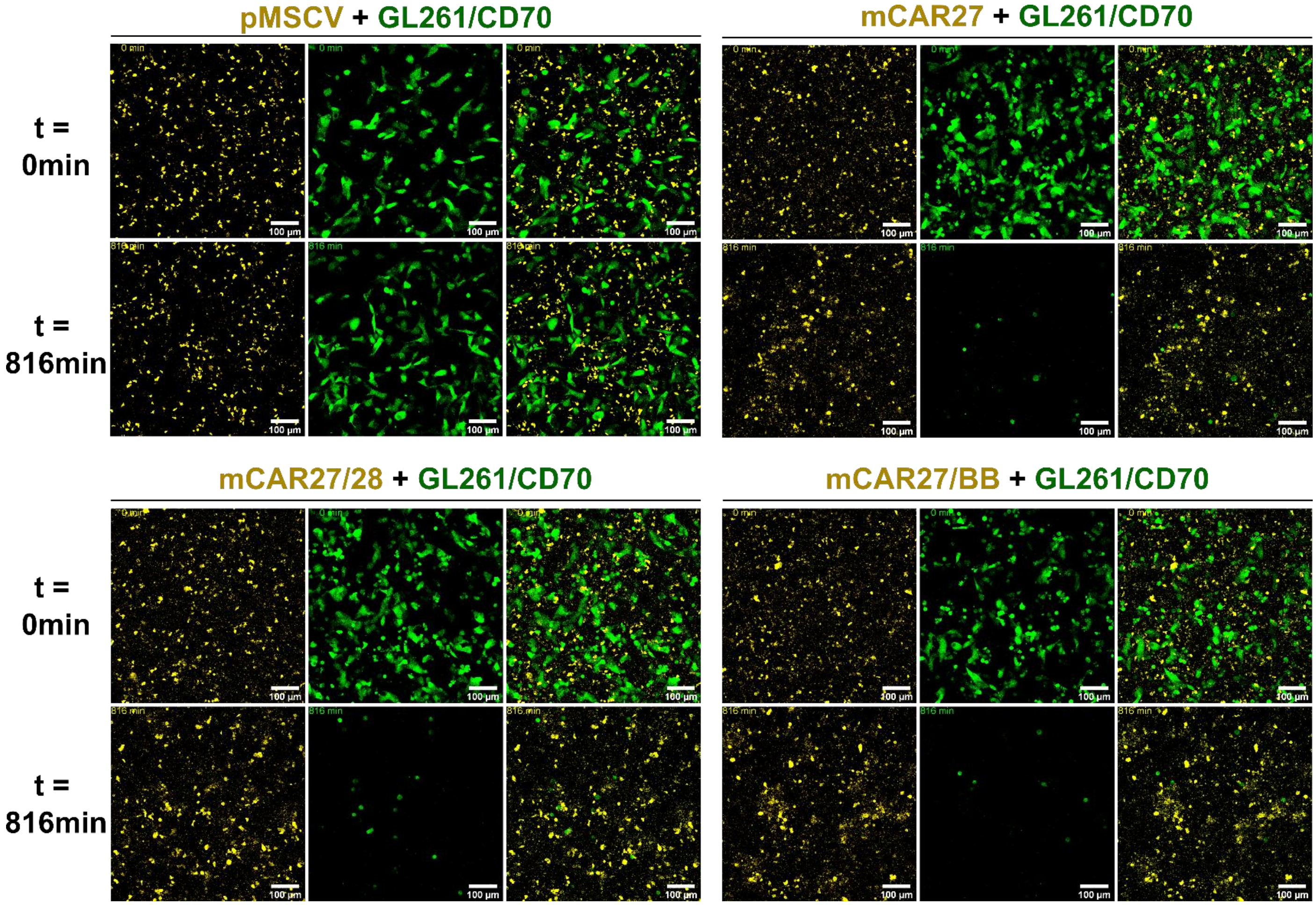

Figure S8: ***In vitro*** **potency of anti-mCD70 ligand-based CAR-T cells.** Confocal IF images of GL261/CD70_EGFP cells at the start (t=0 min) and the end (t=816 min) of co-culture with mCD27-based mCD70-targeting CAR-T cells or mock-transduced murine T-cells. Scale bar = 100 µm.

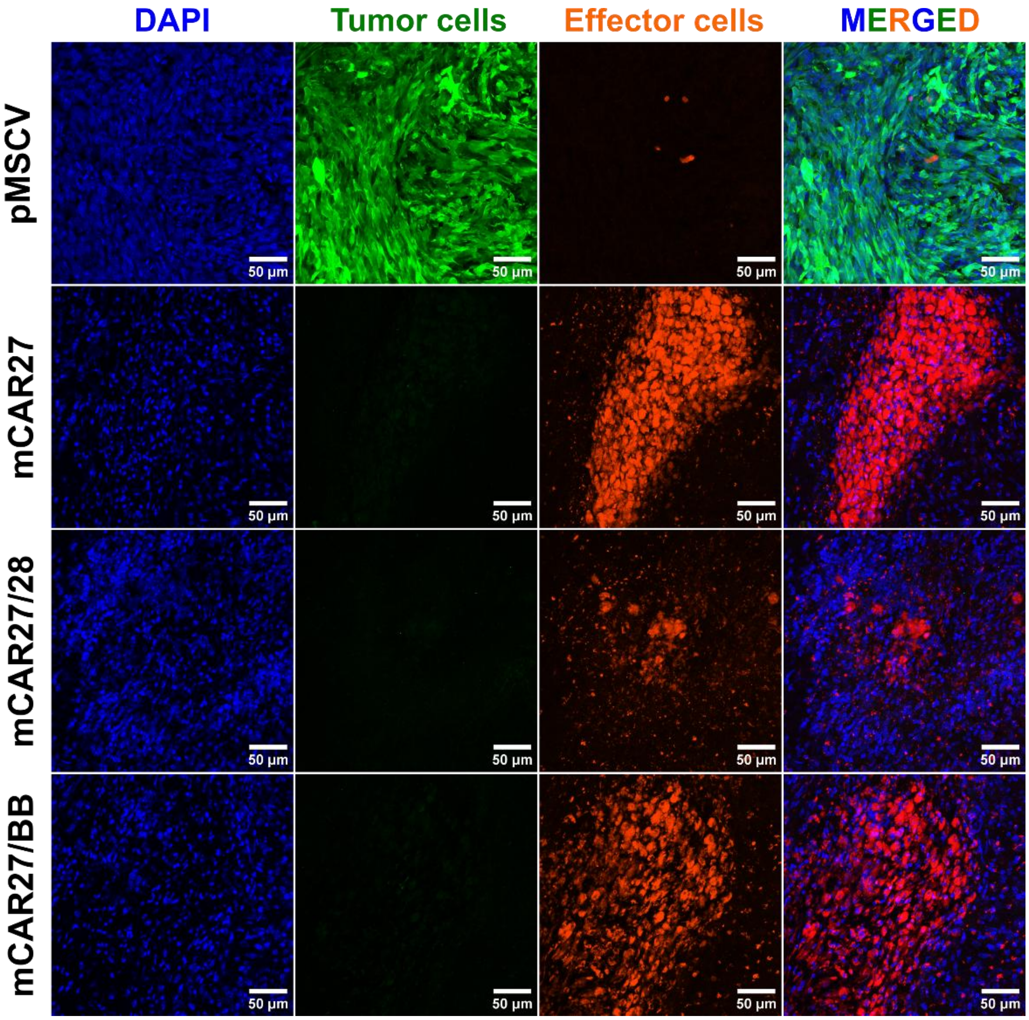

Figure S9: Detection of mCAR-T cells in the brains of treated C57BL/6J mice. Analysis of mCAR-T cell persistence in the brains of C57BL/6J mice treated with mCD70-targeting CAR-T cells from Figure 8A by IF. Representative images from N = 2 mice per treatment arm. Scale bar = 50 μm.

Supplemental Tables

Table S1: Glioblastoma patient characteristics

Sample ID	Age at diagnosis	Gender	Entity	MGMT promoter status	Prior therapy
D170-107	63	male	glioblastoma	methylated	none
D170-108	83	female	glioblastoma	methylated	none
D170-109	83	male	glioblastoma	non methylated	none
D170-112	78	female	glioblastoma	non methylated	none
D170-117	62	female	glioblastoma	non methylated	none
D170-21	63	male	glioblastoma	non methylated	none
D170-27	69	male	glioblastoma	methylated	none
D170-44	64	male	glioblastoma	methylated	none
D170-55	75	female	glioblastoma	non methylated	none
D170-56	80	male	glioblastoma	non methylated	none
D170-63	67	male	glioblastoma	non methylated	none
D170-69	79	female	glioblastoma	methylated	none
D170-83	78	male	glioblastoma	non methylated	none
D170-84	55	female	glioblastoma	non methylated	none
D170-93	42	male	glioblastoma	non methylated	none
D170-99	56	male	glioblastoma	non methylated	none

Supplemental Methods

Western Blot

Total protein from $2*10^6$ GB cells was extracted and quantified with the Pierce™ Bradford Protein Assay Kit (#23200, Thermo Fisher Scientific) according to the manufacturer's recommendations. Samples were prepared in NuPAGE™ LDS Sample Buffer (#NP0007, Invitrogen) and reduced in NuPAGE™ Sample Reducing Agent (#NP0009, Invitrogen). 20 µg of total protein extracted from GB cells was loaded on a 4-12 % (w/v) NuPAGE™ Bis-Tris Mini Protein Gel (#NP0321BOX, Invitrogen) and run at 200 V on the 1 h on the XCell SureLock Mini-Cell system (#EI0001, Invitrogen) in NuPAGE™ MOPS SDS Running Buffer (#NP0001, Invitrogen), in the presence of NuPAGE™ Antioxidant (#NP0005, Invitrogen). The transfer 0.45 µm Immobilon®-P Membrane (#IPVH85R, Merck Millipore) was activated with methanol (#M/4000/PC17, Fisher Chemical) and transfer was performed for 1 h at 200 mA in NuPAGE™ Transfer Buffer (#NP00061, Invitrogen). The membrane was then blocked in 5% (w/v) powdered milk (#T145.2, Carl Roth) for 1 h at RT and incubated with a rabbit anti-human CD70 antibody (1:1000; #69209S, Cell Signaling Technology) and mouse anti-human GAPDH (1:1000; #97166, Cell Signaling Technology) as loading control overnight (O/N) at 4 °C. Next, it was washed three times in TBS-T at RT for a total duration of 1 h. The membrane was incubated with an HRP-linked goat anti-rabbit secondary antibody (#NA9340V, GE Healthcare) diluted 1:2000 in blocking buffer supplemented with 0.2 % (v/v) Tween-20 at RT for 1 h. After a final wash step (performed as above), membrane was incubated with the Clarity Western ECL Substrate (#170-5060, Bio-Rad) following the manufacturer's recommendations. Signal was detected using the Chemidoc imaging station (#17001402, Bio-Rad). The PageRuler™ Plus Prestained Protein Ladder (#26619, Thermo Fisher Scientific) was used as a molecular weight control.

Live-cell confocal microscopy

The 488 nm laser was used to detect EGFP-positive GL261/CD70_EGFP cells and the 555 nm laser to detect tdTomato^{+} effector cells (pMSCV, mCAR27, mCAR27/28, mCAR27/BB). Pixel resolution was 1024x1024 (694.7591x694.7591 microns, with one micron corresponding to 1.4739 pixels). A given pixel comprised 16 bits, the z-range was 15 microns with an interval of 3 microns between two given stacks. The plate was imaged inside a specially-configurated incubator chamber (37 °C, 5% CO_2). Acquired images were analyzed with Fiji (version 1.53c, National Institute of Health). Channels were first split and maximum intensity projections were generated. Afterwards, the brightness and contrast for every channel were adjusted evenly across conditions. For quantification of Gl261/CD70_EGFP signal, the respective channel was converted to binary by using the "*Triangle*" threshold method with dark background. The %-area of the field of view occupied by tumor cells (EGFP signal) was measured for all timepoints and normalized against the first measurement (t=0 min).

Flow Cytometry

For assessment of mCAR-T cell immune cell memory the following mix was used: anti-mouse CD3-BV711 (#100241, Biolegend, RRID: AB_2563945), anti-mouse CD62L-APC (#161217, Biolegend, RRID: AB_3662372), and anti-mouse CD44-FITC (#103005, Biolegend, RRID: AB_312956). For assessment of mCAR-T cell proliferative exhaustion the following mix was used: anti-mouse CD3-BV711 (#100241, Biolegend, RRID: AB_2563945), anti-mouse CD223-BV421 (#125221, Biolegend, RRID: AB_2572080), anti-mouse CD279-FITC (#135214, Biolegend, RRID: AB_10680238), and anti-mouse CD366-APC (#119706, Biolegend, RRID: AB_2561656). The following isotype antibodies were used: PE Mouse IgG3, k Isotype Ctrl Antibody (#556659, BD Biosciences, RRID: AB_396523), BV711 Mouse IgG2a, κ Isotype Ctrl Antibody (#400271,

Biolegend, RRID: AB_3097679), BV421 Mouse IgG1, κ Isotype Ctrl Antibody (#400157, Biolegend, RRID: AB_10897939), BV510 Mouse IgG1, κ Isotype Ctrl Antibody (#400171, Biolegend, RRID: AB_2714004), APC Mouse IgG2b, κ Isotype Ctrl Antibody (#401209, Biolegend, RRID: AB_2941901), PacificBlue Mouse IgG1, κ Isotype Ctrl Antibody (#400151, Biolegend, RRID: AB_2923473), APC Mouse IgG1, κ Isotype Ctrl Antibody (#400119, Biolegend, RRID: AB_2888687), FITC Mouse IgG2a, κ Isotype Ctrl Antibody (#400209, Biolegend, RRID: AB_326458), APC Mouse IgG2b, κ Isotype Ctrl Antibody (#400319, Biolegend, RRID: AB_326500), BV605 Mouse IgG2a, κ Isotype Ctrl Antibody (#400270, Biolegend, AB_3097669), APC Mouse IgG2a, κ Isotype Ctrl Antibody (#40029, Biolegend, RRID: AB_326468), BV421 Mouse IgG1, κ Isotype Ctrl Antibody (#400157, Biolegend, RRID: AB_10897939), PE Rat IgG2b, κ Isotype Ctrl Antibody (#400607, Biolegend, RRID: AB_326551), BV711 Rat IgG2b, κ Isotype Ctrl Antibody (#400653, Biolegend, RRID: AB_3097684), APC Rat IgG2a, κ Isotype Ctrl Antibody (#400512, Biolegend, RRID: AB_2814702), FITC Rat IgG2b, κ Isotype Ctrl Antibody (#400633, Biolegend, RRID: AB_893678), BV421 Rat IgG1, κ Isotype Ctrl Antibody (#400429, Biolegend, RRID: AB_10900998), FITC Rat IgG2a, κ Isotype Ctrl Antibody (#400505, Biolegend, RRID: AB_2736919).

Animal housing, welfare, and experimentation measures

Animals were kept in a pathogen-free facility, maintaining a temperature of 22 ± 2 °C, a humidity level of 55 ± 10%, and a 12-hour light/dark cycle. They were housed in individually ventilated cages (max = 5 animals per cage) and were provided an *ad libitum* standard diet according to the protocols of the German Cancer Research Center (DKFZ). To ensure ethical compliance, only the minimum number of animals necessary to achieve statistical significance was used and every effort was made to reduce animal distress, according to the 3R principles. Animals were monitored daily

throughout the course of all experiments and were terminated upon reaching pre-determined neurological signs, or a body weight loss of 20%, or at the end of the legally approved experimental period. All animal procedures took place at the preclinical research facility of DKFZ. To prevent corneal injury, protective eye cream was applied to the animals' eyes during the orthotopic implantation procedure, bioluminescence imaging, and MRI. To maintain physiological body temperature, all animals undergoing anesthesia were placed on a warming mat with a temperature set at 37°C. Post-surgery pain relief was provided via subcutaneous (s.c.) injection of carprofen (#15, Cp Pharma) at a dose of 10 mg/kg body weight, diluted in a 0.9% (w/v) NaCl solution (#630138, Braun) with a second dose given 24 h later. Carprofen was then administered to the animals via drinking water for 48 h following any surgical intervention. For assessment of human CD70-targeting constructs *in* vivo, a total of 32 NSG animals were used (N=8 animals per treatment group). For assessment of murine CD70-targeting constructs *in vitro* and *in vivo*, a total of 24 C57BL/6J animals were used (N=5 animals per treatment group *in vivo* + N=4 animals as murine T-cell donors for mCAR-T cell production for experimental use). In total, this study utilized N=56 animals. One animal served as one experimental unit. No experimental units or data points were excluded from the analysis. No tumor presence 4 weeks after orthotopic GB cell implantation, based on bioluminescence imaging (BLI), for NSG mice, or 14 days after orthotopic implantation, based on MRI, for C57BL/6J mice, served as an *a priori* exclusion criterion. All procedures on animals of different groups were carried out on the same day, with minimum delay between two given animals. To further reduce bias, we used the same instrumentation, reagents of the same batch, and applied the same data analysis pipelines and parameters for all animals.
