# Supplementary material for: A comparative analysis of CD70-directed CAR-T cells for glioblastoma treatment demonstrates a superior efficacy of the ligand-based construct
Source: Mol Ther Oncol. 2026 Jan 19;34(1):201134. doi: 10.1016/j.omton.2026.201134 (PMC12915223; doi:10.1016/j.omton.2026.201134)
Supplement: Document S2. Article plus supplemental information [file mmc2.pdf]

Molecular Therapy
Oncology
Original Article

A comparative analysis of CD70-directed CAR-T cells for glioblastoma treatment demonstrates a superior efficacy of the ligand-based construct

Alexandros Kourtesakis,[1,2,3] Hiu Nam Hannah Chow,[1,2] Eileen Bailey,[1] Sandra Horschitz,[4] Ammar Jabali,[4] Rainer Will,[5] Christoph Schifflers,[6,7] Abigail K. Suwala,[8] Hannah Rohdjess,[1,2] Melissa Hahn,[1,2,3] Yu-Chan Chih,[9,10] Ling Hai,[1] Denise Reibold,[1] Sonja Pusch,[1] Manuel Fischer,[11] Ralph Sinn,[11] Dennis Alexander Agardy,[9,10] Dirk Carsten Frieder Hoffmann,[1,3] Michael O. Breckwoldt,[11] Robin Wagener,[1,3] Leon Kaulen,[1,3] Philipp Koch,[4] Andreas von Deimling,[8] Lukas Bunse,[9,10] Michael Platten,[9,10] Felix Sahm,[8] Carsten Müller-Tidow,[12] Michael Schmitt,[12] Wolfgang Wick,[1,3] Tim Sauer,[12,13] and Tobias Kessler[1,3,13]

[1]Clinical Cooperation Unit Neurooncology, German Cancer Consortium (DKTK), German Cancer Research Center (DKFZ), 69120 Heidelberg, Germany; [2]Faculty of Biosciences, University of Heidelberg, 69120 Heidelberg, Germany; [3]Department of Neurology and Neurooncology Program, Heidelberg University Hospital, 69120 Heidelberg, Germany; [4]Central Institute of Mental Health, 68159 Mannheim, Germany; [5]Core Facility Cellular Tools, DKFZ German Cancer Research Center, 69120 Heidelberg, Germany; [6]Immunotherapy and Immunoprevention, DKFZ German Cancer Research Center, 69120 Heidelberg, Germany; [7]Cell Biology Research Unit (URBC)–Namur Research Institute for Life Sciences (NARILIS), University of Namur, 5000 Namur, Belgium; [8]German Cancer Research Center (DKFZ), German Consortium for Translational Cancer Research (DKTK), Clinical Cooperation Unit Neuropathology, 69120 Heidelberg, Germany; [9]Clinical Cooperation Unit Neuroimmunology and Brain Tumor Immunology, German Cancer Consortium (DKTK), German Cancer Research Center (DKFZ), 69120 Heidelberg, Germany; [10]Department of Neurology, Medical Faculty Mannheim, Mannheim Center for Translation Neuroscience (MCTN), Heidelberg University, 68167 Mannheim, Germany; [11]Department of Neuroradiology, Heidelberg University Hospital, 69120 Heidelberg, Germany; [12]Medicine, Hematology, Oncology and Rheumatology, University Hospital Heidelberg, 69120 Heidelberg, Germany

CD70, a member of the tumor necrosis factor receptor superfamily, is expressed in glioblastoma (GB), where it promotes tumor growth, migration, and immunosuppression. Accordingly, it has emerged as a target for chimeric antigen receptor (CAR)-T cell therapy. Despite the influence of CAR structure on therapeutic efficacy, no comparative studies have evaluated different CD70-directed CAR designs in GB. Our study addressed this gap. We first validated *CD70* expression in transcriptomic datasets, patient tissue, and GB cell lines. We then generated CD70-specific CAR-T cells featuring distinct target recognition and co-stimulatory domains (CD27z, LF28z, and LFBBz) and performed phenotypic characterization. Using co-culture systems and 3D cerebral organoids, we showed that all constructs eliminated target cells in a CD70-dependent manner, with CD27z secreting the highest levels of Th1 cytokines. This functional advantage translated into superior survival in an orthotopic GB mouse model. Based on these findings, we developed a panel of murine CD27-based constructs, all of which demonstrated potent antitumor activity *in vitro* and in immunocompetent GB mouse models, further underscoring the therapeutic promise of CD27 integration into the CAR design. Collectively, our comparative analysis highlights the superior efficacy of the ligand-based construct, supporting its incorporation into a clinical trial targeting CD70 in recurrent GB.

INTRODUCTION

Chimeric antigen receptor (CAR)-T cell therapy has yet failed to bring long-term clinical benefit to glioblastoma (GB) patients. Recent GB trials suggested promising CAR-T cell antitumor efficacy,[1,2] but observed responses were transient, underlining the need for further studies. In recent years, attention has been drawn to CD70, a member of the tumor necrosis factor receptor superfamily.[3] CD70 is detected in GB, where the highest expression levels are reported in the mesenchymal subtype.[4,5] Notably, a negative prognostic impact of CD70 expression on overall patient survival in low-grade glioma (LGG) and GB has been reported, attributed to its pro-tumorigenic function.[4] Specifically, CD70 expression on GB cells led to a shift of the secretome toward a more immunosuppressive state[4] and is associated with enhanced cancer cell migration and the expression of *CD44* and *SOX2*, factors linked to disease progression.[6] Moreover, it is associated with a higher incidence of $CD163^+$ tumor-associated macrophages in GB patients[6] and is suggested to promote GB self-renewal, stemness and tumor growth.[7] Finally, it

Received 4 August 2025; accepted 8 January 2026;
https://doi.org/10.1016/j.omton.2026.201134.

[13]These authors contributed equally

Correspondence: Tobias Kessler, MD, Neurology Clinic and Neurooncology Program at the National Center for Tumor Diseases & DKTK, DKFZ, Im Neuenheimer Feld 400, 69120 Heidelberg, Germany.
E-mail: t.kessler@dkfz.de

Published by Elsevier Inc. on behalf of The American Society of Gene and Cell Therapy.
This is an open access article under the CC BY license (http://creativecommons.org/licenses/by/4.0/).

has been shown to induce T cell apoptosis,[4,8] possibly via recruitment of pro-apoptotic protein Siva that is shown to inflict mitochondrial damage and caspase activation,[9,10] or through the well-described FasL/Fas axis.[11] Based on the above, GB CD70 has been successfully targeted with CAR-T cells in the preclinical setting,[4,7,12] and two clinical studies utilizing IL-8 receptor-modified CD70-directed CAR-T cells for GB and pediatric high-grade glioma treatment have been initiated (NCT05353530 and NCT06946680, respectively).[12]

Despite the feasibility of targeting CD70 with CAR-T cells in GB, a comparative analysis of different CD70-directed CAR constructs is lacking. This is critical since the CAR composition significantly influences its metabolic program and antitumor cytotoxicity, resulting in varying treatment outcomes in patients treated with different constructs.[13–15] Consequently, such a study is pivotal to determine the construct with the most optimal target engagement, killing specificity and persistence for clinical translation. In our study, we first independently validated CD70 as a suitable therapeutic target in GB and then performed an *in vitro* and *in vivo* comparative analysis of different anti-CD70 CAR constructs, both single-chain variable fragment (scFV)- and ligand-based, by employing conventional coculture, 3D cerebral organoid platforms and finally, patient-derived orthotopic xenograft GB mouse models. We demonstrate that all constructs were able to yield specific target-dependent antitumor responses *in vitro* and *in vivo* and showcase the superiority of the CD27z construct. Expanding on these findings and acknowledging the importance of the endogenous immune system in CAR-T cell efficacy, we advanced the CD27-based design and generated a novel panel of murine CD70-directed CAR-T cells incorporating mCD27 along with distinct co-stimulatory domains. These constructs mirrored the functionality of their human CD27z counterpart, effectively eliminating murine GB cells *in vitro* and *in vivo*, with notable long-term *in vivo* persistence, further underscoring the translational relevance and robustness of the CD27-based design.

RESULTS

Validation of CD70 as a therapeutic target in GB

On a single cell level, we first explored *CD70* expression in an in-house dataset comprising patients diagnosed with GB ($N = 16$), isocitrate dehydrogenase-1-mutant glioma ($N = 3$) and pediatric high-grade glioma ($N = 1$, all patients newly diagnosed, Table S1), and discovered heterogeneous *CD70* expression in the malignant cell fraction (Figure 1A).[16] *CD70* was also expressed in myeloid cells. Nevertheless, the myeloid cluster almost universally expressed high levels of immunosuppressive markers *IL10* and *TGFB1*,[17] but also *CD163*, a receptor strongly expressed in immunosuppressive TAMs.[6,18,19] *CD70* expression was also detected in T cells, as previously described by others.[20–22] Nevertheless, this cluster highly co-expressed *TGFB1* and notably *FOXP3*, a marker of T_{reg} cells, also with immunosuppressive functions in GB.[17] These results were recapitulated in an independent published single-cell dataset,[23] analyzed using the Broad Institute Single Cell Portal.[24] Specifically, *CD70* was heterogeneously expressed in GB cells, but also in myeloid- and T cells, with the last two populations co-expressing the aforementioned immunosuppressive factors (Figure S1A). This suggests that targeting CD70 with CAR-T cells can not only eliminate $CD70^+$ GB cells in qualifying patients but also potentially deplete suppressive immune populations, an approach reported in other studies.[25,26] This might eventually reshape the tumor microenvironment toward a more immune-privileged state, improving treatment outcomes. Furthermore, by analyzing Human Protein Atlas cell line data,[27] we revealed *CD70* expression in 86.6% (52 of 60) of glioma cell lines (Figure S1B; expression cutoff $\geq$1 nTPM) with 33.3% (20 of 60) cell lines displaying higher levels (expression cutoff $\geq$50 nTPM). We then explored *CD70* expression in primary and recurrent GB patient samples from a Heidelberg University Hospital cohort by means of bulk RNA sequencing (RNAseq).[28] *CD70* was expressed in 39.8% (76 of 190) of samples analyzed (Figure 1B, expression cutoff $\geq$1 FPKM). We did not observe differences in *CD70* expression levels between samples derived at initial diagnosis and recurrent tumors. The same observation was obtained when analyzing a subset of 20 matched primary/recurrent pairs from the same dataset (Figure 1C). All these patients received radiotherapy and at least one line of systemic treatment between the two sample acquisitions. These findings lie in agreement with previous research showing a trend toward increased *CD70* expression in recurrent GB, with reported differences not meeting statistical significance.[4,7] Next, we confirmed CD70 expression by immunohistochemistry (IHC) in tumor lesions of three representative GB patient samples from the same cohort, with confirmed *CD70* positivity in bulk RNAseq (Figure 1D). Importantly, CD70 was expressed in tumor cells and did not co-localize with CD45, a global immune cell marker. Next, we screened a panel of primary and conventional cell lines for CD70 and detected expression in a subset by flow cytometry (Figure 1E) and qPCR (Figure 1F). CD70 expression was heterogeneous, with P3, T325, T269, U3017, and BG7 cell lines lacking relevant expression, U3021 and BG5 showing low expression, and U138 and U3047 displaying the highest levels. A strong positive correlation between *CD70* gene expression and protein levels was revealed (Figure 1G). To first test the CD70-directed CAR-T cell specificity, we performed a lentiviral overexpression (OE) of *CD70* in zero-expressing cell lines P3 (P3/CD70), S24 (S24/CD70), and T269 (T269/CD70), which was confirmed by flow cytometry (Figure 1H) and western blot (Figure S1C).

Generation and phenotyping of CD70-directed CAR-T cells

CD70-targeting CAR constructs were designed as previously described.[29] As target recognition domain, two constructs featured the scFv of an anti-CD70 antibody, followed by a long-flexible spacer motif and the transmembrane domain of CD28. Fused to that, one construct contained the cytoplasmic domain of CD137 (4-1BB) while the other one carried the equivalent domain of CD28. The third vector consisted of the full length CD27 (the only natural ligand of CD70). All constructs carried the intracellular signaling domain of CD3z chain. For detection, the tdTomato fluorescent reporter, driven by an IRES element, was cloned downstream of every CAR (Figure 2A). A non-targeting construct was also generated by

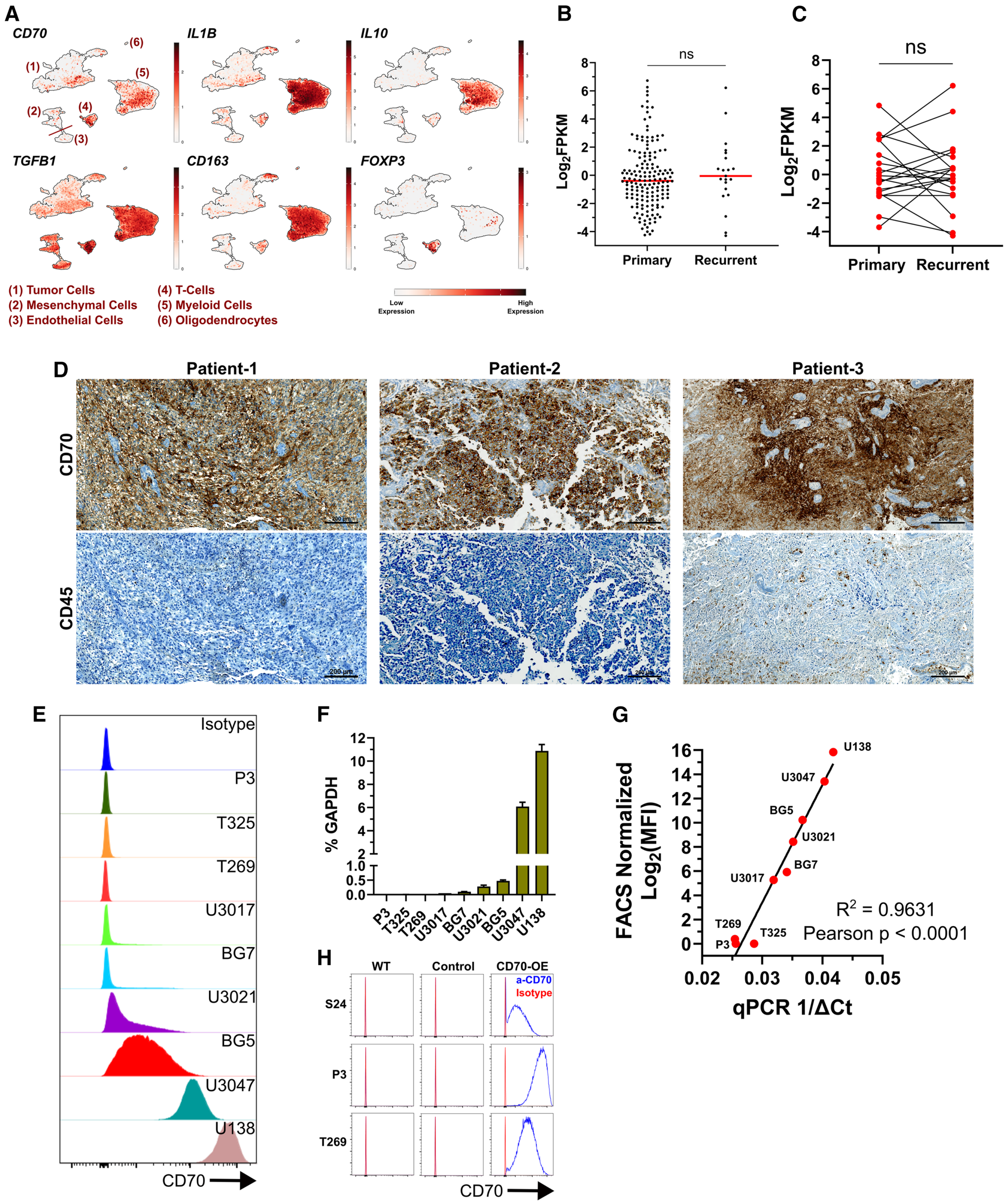

Figure 1. Validation of CD70 as a GB target for CAR-T cell therapy

(A) Expression of *CD70* and selected markers from an in-house glioma patient single-cell RNA seq dataset. (B) *CD70* expression in GB samples from Heidelberg University Hospital, analyzed by bulk RNAseq. Each dot represents a patient. A Mann-Whitney test was performed to assess statistical significance. (C) *CD70* expression in 20 matched pairs from (B). A two-tailed paired *t* test was used to assess significance. (D) IHC staining of three Heidelberg University Hospital GB patients from (B) with high *CD70* expression based on RNAseq. Scale bars, 200 μm. Patient-1 and Patient-3: primary GB; Patient-2: recurrent GB. (E) Measurement of CD70 protein levels on GB cells by flow cytometry. (F) Measurement of *CD70* gene expression levels in glioma cell lines by RT-qPCR. *N* = 3 technical replicates per cell line. (G) Correlation between *CD70* gene expression and protein levels from (E) and (F). (H) Measurement of CD70 on the surface of generated primary GB models by flow cytometry (blue histograms). An isotype control antibody (red histograms) was used. For (E) and (H), data were gated on single live cells. Data presented as mean (SD). $^*p < 0.05$, $^{**}p < 0.01$, $^{***}p < 0.001$, $^{****}p < 0.0001$; n.s., not significant.

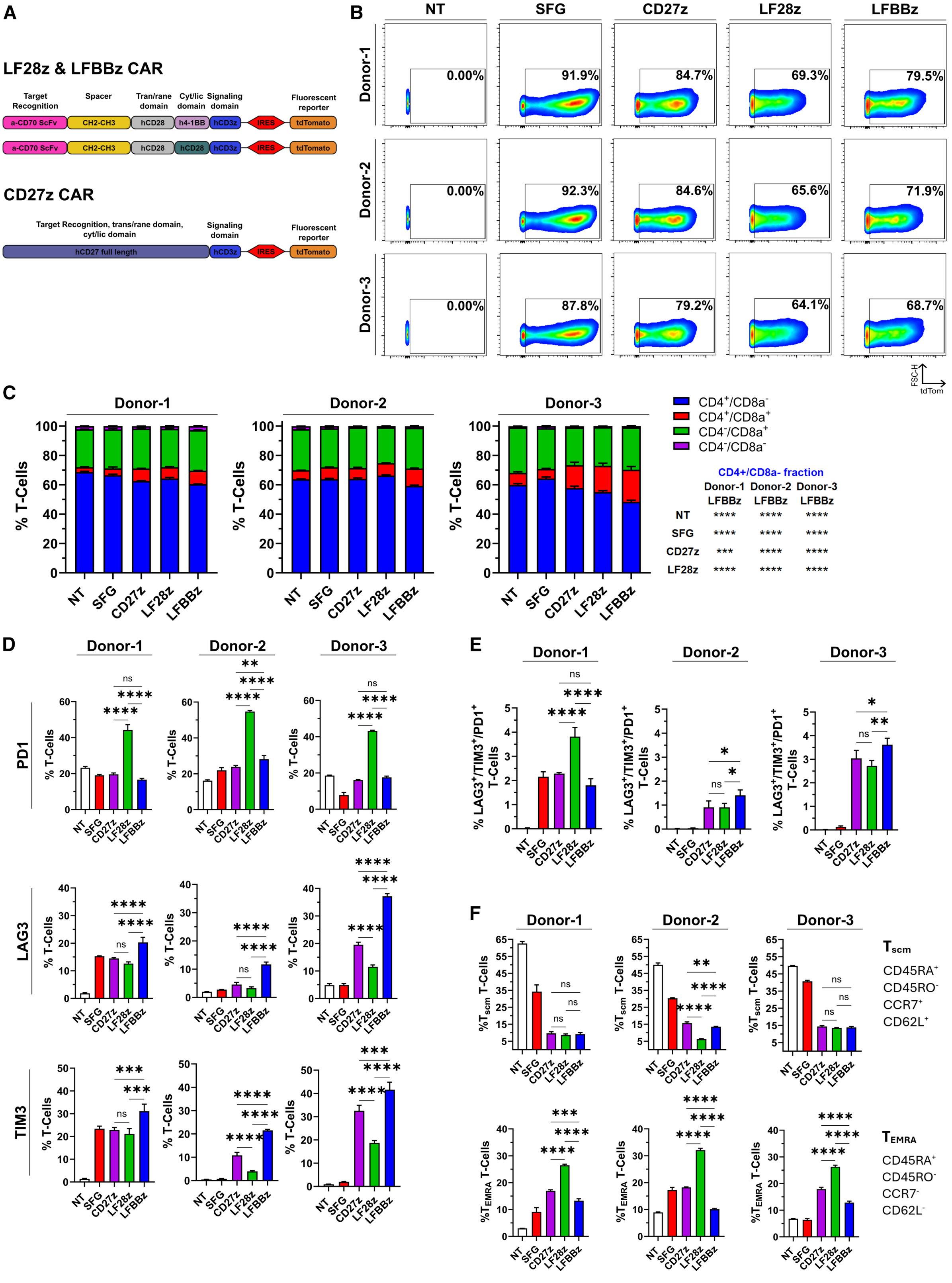

(legend on next page)

replacing the CAR sequence with a tdTomato cassette (SFG construct). Constructs were used to produce retrovirus and activated T cells from healthy donors were transduced. CAR expression was determined 4 d after transduction by flow cytometry (Figure 2B). At the same time point, we also performed phenotypic characterization of our target-naïve effector cells. CAR-T cells featuring the 4-1BB co-stimulatory domain showed the lowest fraction of T_{helper} cells ($CD4^+/CD8a^-$; Figure 2C). Additionally, we discovered that PD1 levels were consistently elevated in the LF28z construct while on the other hand, CAR-T cells featuring the 4-1BB co-stimulatory domain expressed higher levels of LAG3 and TIM3 (Figure 2D). Notably, the fraction of CAR-T cells that expressed PD1, LAG3, and TIM3 simultaneously was low for all constructs and donors (Figure 2E). Further phenotyping for immune memory markers CD45RA, CD45RO, CCR7, and CD62L revealed that LF28z CAR-T cells featured the highest frequency of terminal effector memory (T_{EMRA}) cells, a phenotype linked to higher cytotoxicity and glycolytic metabolism capacity but also reduced proliferation and persistence.[13–15] Concerning stem cell memory (T_{SCM}) cells, the CD27z construct featured the highest fraction in one donor (Figure 2F).

CD70-directed CAR-T cells effectively target GB cells *in vitro*

To evaluate the potency of anti-CD70 CAR-T cells *in vitro*, we co-cultured them with the generated GB models in the absence of exogenous cytokines. After 18 h, all CD70 CAR-T cells secreted significantly higher levels of TNF-α, IFN-γ, and Granzyme-B when co-cultured with CD70-OE GB cells compared to mock-transduced T cells, as determined by ELISA (Figures 3A and S2A). Importantly, CD27z CAR-T cells secreted the highest levels of TNF-α and IFN-γ in all conditions. To discover whether this recognition was accompanied by tumor cell eradication, we co-cultured CAR-T cells with P3/CD70_EGFP tumor cells and monitored tumor cell growth and Annexin-V binding in real time using the Incucyte instrument. Supporting the cytokine secretion patterns, only CD70-directed CAR-T cells eliminated GB cells (Figure 3B). Co-culture with LF28z CAR-T cells led to the highest degree of Annexin-V binding and the most prominent short-term reduction of tumor cell signal in all donors (Figures 3B and S2B), possibly attributed to the higher fraction of cytotoxicity-associated T_{EMRA} cells (Figure 2F), but also to the near-zero fraction of $PD1^+/LAG3^+/TIM3^+$ cells in this construct, detected by flow cytometry after co-culture (Figure S3A). In the same setting, CAR-T cell engagement with tumor CD70 led to significantly reduced $PD1^-/LAG3^-/TIM3^-$ fractions compared to the control construct (Figure S3B), suggesting that target interception might lead to exhaustion. The anti-glioma cytotoxicity of CD70-targeting CAR-T cells was also demonstrated in naturally CD70 expressing cell lines. Specifically, we utilized $CD70^+$ primary GB cell line MMKI (Figure 4A).[30–32] After overnight (O/N) co-culture with CD70-targeting CAR-T cells in a 2:1 effector:target ratio, we observed specific CAR-T cell activation, delineated by upregulation of co-stimulatory marker CD137 (4-1BB) on CAR-T cells compared to control cells (Figure 4B).[33] CD27z CAR-T cells showed the most prominent activation, in line with their higher cytokine secretion pattern (Figure 3A). The CAR-T cell specificity was also interrogated in a gene KO setting. We co-cultured CAR-T cells with genetically modified U138 cells that lacked *CD70* expression after successful CRISPR/Cas9-mediated gene knockout (U138/CD70_KO), and with U138 cells transduced with a control non-targeting oligonucleotide sequence (U138/Ctrl; Figure 4C). At an effector:target ratio of 1:1, only CD70-expressing U138/Ctrl cells were effectively eliminated by CAR-T cells, as determined by live-cell imaging in the Incucyte platform (Figure 4D). Taken together, we highlight the potency of CD70-targeting CAR-T cells to eradicate primary GB cell lines in a target-dependent manner. In our system, CD27z CAR-T cells showed the highest activation while LF28z CAR-T cells performed faster short-term killing of GB cells.

CD70-specific CAR-T cells eliminate GB cells in cerebral organoids

To elaborate on the CAR-T cell potency in a more complex 3D setting requiring effector cell penetration for GB cell elimination, we investigated whether the observed anti-glioma cytotoxicity could be recapitulated in *in vitro*-derived cerebral organoids.[34,35] Initially, we co-cultured cerebral organoids with EGFP-expressing primary GB cell lines P3/MCS, P3/CD70, T269/MCS, and T269/CD70, and demonstrated successful tumor cell infiltration and CD70 expression 14 d later by immunofluorescence (IF; Figures 5A and 5B). Importantly, CD70 was not expressed endogenously by cerebral organoids, which could potentially result in CAR-T cell-mediated on-target/off-tumor toxicity (Figure 5C). Next, we co-cultured cerebral organoids with our GB models for 14 d and subsequently treated the GB-infiltrated organoids with CAR-T cells. Successful infiltration of organoids by CAR-T cells was demonstrated 3 d later by IF (Figure 5D). This infiltration was more prominent in the condition where tumor cells expressed CD70 and was coupled to a potent anti-GB effect. Particularly, we observed lower tumor cell signal and significantly elevated levels of Granzyme-B, determined by IF, in treated organoids previously infiltrated by $CD70^+$ cells, compared to treated organoids previously invaded by $CD70^-$ GB cells

Figure 2. Generation and phenotyping of anti-CD70 CAR-Tcells

(A) CAR construct design. (B) Transduction efficiency of primary human T cells by flow cytometry. A non-transduced (NT) sample from each donor was used to determine gating. Data gated on single live $CD3^+$ cells. (C) CD8a/CD4 expression on transduced T cells, determined by flow cytometry. A one-way ANOVA followed by a Dunnett's multiple comparisons test was performed to assess significance. (D) Expression of exhaustion receptors on transduced T cells, determined by flow cytometry. (E) Quantification of $PD1^+/LAG3^+/TIM3^+$ T cells from (D). (F) Expression of immune memory-associated markers on transduced T cells by flow cytometry. For (C), (D), (E), and (F), $N = 3$ biological replicates per group. Isotype controls were used to determine gating and displayed data are gated on single live $CD3^+$ cells (NT) or single live $CD3^+$/$tdTomato^+$ cells (SFG, CD27z, LF28z, and LFBBz). Data presented as mean (SD). A one-way ANOVA followed by a post-hoc Šídák's multiple comparisons test was performed. $^*p < 0.05$, $^{**}p < 0.01$, $^{***}p < 0.001$, $^{****}p < 0.0001$; n.s., not significant.

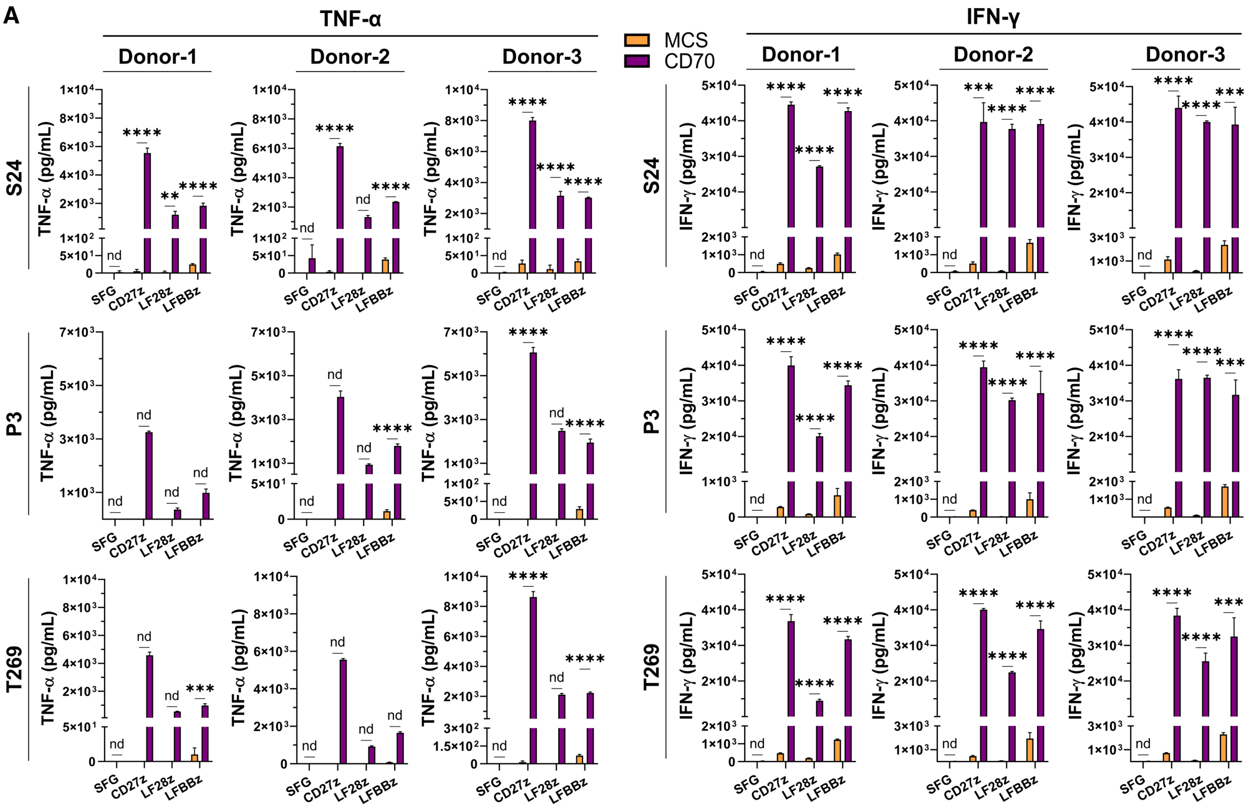

	S24/CD70			P3/CD70			T269/CD70		
	Donor-1	Donor-2	Donor-3	Donor-1	Donor-2	Donor-3	Donor-1	Donor-2	Donor-3
CD27z vs LF28z	****	****	****	****	****	****	****	****	****
CD27z vs LFBBz	****	****	****	****	****	****	****	****	****
LF28z vs LFBBz	*	****	ns	****	***	**	**	****	ns

	S24/CD70			P3/CD70			T269/CD70		
	Donor-1	Donor-2	Donor-3	Donor-1	Donor-2	Donor-3	Donor-1	Donor-2	Donor-3
CD27z vs LF28z	****	ns	ns	****	*	ns	****	****	**
CD27z vs LFBBz	*	ns	ns	**	ns	ns	***	**	ns
LF28z vs LFBBz	****	ns	ns	****	ns	ns	****	****	ns

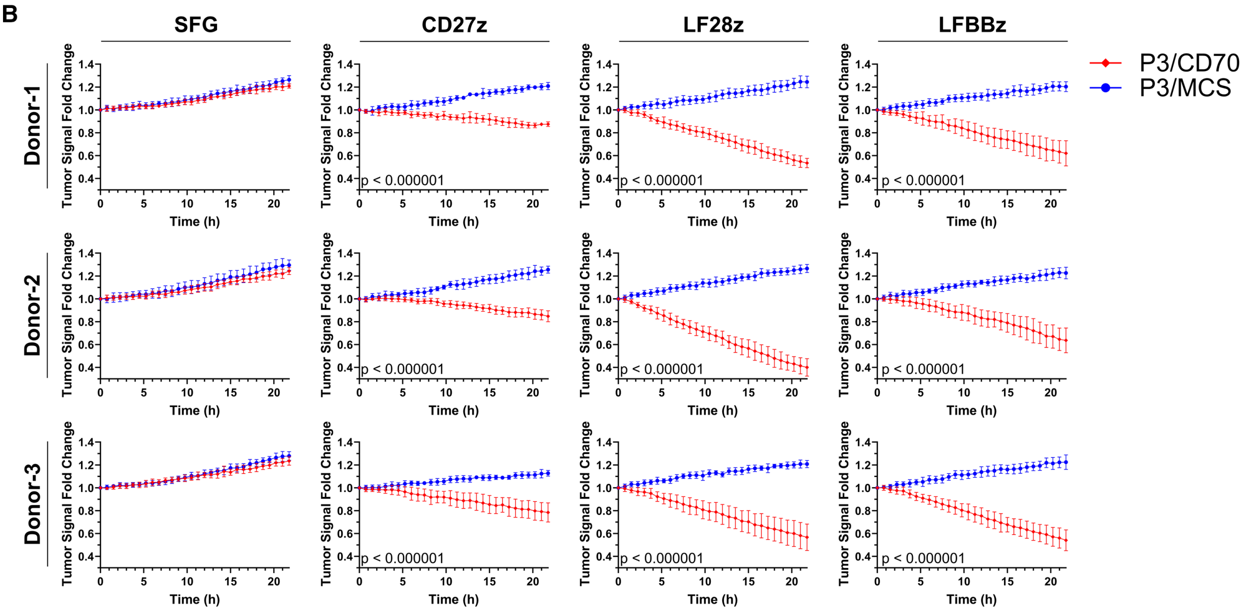

(legend on next page)

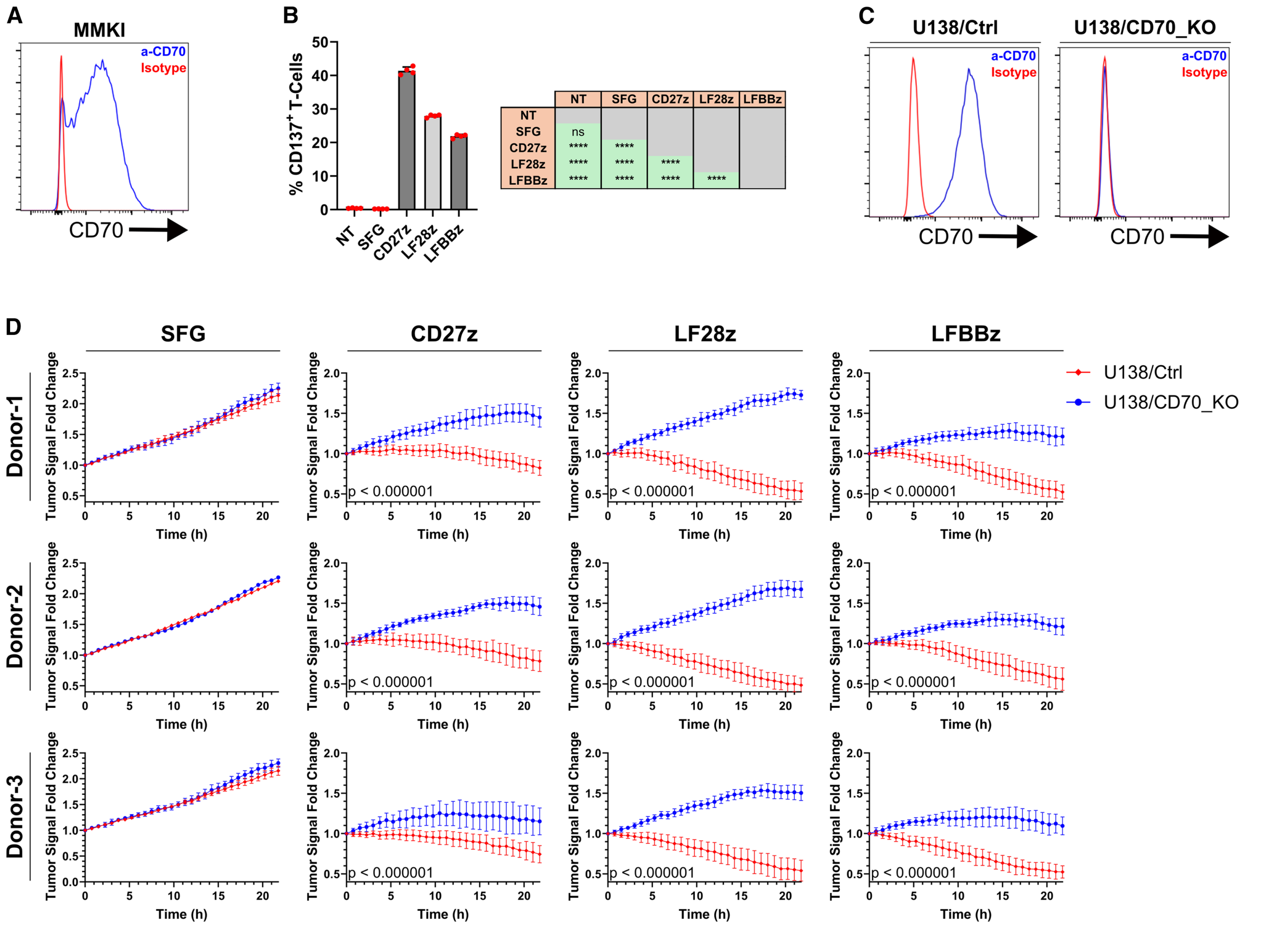

Figure 4. Assessment of anti-CD70 CAR-T cell killing in naturally CD70 expressing cell lines

(A) CD70 expression levels (blue histogram) on primary GB cell line MMKI by flow cytometry. Data gated on single live cells. An isotype control (red histogram) was used. (B) Quantification of activation marker CD137 after O/N co-culture of MMKI cells with CD70-targeting CAR-T cells by flow cytometry. $N = 4$ biological replicates per group. Data gated on single live $CD3^+$ cells (NT) or single live $CD3^+$/$tdTomato^+$ cells (SFG, CD27z, LF28z, and LFBBz). Isotype control antibodies were used for gating. A one-way ANOVA followed by a Tukey's multiple comparisons test was applied for significance. (C) Measurement of CD70 (blue histograms) on the surface of generated genetic knockout models by flow cytometry. An isotype control (red histogram) was used. Data gated on single live $EGFP^+$ cells. (D) Measurement of tumor cell signal during co-culture with CAR-T cells on the Incucyte platform. $N = 2$ biological replicates per group. Every biological replicate is the mean of $N = 5$ technical replicates. Time point intervals = 45 min. Statistical significance was assessed as in Figure 3B). For (B) and (D), data are presented as mean (SD). $^*p < 0.05$, $^{**}p < 0.01$, $^{***}p < 0.001$, $^{****}p < 0.0001$; n.s., not significant.

(Figures 5D and 5E). Corroborating this, Granzyme-B and IFN-γ levels in the SN of these co-cultures were significantly increased in all $CD70^+$ GB conditions (Figure 5F). Direct comparisons among constructs revealed that in all timepoints and for both P3/CD70 and T269/CD70 cell lines, CD27z CAR-T cells secreted the highest levels of Granzyme-B (Figures 5F and 5G). The same trend was observed for IFN-γ release (Figure 5G). Conclusively, we illustrate the capability of CD70-directed CAR-T cells to infiltrate cerebral organoids previously invaded by primary GB cell lines to exert cytotoxic effector functions, which are strictly driven by GB CD70. In this setting, CD27z CAR-T cells secreted the highest levels of measured cytokines.

Figure 3. *In vitro* assessment of the CD70-directed CAR-T cell anti-GB cytotoxicity

(A) Measurement of secreted TNF-α and IFN-γ in the SN of GB/CAR-T cell co-cultures by ELISA. $N = 3$ biological replicates per group. For comparison between MCS and CD70 (upper bar plots), an unpaired two-tailed t test was used. For comparisons among constructs (bottom), a one-way ANOVA followed by a Holm-Šídák multiple comparisons test was used. (B) Measurement of tumor cell signal during co-culture with CAR-T cells on the Incucyte platform. $N = 2$ biological replicates per group. Every biological replicate is the mean of $N = 5$ technical replicates. Time point intervals = 45 min. A two-tailed Student's t test was performed using the values of the last measured time point to determine statistical significance. For (A) and (B), data presented as mean (SD). $^*p < 0.05$, $^{**}p < 0.01$, $^{***}p < 0.001$, $^{****}p < 0.0001$; n.s., not significant.

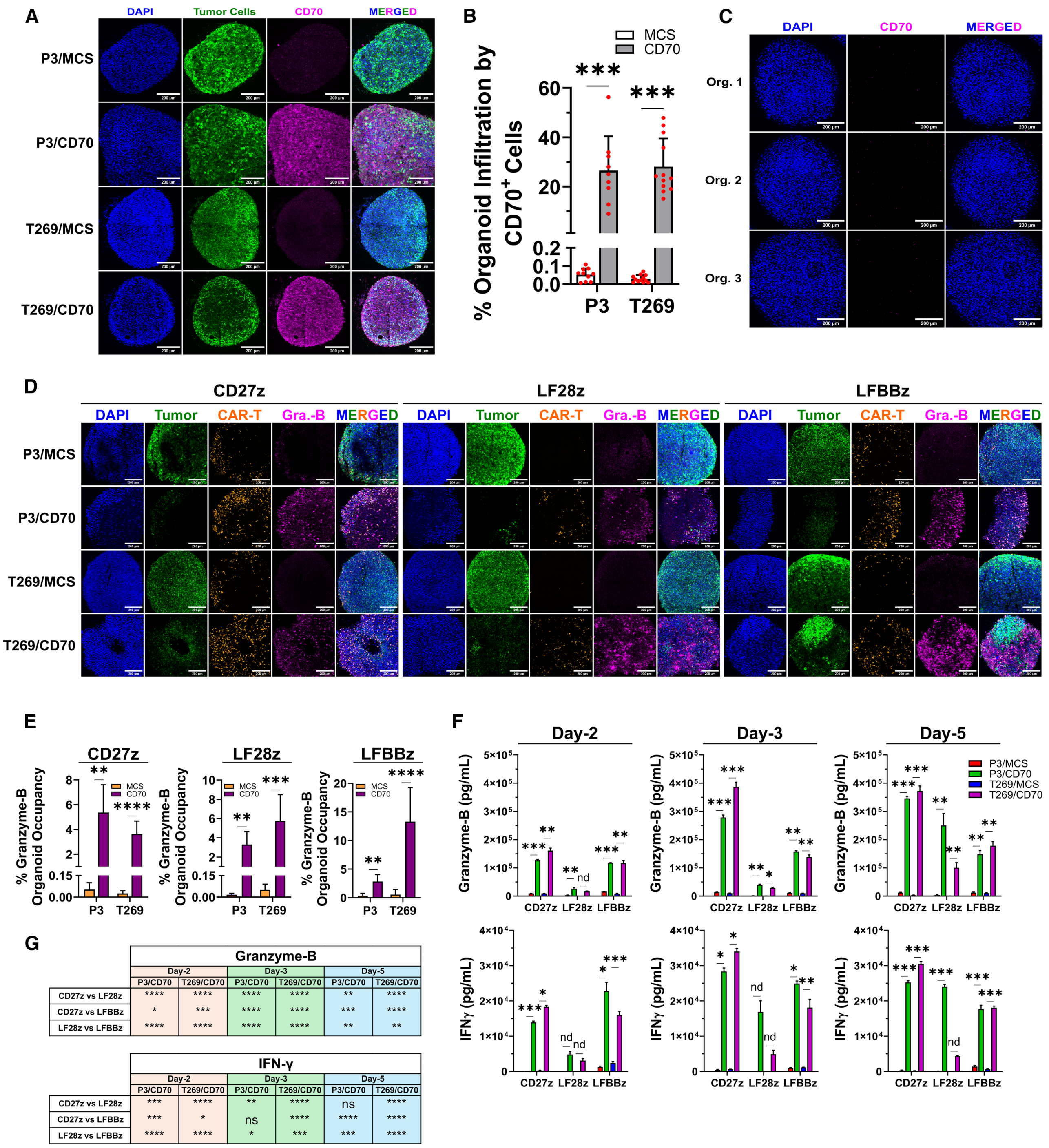

Figure 5. Evaluation of CD70-directed CAR-T cell effector function in cerebral organoids

(A) Confocal microscopy of cerebral organoids, infiltrated by generated GB models. (B) Quantification of CD70 signal in organoids from (A). Each dot represents an organoid. A Welch's *t* test was used to assess significance. (C) Immunofluorescence analysis of endogenous CD70 expression in cerebral organoids. (D) Confocal microscopy of cerebral organoids previously invaded by GB cells and subsequently treated with CAR-T cells for 3 d. (E) Quantification of Granzyme-B signal from (D). A two-tailed *t* test was used to determine significance. (F) Measurement of secreted Granzyme-B and IFN-γ levels in the SN of co-cultures from (D) by ELISA. $N = 3$ biological replicates per group. (G) CAR construct direct comparisons from (F). A one-way ANOVA followed by a Tukey's post hoc test for multiple comparisons was used. For (A), (C), and (D), scale bars, 200 μm. For (D) and (E), $N \geq 3$ organoids per group. For (E) and (F), a two-tailed *t* test was used to assess significance. For (B), (E), and (F), data presented as mean (SD). *$p < 0.05$, **$p < 0.01$, ***$p < 0.001$, ****$p < 0.0001$; n.s., not significant.

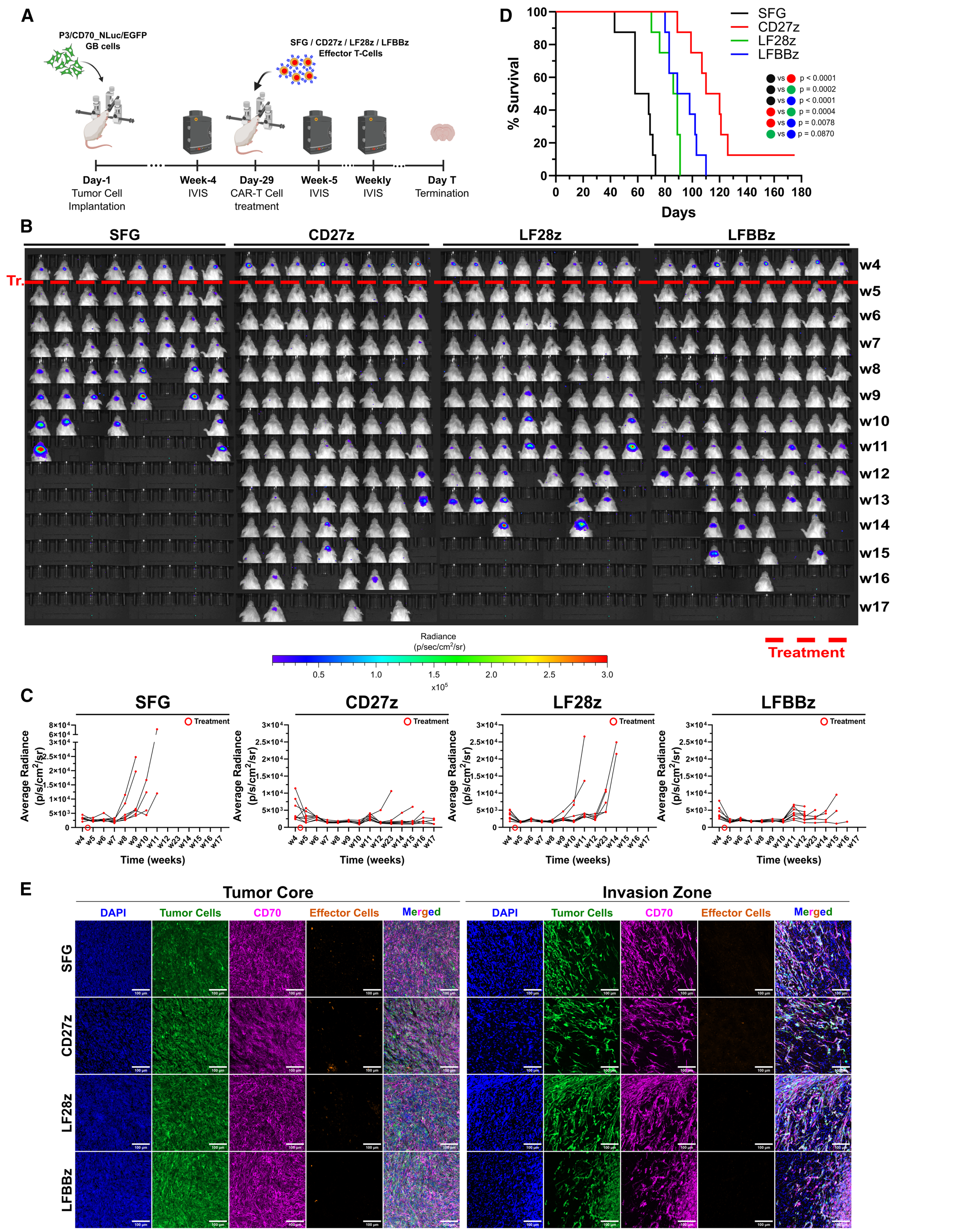

(legend on next page)

CD27z CAR-T cells show superior *in vivo* activity against orthotopic GB

Next, the ability of our CD70 CAR constructs to eliminate $CD70^+$ GB in an orthotopic murine model was investigated. To this end, we first genetically modified the P3/CD70 cell line to express nano-luciferase (NLuc) and EGFP (P3/CD70_NLuc/EGFP), to allow GB monitoring by bioluminescence imaging (BLI) on a weekly basis. EGFP expression was verified by flow cytometry (Figure S4A) and the functionality of the NLuc gene was validated in an *in vitro* furimazine assay (Figure S4B). We orthotopically implanted NSG mice with 10^5 P3/CD70_NLuc/EGFP cells (day-1) and verified the presence of GB tumors 4 weeks later by BLI (day-28; Figures 6A and 6B). Subsequently, animals were assigned to different groups based on the size of their tumors to harmonize the tumor burden among the groups prior to treatment initiation (Figure S4C; $N = 8$ animals per group). Investigators were blinded during the conduct of the experiment. On day-29, animals were treated with 2×10^6 effector cells intratumorally (ICT). Mouse overall survival served as the primary outcome measure. CAR-T cell treatment, but not the infusion of SFG T cells, led to tumor remission in all treated animals (Figures 6B and 6C). Six weeks after therapy (w10), all mice treated with CD27z CAR-T cells showed no signs of tumor, while only 1/8 and 3/8 mice treated with LFBBz and LF28z CAR-T cells, respectively, showed recurring tumors, based on BLI. In comparison to the control group, all CD70-targeting CAR-T cell constructs significantly extended survival (CD27z median survival 115 days vs. SFG median survival 63 days, HR = 0.03679, 95% CI: 0.007695–0.1759, $p < 0.0001$; LF28z median survival 87.5 days vs. SFG median survival 63 days, HR = 0.05947, 95% CI: 0.01353–0.2614, $p = 0.0002$; LFBBz median survival 93.5 days vs. SFG median survival 63 days, HR = 0.03679, 95% CI: 0.007695–0.1759, $p < 0.0001$; Figure 6D). While there was no significant difference in survival between the LF28z and the LFBBz constructs (HR = 0.3407, 95% CI: 0.09927–1.169, $p = 0.0870$), treatment with CD27z CAR-T cells led to a superior survival of treated animals compared to the other two CAR constructs (CD27z vs. LF28z HR = 0.07339, 95% CI: 0.01722–0.3127, $p = 0.0004$; CD27z vs. LFBBz HR = 0.1756, 95% CI: 0.04878–0.6320, $p = 0.0078$; Figure 6D). Eventually, all mice, except one treated with CD27z CAR T cells, experienced tumor recurrence and succumbed to the disease. IF analysis revealed CAR-T cell scarcity in recurrent tumor tissue, suggesting that recurrence might have emerged due to suboptimal long-term CAR-T cell persistence. This finding was also accompanied by universal and high CD70 expression on tumor cells, providing a clear rationale for a second local CAR-T cell administration to constrain GB outgrowth and further extend survival (Figure 6E). Conclusively, we demonstrate the capacity of CD70-targeting CAR-T cells to exert anti-GB functions *in vivo*, and that the CD27z construct performed best concerning OS prolongation in treated animals.

A panel of murine ligand-based constructs demonstrates efficacy against murine GB *in vitro*

Building upon these results and considering the critical role of the endogenous immune system in shaping CAR-T cell responses, we further advanced the superior CD27-based design by developing a new panel of murine CD70-targeted CAR-T cells featuring mCD27 and diverse co-stimulatory domains. For comparison purposes, we first included a previously described construct comprising full-length mCD27 fused to the cytoplasmic tail of mCD3z (referred to as mCAR27). Additionally, we generated two other constructs, both incorporating full-length mCD27 as the target recognition, transmembrane, and co-stimulatory domain. One construct additionally included a mCD28 cytoplasmic domain (mCAR27/28), while the other contained the murine equivalent of CD137 (mCAR27/BB; Figure 7A). For detection purposes, a tdTomato cassette was cloned downstream of each CAR, driven by a T2A self-cleavage site. As a control, a non-targeting retroviral vector encoding tdTomato alone was also generated (pMSCV). Primary activated murine T cells were successfully transduced with produced retrovirus (Figure 7B). Across all constructs and donors, substantially higher fractions of cytotoxic $CD8a^+/CD4^-$ cells were reported, suggesting an elevated killing potential (Figures S5A and S5B). Phenotypically, mCAR27 cells featured the highest fraction of effector-like memory cells (Figures S6A and S6B) and the lowest incidence of PD1/LAG3/TIM3 triple negative cells (Figures S6C and S6D). To assess mCAR-T cell functionality, *mCD70* was overexpressed in murine glioma cell lines GL261 and CT2A (Figures 7C and 7D). Upon O/N co-culture with the engineered mCD27-based mCAR-T cells, we observed mCD70-dependent secretion of TNF-α (Figures 7E and 7F) and effector cell activation (Figure S7). Notably, murine T cells transduced with the mCAR27 construct consistently exhibited higher levels of TNF-α secretion and activation compared to the mCAR27/28 and mCAR27/BB variants across all mouse donors and target murine GB cell lines, an observation potentially linked to the enriched effector-like memory phenotype observed in this construct (Figure S6A). The cytotoxic potential of all mCD27-based constructs was further validated in a live-cell time-lapse co-culture assay (Figure 7G), where all mCD70-targeting mCAR-T cells, unlike mock-transduced murine T cells, effectively eliminated GL261/CD70_EGFP cells (Figures 7H and S8).

Figure 6. *In vivo* evaluation of the anti-CD70 CAR-T cell killing efficacy

(A) *In vivo* pipeline. (B) Measurement of tumor cell signal in mice orthotopically implanted with P3/CD70_NLuc/EGFP cells and subsequently treated ICT with anti-CD70 CAR-T cells by BLI.

(C) Quantification of BLI signals from B). $N = 8$ animals per group.

(D) Overall survival of treated mice from B). The log rank (Mantel-Cox) test was used to assess statistical significance. Bonferroni-adjusted significance threshold: $p = 0.0083$.

(E) Analysis of CAR-T cell persistence and CD70 expression in recurrent tumors of treated mice from (A) by IF. Representative images from $N = 3$ animals per group. Scale bars, 100 μm.

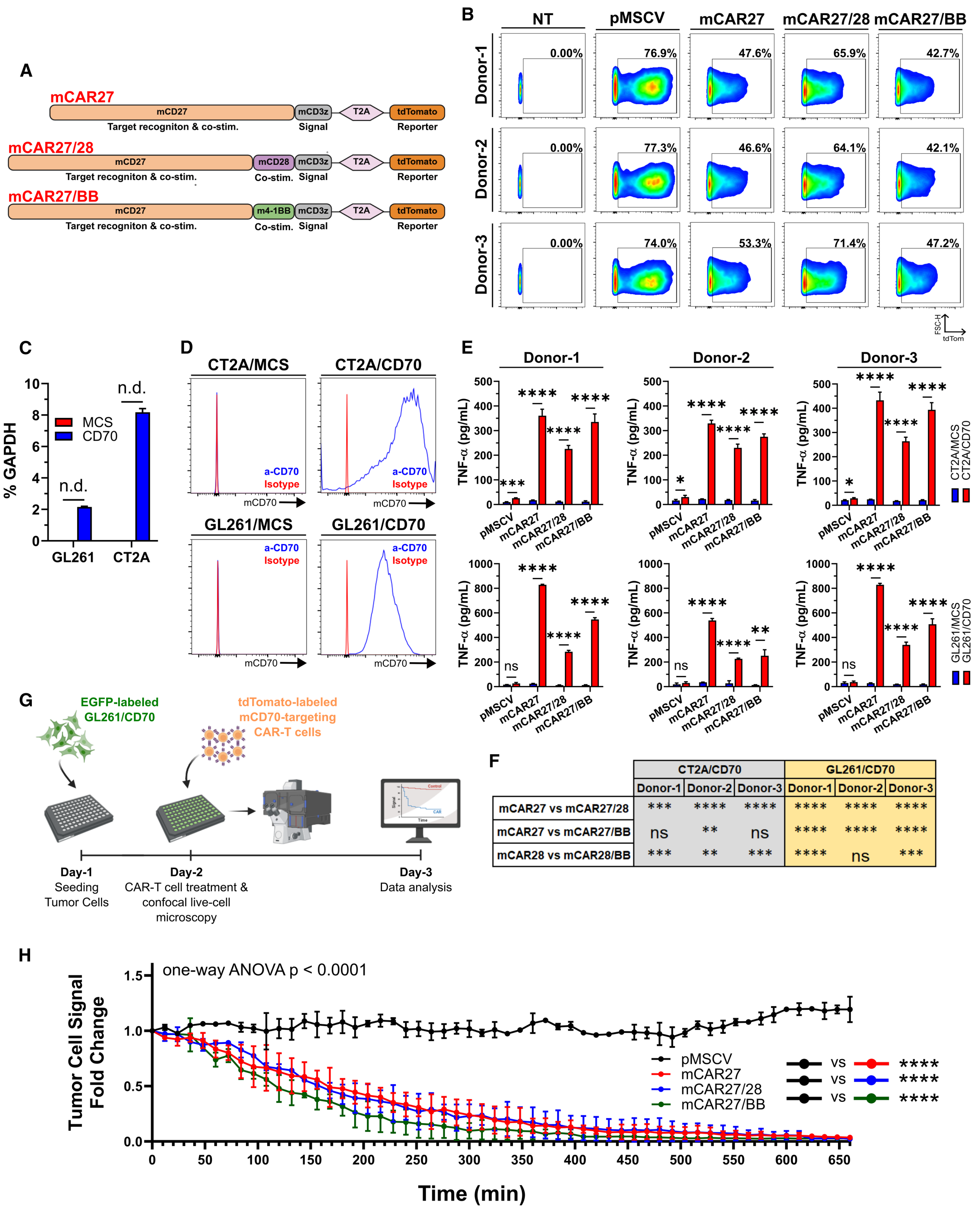

(legend on next page)

Ligand-based mCD70-targeting CAR-T cells eliminate murine GB *in vivo*

To interrogate the anti-GB efficacy of the developed ligand-based constructs in the presence of a functional immune system, immunocompetent C57BL/6J mice were orthotopically implanted with 10^5 mCD70-expressing GL261 cells (GL261/CD70), and the presence of tumors was determined 14 days later by MRI ($N = 5$ mice per group). One day later, all mice received a single ICT dose of 2×10^6 mock-transduced or ligand-based mCAR-T cells, the CAR expression of which was verified earlier on the same day. Tumor size was monitored on a weekly basis by MRI (Figures 8A and 8B). When comparing pre- and post-therapy measurements, all mice treated with non-targeting murine T cells exhibited progressive tumor growth, whereas treatment with all ligand-based mCD70-targeting constructs resulted in significantly reduced tumor sizes, with the majority of animals achieving complete and durable remissions (Figures 8C and 8D). Across treatment arms, no differences in tumor size were observed prior to therapy; however, post-treatment, all mCD70-targeting constructs led to significantly smaller tumors compared to the control group (Figure 8E). This potent mCD70-driven cytotoxicity was translated to a significant OS prolongation. Whereas the median OS of mice treated with mock-transduced T cells was reported as 33 days, the median OS was notably not reached in any group receiving the ligand-based murine constructs, highlighting the robust and sustained antitumor efficacy of the mCD70-driven effector cell response (for mCAR27 vs. pMSCV, mCAR27/28 vs. pMSCV and mCAR27/BB vs. pMSCV: HR = 0.03725, 95% CI: 0.004861–0.2855, $p = 0.0015$; Figure 8F). Finally, by performing IF analysis on brains of these treated mice, we detected viable mCAR-T cells across all targeting constructs, an observation that coincided with a complete absence of murine GB cells. In contrast, sections from pMSCV-treated mice exhibited a dense network of tumor cells, along with a marked mCAR-T cell scarcity (Figure S9). In conclusion, our findings demonstrate that all mCD70-directed murine constructs can eliminate orthotopic GB in an immunocompetent setting, further supporting the translational potential of the ligand-based construct for clinical development.

DISCUSSION

In recent years, CD70 has emerged as a promising target antigen for CAR-T cell therapy in GB. Its role in promoting tumor growth,[4,7,8] maintaining GB stemness,[7] and inducing immunosuppression,[4,6–8] led to the inception of preclinical investigations targeting GB CD70 with CAR-T cells.[4,7,12] Despite being clinically relevant, a direct comparison of structurally different CD70-directed CAR constructs is still lacking. To the best of our knowledge, our study is the first comparative analysis of distinct CD70-directed CAR constructs in GB. While all constructs specifically eliminated $CD70^+$ GB cells, the CD27z design proved superior concerning cytokine secretion *in vitro* and prolonging OS *in vivo*. Similar results were reported by Sauer et al., demonstrating that CD70-directed CD27z CAR-T cells led to extended OS compared to the LFBBz and LF28z counterparts in an acute myeloid leukemia mouse model.[29] The observed difference might stem from the structural variation of the CARs that potentially impact the CAR-T cell phenotypic characteristics. CD27 is the natural ligand of CD70.[3] Therefore, incorporating a physiological ligand-receptor interaction in a CAR design may lead to optimal target binding, potentially reduced levels of tonic signaling, and eventually better outcomes compared to constructs consisting of multiple synthetic fusions. Future analyses will compare the CD27z CAR with counterparts utilizing only the extracellular domain of CD27, fused to the clinically relevant co-stimulatory domains of CD28 and/or CD137 (4-1BB). In our study, the LF28z construct eliminated GB cells the fastest *in vitro*. This agrees with the generally accepted concept that CAR-T cells with the CD28 co-stimulatory domain kill tumor cells more rapidly.[13,14,36] Relevantly, PD1/LAG3/TIM3 triple-positive cells in LF28z CAR-T cells were reported the lowest among all constructs after co-culture with CD70-expressing GB cells (Figure S3A), and the observed T_{EMRA} cell fraction, a differentiation state linked to higher cytotoxicity,[37] was higher in LF28z CAR-T cells (Figure 2F), potentially explaining the faster killing dynamics. However, it is also linked to lower persistence and proliferative potential,[37] which might explain the inferior OS of mice treated with LF28z CAR-T cells compared to the CD27z and LFBBz constructs. Conversely, the fraction of T_{SCM} cells was increased in CD27z cells, possibly explaining the superiority of this construct *in vivo*, as T_{SCM} cells persist longer and possess stronger self-renewal capabilities.[38,39] Future investigations will also focus on deciphering how the utilized co-stimulatory domains affect CAR-T cell metabolism. Previous studies indicate that CAR-T cells incorporating a CD28 co-stimulatory domain typically display elevated aerobic glycolysis, while constructs with a 4-1BB domain tend to promote increased mitochondrial biogenesis and oxidative phosphorylation.[13–15] Therefore, metabolic characterization of the present study's constructs would assist defining how the utilized co-stimulatory domains modulate the interplay between cytokine-driven activation and target-cell toxicity.

Figure 7. mCD27-based anti-murine CD70 CAR-T cells are potent against murine GB *in vitro*

(A) Murine CD27-based construct design. (B) Transduction efficiency of primary murine T cells by flow cytometry. A non-transduced (NT) sample from each donor mouse was used to determine gating. Data gated on single live $mCD3^+$ cells. (C) Measurement of *mCD70* gene expression levels in generated OE models by RT-qPCR. $N = 3$ technical replicates per cell line. (D) Measurement of mCD70 on the surface of generated murine OE GB models by flow cytometry (blue histograms). Signal was compared to that of an isotype control (red histograms).

(E) Quantification of secreted TNF-α in the SN of mGB/mCAR-T cell co-cultures by ELISA. $N = 3$ biological replicates per group.

(F) Pairwise comparisons of secreted TNF-α levels from (E) among targeting constructs. A one-way ANOVA with a post hoc Holm-Šídák test was used for significance. (G) Schematic representation of the live-cell imaging pipeline. (H) Quantification of tumor cell signal from (G) over time. A one-way ANOVA with a Dunnett's multiple comparisons test was used with data from the $t = 660$ min mark. For (C) and (E), an unpaired two-tailed t test was used for significance. Data are presented as mean (SD). $^*p < 0.05$, $^{**}p < 0.01$, $^{***}p < 0.001$, $^{****}p < 0.0001$; n.s., not significant; n.d., not detected.

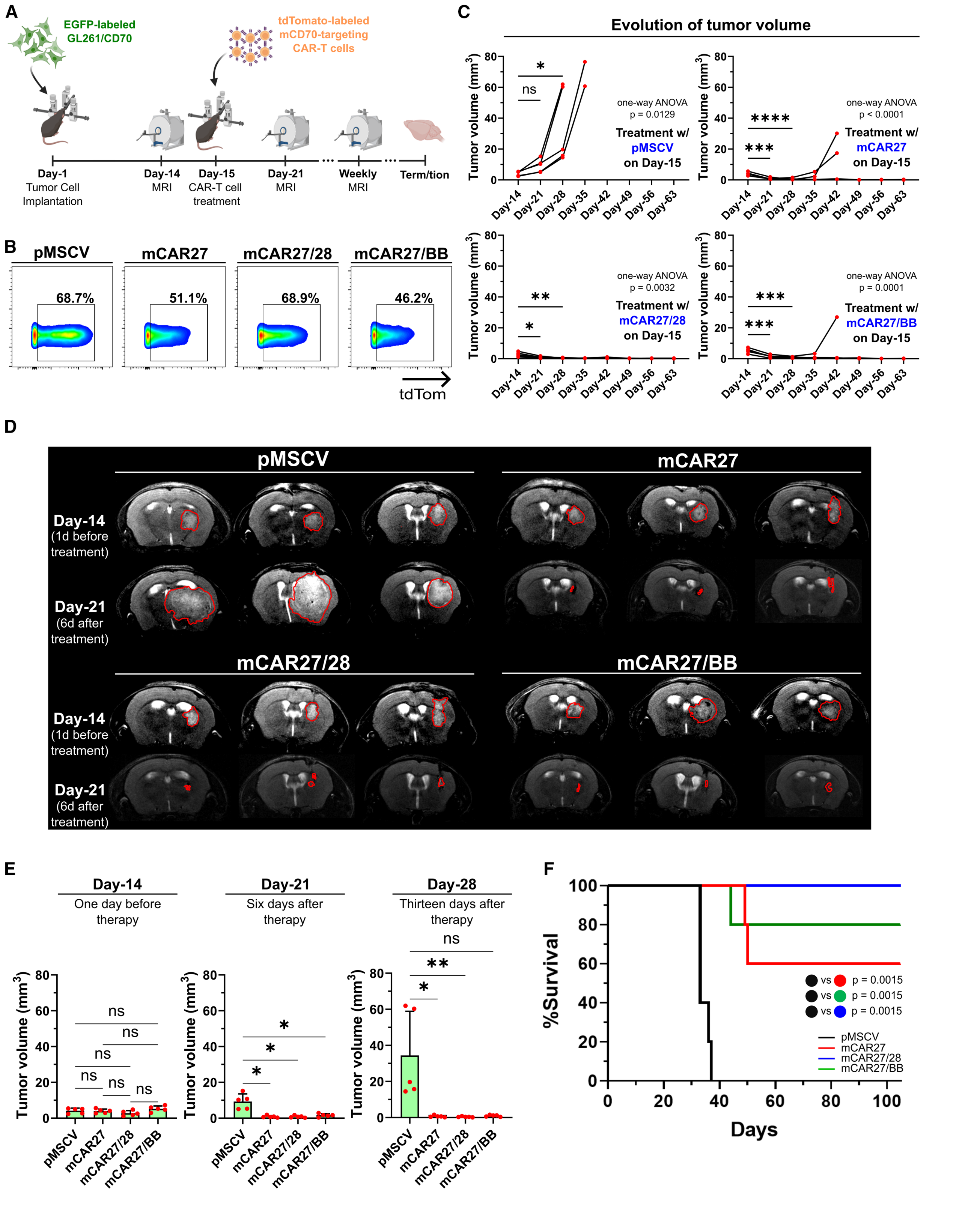

(legend on next page)

We also demonstrate the efficacy of CD70-directed CAR-T cells to eliminate primary GB cells in cerebral organoids. Despite the underlined success, these organoids are derived *in vitro* from human iPSCs via SMAD inhibition, therefore lacking mesodermal-stemming immune cells and microglia.[34,35] Relevantly, microglia constitutes a significant portion of brain tumors and contributes to tumor progression by chemokine and cytokine secretion.[40,41] Therefore, investigation of its interplay with CD70-directed CAR-T cells would be meaningful. Employment of patient-derived organoids can address this issue. Jacob et al. described a pipeline to develop GB-organoids from surgically resected GB tissue, preserving the molecular and histological features of parental tumors such as tumor heterogeneity, transcriptomic signature and mutational load. Importantly, their suitability as a platform to assess CAR-T cell therapies was elicited.[42,43] Utilization of such a model would benefit preclinical CAR-T cell investigations for GB treatment as it would allow to study the interplay between therapeutic and endogenous immune cells.

Except for one, all mice treated with human CD70-targeting CAR-T cells eventually experienced tumor recurrence, with reported CAR-T cell scarcity in recurrent lesions. This suboptimal long-term CAR-T cell persistence could be a result of exhaustion, as engagement of GB CD70 by all constructs led to a significant reduction of PD1/LAG3/TIM3 triple-negative effector cells compared to the control group (Figure S3B). This, combined with the prolonged antigen stimulation of the *in vivo* experiment might have led to exhaustion,[44] explaining tumor recurrence. In future studies, CAR-T cells will be isolated from treated mice at earlier post-treatment time points to enable assessment of exhaustion-associated marker expression. This approach will provide deeper insights into the temporal dynamics of our therapeutic intervention and inform the design of combination strategies to enhance persistence and prevent tumor relapse. Importantly, our analysis on recurrent tissue also revealed high and universal CD70 expression, restricted to GB-cells. This finding excludes antigen loss as a tumor recurrence mechanism in our study and provides rationale for a second local CAR-T cell administration as an approach to tackle recurrent disease, an intervention which is followed in the clinic and is well-tolerated by patients.[1,45]

Not only restricted to the immunocompromised setting, mCAR-T cells integrating mCD27 as target recognition and co-stimulatory domain demonstrated remarkable potency against murine GB cells *in vitro* and *in vivo*, with the median OS not reached by the end of the legally approved experimental period in any of the treatment groups. This robust and sustained tumor remission is likely attributable to the observed long-term mCAR-T cell persistence within the brains of treated mice, a fact which suggests communication of the administered mCAR-T cells with the endogenous immune system, reported by others.[46] Specifically, exogenously administered mCAR-T cells targeting orthotopically implanted murine GB tumors have been shown to induce a proinflammatory phenotype by driving an increase in T cell and dendritic cell infiltration, a decrease in the fraction of myeloid-derived suppressor cells, as well as enhancement of the antigen-presenting capacity of myeloid cells and reduction of the immunosuppressive regulatory T cell abundancy.[47,48] Future studies will determine whether these events contribute to the durable anti-GB activity observed with mCAR-T cells in our study. In conclusion, this comparative study emphasizes the efficacy of several CD70-directed CAR-T cell constructs against GB *in vitro* and *in vivo*, with the ligand-based one exhibiting the most prominent anti-glioma activity *in vivo*. Based on these results, a clinical trial evaluating the safety and efficacy of CD27z CAR-T cells for the treatment of patients with recurrent GB is currently in preparation.

Limitations of the study

One limitation of our study is the interpatient and intrapatient tumor heterogeneity regarding the qualitative and quantitative nature of CD70 expression. This represents a hallmark of GB and a phenomenon reported in clinical trials utilizing CAR-T cells for the treatment of GB. Consequently, it may hinder the success of cell therapy by rendering patients ineligible for treatment due to low or no expression of the target of interest, or by intercepting only part of the tumor. Additionally, although we have successfully demonstrated the very potent and specific CD70-driven CAR-T cell cytotoxicity in immunocompromised (NSG) and immunocompetent (C57BL/6J) mouse models, further incorporating a fully-humanized mouse model would allow us to provide deeper insights into the mechanics of tumor cell interception as well as to examine the interplay between administered human CAR-T cells and endogenous human immune cells. Finally, our study does not involve a thorough evaluation of our immunotherapy's safety profile. However, in our experience, on-target/off-tumor associated toxicities are not expected, since we perform local CAR-T cell administration in the brain, which is an organ that completely lacks CD70 expression, providing a rationale in favour of the safety of our administration method and our approach in general.

Figure 8. mCD27-based CAR constructs lead to regression of syngeneic GB tumors *in vivo*

(A) Schematic representation of the syngeneic model *in vivo* experiment. (B) Evaluation of mCAR expression on transduced murine T cells on treatment day by flow cytometry. A non-transduced sample was used to determine gating. Data gated on single live $mCD3^+$ cells. (C) Weekly tumor volume evaluation in C57BL/6J mice orthotopically implanted with GL261/CD70 cells and subsequently treated ICT with anti-mCD70 CAR-T cells by MRI. A one-way ANOVA with a Dunnett's multiple comparisons test was used to assess significance. (D) Indicative T2-weighed MRI sections (coronal plane) from $N = 3$ mice of each treatment group from (C), before and after treatment with anti-mCD70 CAR-T cells. (E) Comparison of tumor volumes among treatment groups from (C). For day-14, a one-way ANOVA followed by a Tukey's multiple comparisons test was used. For day-21, a Welch's ANOVA followed by a Dunnett's T3 multiple comparisons test was used. For day-28, a Kruskal-Wallis test followed by a Dunn's multiple comparisons test was used. Data presented as mean (SD).

(F) Overall survival of treated mice from (C). The log rank (Mantel-Cox) test was used to assess statistical significance. Bonferroni-adjusted significance threshold: $p = 0.0166$. *$p < 0.05$, **$p < 0.01$, ***$p < 0.001$, ****$p < 0.0001$; n.s., not significant; n.d., not detected.

MATERIALS AND METHODS

Cell culture

The origins of primary human GB cell lines T269, T325, S24, U3017, U3021, U3047, P3, BG5, and BG7 have been described in detail previously.[49] Primary human GB cell line MMKI was provided by Bryan Day.[30–32] Primary GB cells were maintained under neurosphere-forming conditions using DMEM-F12 (#11330-032, Gibco), supplemented with B27 (#17504044, Gibco), 5 μg/mL insulin (#I9278, Sigma-Aldrich), 5 μg/mL heparin (#H4784, Sigma-Aldrich), 20 ng/mL epidermal growth factor (EGF; #PHG0311, Gibco), and 20 ng/mL fibroblast growth factor (FGF; #PHG0021, Gibco), collectively referred to as "*human Glioma Cell Medium*" (hGCM).[50] U138 cells were purchased from the American Type Culture Collection (ATCC) and cultured in Dulbecco's modified eagle's medium (DMEM; #D5796, Sigma-Aldrich), supplemented with 10% (v/v) fetal bovine serum (FBS; #S0615, Sigma-Aldrich) and 1% (v/v) penicillin/streptomycin (#PS-B, Capricorn Scientific). Murine glioma cell lines GL261 and CT2A were purchased from ATCC and cultured in DMEM (#6429, Sigma-Aldrich), supplemented with 10% (v/v) FBS, referred to as "*murine Glioma Cell Medium*" (mGCM). HEK293 cells, obtained from ATCC, were cultured in Iscove's modified Dulbecco's medium (IMDM; #12440053, Gibco), supplemented with 10% (v/v) FBS. Cell line authenticity was confirmed by Multiplexion GmbH (Heidelberg, Germany), and cells were tested for mycoplasma contamination. Adherent lines U138, GL261, CT2A, and HEK293 were dissociated with Trypsin-EDTA (0.25%; #25200056, Gibco), while StemPro Accutase (#A1110501, Gibco) was used for primary cells.

Generation of OE and knockout models

For *CD70* OE in primary human GB cell lines S24, S24/EGFP, P3, P3/EGFP, T269 and T269/EGFP, the generation of the lentiviral construct and its non-expressing control equivalent are described in detail in our previous published work.[49] The exact same approach was followed for cloning the *mCD70* OE construct, with full-length *mCD70* (GenBank: NM_011617.2) serving as the integrated DNA fragment. The following murine cell lines were transduced with the *mCD70* OE or control constructs: GL261, GL261/EGFP, and CT2A. For human *CD70* gene knockout (KO), a single-guide RNA (5′-GTGCATCCAGCGCTTCGCAC-3′, U138/CD70_KO resulting cell line) or a control non-targeting sequence (5′-GTAGGCGCGCC GCTCTCTAC-3′, U138/Ctrl resulting cell line) were designed with the CHOP/CHOP online design tool (RRID:SCR-015723) and synthesized by Eurofins Genomics. Complementary oligonucleotide sequences were annealed and phosphorylated with T4 Polynucleotide Kinase (#M0201S, New England Biolabs). Then, the oligonucleotides were inserted into the LentiCRISPRv2GFP vector by digestion with BsmbI-v2 (#R0739S, New England Biolabs) and ligation using T4 DNA ligase (#M0202S, New England Biolabs). Ligation products were then used to transform DH5α competent bacterial cells (#18265017, Invitrogen). Single bacterial clones were selected and cultured O/N in Luria-Broth medium (#A0954, PanReac AppliChem) with 100 μg/mL ampicillin (#A5354, Sigma-Aldrich). Plasmid DNA was purified with the QIAprep Spin Miniprep Kit (#27106, Qiagen). To produce lentivirus for transduction of human and murine cell lines, HEK293 cells were co-transfected with 4.7×10^{11} copies of transfer plasmid and packaging plasmids psPAX2 (Addgene plasmid #12260; http://n2t.net/addgene:12260; RRID:Addgene_12260, gift from Didier Trono) and pMD2.G (Addgene plasmid #12259; http://n2t.net/addgene:12259; RRID:Addgene_12259, gift from Didier Trono) using TransIT-LT1 Transfection Reagent (#MIR 2304, Mirus Bio). Supernatant was collected 24 and 48 h after transfection, filtered, and lentiviral particles were concentrated using the PEG-it Virus Precipitation Solution (#LV810A-1, Systems Bioscience). Human and murine cells were transduced in the presence of 8 μg/mL polybrene (#TR-1003-G, Sigma Aldrich). Growth medium was exchanged 24 h later. Transduced human and murine cells were sorted on a BD FACSAria fusion instrument (BD Biosciences) based on CD70 expression. For cloning of the lentiviral human and murine CD70 OE construct, the pLEX306 was a gift from David Root (Addgene plasmid #41391; http://n2t.net/addgene:41391; RRID:Addgene_41391). For cloning of the lentiviral CD70 knockout construct, the LentiCRISPRv2GFP was a gift from David Feldser (Addgene #82416; http://n2t.net/addgene:82416; RRID:Addgene_82416).

CAR design

For human CD70, retroviral CAR constructs CD27z, LF28z, and LFBBz have been described previously.[29] To introduce the IRES/tdTomato cassette into the human-CD70 targeting retroviral CAR constructs, the IRES2/tdTomato region of the LeGO-iT vector was amplified with primers featuring SphI/BspEI (forward primer 5′-GTGACAGCATGCATTCCTGCAGGCCTCGACGA-3′ and reverse primer 5′-TGTCACTCCGGATCGACGAATTTCGACCA CTGTGC-3′) or NotI/MluI (forward primer 5′-GTGACAGCGG CCGCATTCCTGCAGGCCTCGACGA-3′ and reverse primer 5′-TGTCACACGCGTTCGACGAATTTCGACCACTGTGC-3′) overhangs for cloning into the CD27z or the LF28z/LFBBz constructs, respectively. PCR products were purified with the DNA Clean & Concentrator-5 kit (#D4003, Zymo Research) and cloned into the CD27z vector after double digestion with SphI (#R3182S, New England Biolabs) and BspEI (#R0540S, New England Biolabs), using T4 DNA Ligase (# New England Biolabs, M0202S). For cloning into the LF28z/LFBBz vector, the respective PCR products and destination backbones underwent double digestion with MluI (#R3198S, New England Biolabs) and NotI (#R3189S, New England Biolabs). A non-targeting control construct encoding only tdTomato was also generated (referred to as SFG). To generate it, the tdTomato region of the LeGO-iT vector was amplified with primers featuring XhoI/MluI overhangs (forward primer 5′-GTGACACTCGAGCGATG GTGAGCAAGGGCGAG-3′ and reverse primer 5′- CAAGCTAC GCGTTTACTTGTACAGCTCGTCCATGCC-3′), purified as above, and cloned into the LFBBz construct after dual digestion with XhoI (#R0146S, New England Biolabs) and MluI. DH5α competent bacterial cell transformation, O/N culture, picking and growing of single clones and DNA isolation were performed as above. LeGO-iT was a gift from Boris Fehse (Addgene plasmid #27361;

http://n2t.net/addgene:27361; RRID:Addgene_27361). For murine CD70, retroviral constructs mCAR27, mCAR27/28, and mCAR27/BB were designed in-house and synthetized by VectorBuilder. The full-length sequence of mCD27 was fused either to the cytoplasmic portion of the mCD3z chain (mCAR27), or the cytoplasmic domain of mCD28 and then that of mCD3z (mCAR27/28), or the cytoplasmic domain of mCD137 (m4-1BB) and then that of mCD3z (mCAR27/BB). The tdTomato cassette was fused to the above sequences, driven by a T2A self-cleavage site. Like with the human counterparts, a non-targeting control construct encoding only tdTomato was also generated (referred to as pMSCV).

Retrovirus production and T cell transduction

Human T cells

HEK293 cells were co-transfected using 3.75 μg of MLV gag/pol-expressing plasmid pEQ-Pam3(-E), 2.5 μg of envelope-encoding plasmid pLTR-RD114A,[29] and 3.75 μg of SFG, CD27z, LF28z, or LFBBz constructs (all tdTomato-encoding) using GeneJuice (#70967, Sigma-Aldrich). Supernatant (SN) was collected 48 h post-transfection, filtered, and used to transduce activated human T cells from healthy donors, which were isolated from Buffy Coats as described previously,[49] and activated for 48 h in medium consisting of 45% (v/v) Click's medium (#C5572, Sigma-Aldrich), 45% (v/v) RPMI-1640 (#11875093, Gibco), 10% (v/v) FBS, 10 ng/mL IL-7 (#200-07, Peprotech), and 5 ng/mL IL-15 (#200-15, Peprotech, hereby referred to as "*hCAR medium*"), further supplemented with a 1:100 dilution of human T cell TransAct (#130-111-160, Miltenyi Biotec), at a density of 10^6 cells/mL. Human T cell retroviral transduction was performed as previously described.[49] Transduction efficiency was evaluated 96 h later by flow cytometry.

Murine T cells

HEK293 cells were co-transfected with 4.75 μg of gag/pol/env-encoding plasmid pCL-Eco (Addgene #12371; http://n2t.net/addgene:12371; RRID:Addgene_12371, gift from Inder Verma) and 3 μg of murine retroviral constructs pMSCV, mCAR27, mCAR27/28, and mCAR27/BB using GeneJuice (#70967, Sigma-Aldrich). SN was collected 48 h post-transfection, filtered, and used to transduce activated murine T cells. To isolate primary murine T cells, C57BL/6J mice (Janvier Labs) were sacrificed by cervical dislocation and the spleen and lymph nodes were removed using aseptic technique. They were meshed through a 70 μm strainer (#CNA0.1, Carl Roth) and washed twice with PBS. Meshed spleens were resuspended in ACK Lysing Buffer (#A1049201, Invitrogen) at RT for 1.5 min, washed with PBS, and meshed again. The spleen and LN preparation products were pooled, and murine T cells were enriched using the MagniSort Mouse T cell Enrichment Kit (#8804-6820-74, Invitrogen). Isolated T cells were activated on 24-well plates, previously coated with 20 μg/mL anti-hamster IgG whole molecule (#55397, MP Biomedicals) at 37°C for 4 h, followed by a PBS wash and coating with 4 μg/mL Ultra-LEAF Purified anti-mouse CD3 Antibody (#100340, Biolegend, RRID: AB_11149115) at 4°C for 1 h. Activation took place in T cell proliferation medium (TCPM), supplemented with 5 μg/mL Ultra-LEAF Purified anti-mouse CD28 Antibody (#102115, Biolegend, RRID: AB_11150408) and 100 IU/mL recombinant human IL-2 (#130-097-743, Miltenyi Biotec). TCPM comprised RPMI-1640 supplemented with 10% (v/v) FBS, 1% (v/v) penicillin/streptomycin, 2 mM L-Glutamine (#25030081, Gibco), 50 μM β-mercaptoethanol (#M6250, Sigma-Aldrich), 25 mM HEPES (#15630080, Gibco), 1 mM sodium pyruvate (#11360070, Gibco), and 0.1 mM MEM non-essential amino acids solution (#11140050, Gibco). Murine T cell transduction took place 36 h after activation, exactly as described for primary human T cells.[49] Transduced murine T cells were expanded in TCPM supplemented with 100 IU/mL IL-2, referred to as "*mCAR-medium.*" Transduction efficiency was evaluated 96 h later by flow cytometry.

qPCR

The RNeasy Mini Kit (#74104, Qiagen) was used to extract RNA from 10^6 GB cells. The MultiScribe Reverse Transcriptase kit (#4311235, Invitrogen) was employed to reverse-transcribe 1 μg RNA. Gene expression quantification was achieved on a QuantStudio-3 real-time PCR system (Applied Biosystems) using TaqMan Gene Expression Master Mix (#4369016, Applied Biosystems) and TaqMan probes targeting human *CD70* (assay ID: Hs00174297_m1, Applied Biosystems) and human *GAPDH* (assay ID: Hs99999905_m1, Applied Biosystems), or mouse *CD70* (assay ID: Mm00441914_m1, Applied Biosystems) and mouse *GAPDH* (assay ID: Mm99999915_g1, Applied Biosystems).

Generation of luminescent tumor models

The lentiviral plasmid pCDH_NanoLuc/EGFP was a kind gift from Patricia Benites. The human primary GB cell line P3 was transduced to generate the P3/CD70_NLuc/EGFP cell line. The functionality of the NLuc gene was verified using the Nano-Glo Luciferase Assay System kit (#N1120, Promega) according to the manufacturer's instructions.

Generation of cerebral organoids

Human induced pluripotent stem cells (iPSC) were subjected to dual SMAD inhibition using SB-431542 (#SM33, Cell Guidance Systems), LDN193189 (#72149, Stem Cell Technologies), and XAV (#SM38-50, Cell Guidance Systems) to differentiate into neural precursor cells (NPC). Cells were cultured in DMEM/F12 supplemented with N2-supplement (#17502048, Gibco), B27-supplement, 1% (v/v), GlutaMax (#35050061, Gibco), 1% (v/v) NEAA (#11140035, Gibco), 1.6 mg/mL D-Glucose (#G8270, Sigma-Aldrich), 1% (v/v) penicillin/streptomycin, 1 μM LDN193189, 10 μM SB-431542, and 2 μM XAV. After 8 d, the concentration of LDN193189 was reduced to 200 nM and SB-431542 was omitted. Cells were dissociated into single-cell suspensions using TrypLE Express (#12604013, Gibco) and 10^5 cells were plated in U-bottom plates pre-coated with 0.5% (v/v) Pluronic (#435465, Sigma-Aldrich) in the same culture medium, supplemented with 50 μM Y-27632 (#SCM075, Sigma-Aldrich). 48 h later, the resulting spheroids were transferred to 6 cm dishes. After 5 d, the small molecules were replaced with 20 ng/mL EGF and 20 ng/mL FGF for 19 d. The culture medium was changed every 2 d, and EGF/FGF were

removed starting from day 27. Neuronal differentiation was subsequently initiated via Notch inhibition.

Co-culture

Evaluation of human CAR-T cell cytotoxicity by flow cytometry and ELISA

Human GB cells were dissociated as above, washed, and seeded on wells of a U-bottom 96-well pate (10^5 cells per well, #83.3925.500, Sarstedt) in hCAR medium. Immediately afterwards, effector cells were harvested, washed, and 2×10^5 CAR-T cells were added to the GB cells in hCAR medium without exogenous cytokines. After 18 h, SN was collected for ELISA and CAR-T cell activation was evaluated by flow cytometry.

Evaluation of murine CAR-T cell cytotoxicity by flow cytometry and ELISA

Murine GB cells were dissociated with trypsin and 1×10^5 cells were seeded on the wells of 96-well plate (#92196, TPP) in mGCM. On the next day, mGCM was removed and 2×10^5 mCAR-T cells were added to each well in TCPM without exogenous cytokines. SN was collected 18 h later for ELISA and mCAR-T cell activation was assessed by flow cytometry.

Evaluation of human CAR-T cell cytotoxicity on the Incucyte platform

Wells of a 96-well plate (#655090, Greiner Bio-One) were coated with a 1:50 dilution of growth factor-reduced Corning Matrigel Matrix (#356231, Corning) in hGCM for 1 h at 37°C. Afterward, matrigel was removed and 7.5×10^3 P3/GFP_MCS or P3/GFP_CD70 cells were seeded in hGCM medium supplemented with D-Glucose (50 mM final concentration). Adherent U138/CD70_KO and U138/Ctrl cells were directly seeded in DMEM, supplemented with 10% (v/v) FBS and 1% (v/v) penicillin/streptomycin. Next day, 7.5×10^3 effector cells were added per well in hCAR medium without cytokines. The Incucyte Annexin-V Near Infrared Dye (#4768, Sartorius) was added at a final concentration of 1:200. Live-cell imaging immediately commenced on the Incucyte SX5 instrument using the 10x objective. The time interval between two given consecutive timepoints was 45 min. Data from the SX5 Incucyte instrument were analyzed on the Incucyte Analysis Software (version 2020C, Sartorius) using the Incucyte Basic Analysis Module. The confluency mask was first set on the phase contrast channel to identify cells and background noise by defining cell size, eccentricity as well as background signal threshold values. Next, analysis parameters for the EGFP (tumor cell signal) and the NIR (Annexin-V incorporation to flipped phosphatidylserines) channels were defined. A Top-Hat segmentation approach was followed to correct for fluorescent background signal. Co-localization analysis was performed by generating an overlap mask from the previously defined parameters over time. The following metrics were calculated for every sample, image and time point: (a) Annexin-V$^+$/EGFP$^+$ object count divided by total EGFP$^+$ object count and (b) EGFP$^+$ total object count. Both generated metrics were normalized against the first measured time point.

Evaluation of mCAR-T cell cytotoxicity against mCD70-expressing GB cells by live cell imaging

Murine GB cells were dissociated, washed, and seeded on wells of a 96-well plate (10^5 cells per well, #655090, Greiner Bio-One) in mGCM. Next day, medium was removed, and 10^5 mCD70-targeting CAR-T cells were added in TCPM, without exogenous cytokines. The plate was spun down 500 G for 5 min and live cell confocal microscopy commenced immediately on a LSM980 confocal microscope (Carl Zeiss). Image acquisition and data analysis are described in detail in supplemental methods.

Evaluation of human CAR-T cell cytotoxicity in 3D cerebral organoids

Organoids were cultured in advanced MEM (#11095080, Gibco), enriched with B27 supplement, GlutaMax, 5 mg/mL D-glucose, and 1% (v/v) penicillin/streptomycin, a composition hereby referred to as "*Organoid medium.*" GB cells were dissociated and washed once with PBS. A total of 5×10^3 cells were then seeded in U-bottom 96-well plates, pre-coated with 0.5% (v/v) pluronic, in organoid medium, further supplemented with 20 ng/mL EGF and 20 ng/mL FGF. One organoid per well was then manually added. Six days later, EGF and FGF were removed from the medium. Fourteen days after the co-culture initiation, organoids were either harvested and fixed in 4% (v/v) paraformaldehyde (PFA; #sc-281692, Santa Cruz Biotechnology) in PBS for subsequent IF analysis or treated with CD70-targeting CAR-T cells. For treatment, CAR-T cells were harvested, washed once with PBS, and 25×10^3 cells were added per well in organoid medium. SN was collected prior to, and 2, 3, and 5 d following the addition of CAR-T cells. Organoids were fixed as above 3 d after CAR-T cell addition for downstream IF analysis.

Flow cytometry

For all flow cytometry analysis experiments, human cells were washed with PBS and blocked in human serum (#H4522, Sigma-Aldrich), diluted 1:10 in PBS for 10 min at RT. Murine cells were washed with PBS and blocked with a 10 μg/mL a-mCD16/a-mCD32 mix (#14-0161-82, Thermo Fisher Scientific) in PBS for 10 min at RT. After blocking, antibody incubation took place for 30 min at 4°C, protected from light. Cell viability was evaluated using the eBioscience Fixable Viability Dye eFluor 780 (#65-0865-14, eBioscience). Following incubation, cells underwent two washes with PBS. Data acquisition was carried out using an LSR Fortessa instrument (BD Biosciences), and data analysis was conducted with FlowJo (version 10.10.0, Treestar). For evaluation of human CD70 expression on GB cells, 10^6 GB cells cells were stained with anti-human CD70-PE (#555835, BD Biosciences, RRID: AB_396158). For evaluation of human T cell transduction efficiency and CAR-T cell phenotyping, before or after target cell stimulation transduced T cells were incubated with the following antibody mixes: (a) anti-human CD3-BV711 (#317328, Biolegend, RRID: AB_2562907), anti-human CD366-BV421 (#364808, Biolegend, RRID: AB_3068174), anti-human CD223-BV510 (#369318, Biolegend, RRID: AB_2715780), and anti-human CD279-APC (#379208, Biolegend, RRID: AB_2922606), (b) anti-human CD3-BV711 (#317328,

Biolegend, RRID: AB_2562907), anti-human CD4-PacificBlue (#344620, Biolegend, RRID: AB_2832384), and anti-human CD8a-APC (#300912, Biolegend, RRID: AB_314115), or (c) anti-human CD3-BV711 (#317328, Biolegend, RRID: AB_2562907), anti-human CD197-FITC (CCR7, #353216, Biolegend, RRID: AB_10924057), anti-human CD62L-PacificBlue (#304825, Biolegend, RRID: AB_2186977), anti-human CD45RA-APC (#304112, Biolegend, RRID: AB_2564158), and anti-human CD45RO-BV605 (#304238, Biolegend, RRID: AB_2562143). For assessment of CAR-T cell activation after co-culture with MMKI cells, the following antibodies were used: anti-human CD3-APC (#100236, Biolegend, RRID: AB_312660) and anti-human CD137-BV421 (#309820, Biolegend, RRID: AB_2563830). Regarding murine cells, for measurement of mCD70 levels on murine glioma cell lines, 10^6 cells were stained with anti-mouse CD70-PE (#104606, Biolegend, RRID: AB_2291343). For evaluation of transduction efficiency of murine T cells, the following mix was used: anti-mouse CD3-BV711 (#100241, Biolegend, RRID: AB_2563945), anti-mouse CD8a-APC (#100712, Biolegend, RRID: AB_312751), and anti-mouse CD4-FITC (#100406, Biolegend, RRID: AB_312691). For assessment of mCAR-T cell activation after co-culture with murine GB models, the following mix was used: anti-mouse CD3-BV711 (#100241, Biolegend, RRID: AB_2563945) and anti-mouse CD137-APC (#106110, Biolegend, RRID: AB_2564297). All isotype antibodies used in this study are provided in supplemental methods.

ELISA

The following kits were used to quantify cytokine secretion: Human Granzyme-B DuoSet enzyme-linked immunosorbent assay kit (ELISA; #DY2906–05, R&D Systems), Human IFN-gamma DuoSet ELISA kit (#DY285B, R&D Systems), Human TNF-alpha DuoSet ELISA kit (#DY210, R&D Systems), and Mouse TNF-alpha DuoSet ELISA kit (#DY410, R&D Systems).

Animal experiments

Animal housing and welfare measures are described in detail in supplemental methods. For orthotopic implantation of GB cells, 7–12-week-old male NOD.Cg-Prkdc^{SCID}Il2rg^{tm1Wjl}/SzJ (NSG) immunocompromised mice, or C57BL/6J immunocompetent mice, purchased from Janvier Labs, were anesthetized with an intraperitoneal (i.p.) injection of a 100 mg/kg body-weight ketamine (LOT# FS1670041, belapharm) and 20 mg/kg body weight xylazine (LOT# PZN-01320422, Elanco) mixture. Tumor cells were loaded on a 10 μL Hamilton syringe (#80308, Hamilton) and 10^5 (for NSG mice) or 5×10^4 (for C57BL/6J mice) cells were stereotactically injected into the right hemisphere, 2 mm right lateral of the bregma and 1 mm anterior to the coronal suture, 3 mm below the dural surface, with an injection rate of 5×10^4 cells/min. On treatment day, human or murine CAR-T cells were harvested and washed with PBS. Intratumoral (ICT) administration of 2×10^6 therapeutic or control cells took place as described above, at a rate of 10^6 cells/min. Upon meeting termination criteria, animals were intracardially perfused with PBS followed by 4% (w/v) PFA in PBS, under deep anesthesia, and brains were subsequently fixed and frozen as previously described.[49]

Immunofluorescence

On cerebral organoids

Fixation took place in 4% (w/v) PFA in PBS at RT for 20 min and organoids were then transferred to 30% (w/v) sucrose in PBS O/N at 4°C. Embedding took place in a 10% (w/v) gelatin (#9000-70-8, Sigma-Aldrich)/7.5% (w/v) sucrose solution in PBS. Organoids were frozen at −80°C. Cryosectioning was performed to generate sections of 20 μm thickness. Sections were blocked in 10% (v/v) horse serum (#16050130, Gibco) in PBS, supplemented with 0.1% (v/v) Triton-X at RT for 1 h. Samples were then incubated O/N at 4°C with the following primary antibody mixes, diluted 1:1000 in blocking buffer: (a) chicken anti-EGFP (#GFP1020, Aves Labs) and mouse anti-human CD70 (#ab77868, Abcam) or (b) chicken anti-EGFP, rabbit anti-RFP (#600-401-379, Rockland) and mouse anti-Granzyme-B (#MA1-80734, Invitrogen). Samples were washed three times in TBS-T and incubated at RT for 1 h in the dark with the following secondary antibodies diluted in TBS-T, used in different combinations: Donkey anti-Mouse IgG (H + L) Alexa Fluor 647, Goat anti-Chicken IgY (H + L) Alexa Fluor 488, and Donkey anti-Rabbit IgG (H + L) Alexa Fluor 546. Samples were then washed three times in TBS-T, counterstained with DAPI as above, and washed three times in TBS-T. Mouse brain tissue and organoid sections were mounted on Superfrost slides (#J1800AMNZ, Thermo Fisher Scientific) using the Fluoromount Aqueous Mounting Medium (#F4680, Sigma-Aldrich). Slides were allowed to dry at 4°C for 1 h and were stored at 4°C until confocal microscopy. Confocal microscopy was performed on the LSM980 instrument (Zeiss). The following lasers were used: 5 mW 405 nm diode (detection of DAPI), 10 mW 488 nm diode (detection of Alexa Fluor 488 signal), 10 mW 561 nm diode (detection of Alexa Fluor 546 signal), and the 5 mW 640 nm diode (detection of Alexa Fluor 647 signal). The laser power, digital offset, and digital gain were adjusted for each individual experiment and remained constant across all conditions. The 20x/0.8 Plan-Apochromat M27 (a = 0.55 mm) and 40x/1.2 LD LCI Plan-Apochromat DIC M27 (a = 0.40 mm, D = 0.17 mm) objectives were used. All images were acquired with a bit depth of 16 and by generating z-stacks. Acquired images were processed using Fiji software (version 1.53c, National Institutes of Health). Channels were first split and maximum intensity projections from all obtained z-stacks were generated. The same brightness and contrast adjustments were then applied to all samples. For quantification of CD70 and Granzyme-B signal on cerebral organoids, the corresponding channels were converted to a binary format using the same thresholding method across all samples and conditions, with a dark background setting. The area of each individual organoid was then calculated using the DAPI channel. For each sample, the % fraction of the total organoid area occupied by CD70 or Granzyme-B signal was then calculated.

On treated mouse brain tissue

IF was performed on 50 μm free-floating cryosections. Sections were placed into a 24-well plate and subjected to two washes in Tris-buffered saline (TBS), followed by a wash in TBS containing 0.4% (v/v) Triton-X (TBS-T; #648466, Sigma Aldrich). All washes, unless

otherwise specified, were carried out at RT for 15 min using 500 μL per well. Sections were blocked for 90 min in TBS-T supplemented with 0.25% (w/v) bovine serum albumin (BSA; #T844.2, Roth) and 10% (v/v) normal goat serum (#5425, Cell Signaling Technology) at RT. Next, sections were incubated O/N at 4°C with the following primary antibodies diluted 1:1000 in TBS-T: (a) chicken anti-EGFP (#GFP1020, Aves Labs), rabbit anti-RFP (#600-401-379, Rockland), and mouse anti-human CD70 (#ab77868, Abcam) for staining of treated NSG mouse brains and (b) chicken anti-EGFP (#GFP1020, Aves Labs) and rabbit anti-RFP (#600-401-379, Rockland) for staining of treated C57BL/6J mouse brains. On the following day, after four washes in TBS-T, sections were exposed to secondary antibodies O/N at 4°C, in the dark, while gently rotating. The following secondary antibodies were used in different combinations: Goat anti-chicken IgY (H + L) Alexa Fluor 488 (#A32931, Invitrogen; 1:500), donkey anti-rabbit IgG (H + L) Alexa Fluor 546 (#A10040, Invitrogen; 1:500), and donkey anti-mouse IgG (H + L) Alexa Fluor 647 (#A31571, Invitrogen; 1:500), diluted in TBS. Sections were then washed twice with TBS-T, followed by one wash in TBS. All samples were counterstained with 1 μg/mL DAPI (#62248, Invitrogen) in TBS for 15 min at RT, followed by three washes in TBS-T and one in TBS. Sections were mounted as above. Slides were allowed to dry at 4°C for 1 h and were stored at 4°C. Confocal microscopy was performed exactly as described above, utilizing the same instrument setup.

Immunohistochemistry

IHC was performed on 1 μm FFPE tissue sections using the BenchMark Ultra immunostainer (Ventana Medical Systems). Samples were stained with a rabbit anti-CD70 antibody (#A700-266, Fortis Life Science; 1:100). Sections were mounted on StarFrost Advanced Adhesive slides (#11270, Engelbrecht) and dried for 15 min at 80°C. Slides were pretreated with Cell Conditioning Solution CC1 (#05279801001, Ventana Medical Systems) for 32 min at RT. Primary antibody incubation occurred for 32 min at 37°C, followed by Ventana's standard signal amplification kit (#05266114001, Ventana Medical Systems), UltraWash, counterstaining with hematoxylin (one drop for 4 min), and bluing reagent (one drop for 4 min). The chromogenic reaction was performed using the UltraView Universal DAB Detection Kit (#05269806001, Ventana Medical Systems). Stained slides were scanned with the Aperio AT2 Scanner (Aperio Technologies, Vista, USA) and digitized via Aperio ImageScope software (version 12.3.2.8013, Leica Biosystems).

MRI

For MRI, mice were anesthetized with 2% (v/v) isoflurane for induction, and anesthesia was maintained at a reduced concentration of 1–1.5% (v/v) isoflurane. Throughout the process, animals were positioned on a heating pad to preserve stable body temperature, and their breathing was continuously monitored using a respiratory sensor pad linked to a LabVIEW-based system developed in-house (National Instruments Corporation). MRI scans were conducted using a 9.4 T horizontal bore small animal MR scanner (BioSpec 94/20 USR, Bruker BioSpin), equipped with a four-channel phased-array surface coil. To assess tumor volume, a 3D T2-weighted rapid acquisition sequence with refocused echoes (RARE) was employed. The sequence parameters were set as follows: echo time (TE) = 72.56 ms, repetition time (TR) = 1800 ms, flip angle = 90°, matrix size: 200 × 100 × 120, with one average, slice thickness of 100 μm, and an isotropic resolution of 100 μm. The total acquisition time was 3 min. Post-acquisition, MR images were saved as DICOM files and visualized using Slicer imaging software (version 4.11.20210226).

In vivo bioluminescence imaging

In vivo imaging of brain tumors was performed on the IVIS Lumina III system (PerkinElmer). Animals received an i.p. injection of furimazine (#N1120, Promega) at a dose of 250 μg/kg body weight, diluted in PBS. Imaging commenced 10 min after injection with 10 min exposure time under isoflurane-induced inhalation anesthesia. Data were analyzed using the Living Image Software (version 4.7.2, PerkinElmer). Brightness was set to 100, contrast was set to 1.5, and opacity was set to 100. Binning was set to 1, and the color scale ranged from 1×10^4 to 3×10^5. For every animal, regions-of-interest of the same size were drawn around the tumor area, and the average radiance values (p/sec/cm^2/sr) were calculated and compared.

Statistical analysis

Data were analyzed with GraphPad Prism (version 10.1.2; GraphPad Software). Results are expressed as mean (SD) unless otherwise stated. The applied statistical tests are always mentioned in the corresponding figure captions. Data normality was investigated. Data were visualized using GraphPad Prism (version 10.1.2; GraphPad Software). Illustrations were made using BioRender (BioRender Inc.). Figures were assembled using Inkscape (version 1.3.2, Inkscape Developers).

Study approvals

All animal experiments were approved by the local regulatory authority ("*Regierungspräsidium Karlsruhe*," Karlsruhe, Germany, ref. nr. G-51/22) and performed following the institutional laboratory animal research guidelines. Utilization of mice that served as murine T cell donors was approved both institutionally (ref. nr. DKFZ-401) and by the local regulatory authority ("*Regierungspräsidium Karlsruhe*," Karlsruhe, Germany, ref. nr. G-51/22). All genetic engineering work in this study has been approved by the local regulatory authority ("*Regierungspräsidium Tübingen*," Tübingen, Germany, ref. nr. DKFZ.HD.04.03-39).

DATA AND CODE AVAILABILITY

Data and protocols are provided in the main article or as supplemental material and are available from the corresponding author upon reasonable request.

ACKNOWLEDGMENTS

We would like to thank Bryan Day, Brett Stringer, Dieter Lemke, Hrvoje Miletic, and Tobias Bergström for providing primary GB cell lines. Our gratitude is also extended to Damir Krunic, Manuela Brom, and Felix Bestvater (DKFZ Light Microscopy Core Facility), but also to Steffen Schmitt and Florian Blum (DKFZ Flow Cytometry Core

Facility) for providing the instruments for the experiments but also for their expert input. Finally, our gratitude is extended to Daniel Dominguez Azorin for assistance with confocal microscopy. The work was supported by the SFB grant UNITE Glioblastoma (SFB1389, WP A03 to W.W. and T.K.) of the German Research foundation (DFG) and the European Center for Neurooncology, funded by the Dietmar Hopp-Foundation. T.K. and A.K.S. were supported by a Hertie Network of excellence in clinical neuroscience fellowship by the Hertie Foundation. A.K.S. is funded by the Emmy-Noether Program by the DFG (project ID SU 1548/1-1). P.K. was funded by the Hector Stiftung II and by the *Bundesministerium für Bildung und Forschung* (BMBF; GBM3DTest, FKZ: 16LW0500K to P.K.).

AUTHOR CONTRIBUTIONS

A.K. designed the study and performed the following experiments and downstream data analysis and interpretation: qPCR, generation of overexpression and knockout models, cloning of CAR and knock-out constructs, all flow cytometry-related experiments, *in vitro* co-culture, confocal microscopy, design and execution of cerebral organoid experiments, ELISA, tumor and CAR-T cell implantation and bioluminescence imaging; E.B. contributed to co-culture and ELISA experiments; H.N.H.C. and D.A.A. assisted with surgical procedures on animals; R.Wi. generated the *CD70* overexpression models; P.K., S.H., and A.J. assisted and executed cerebral organoid experiments; D.R. and S.P. performed tissue sectioning and immunofluorescence; M.B., M.F., and R.S. assisted with MRI measurements; Y.-C.C. analyzed and provided single cell data; D.C.F.H., L.H., and M.H. analyzed and provided bulk RNAseq data; A.K.S., F.S., and A.v.D. stained glioblastoma patient samples; H.R. performed cell-culture and assisted with ICT administration of therapeutic CAR-T cells; C.S. assisted with experiments on the Incucyte platform and analyzed the data; M.P., L.B., L.K., and R.Wa. interpreted data and provided experimental input; T.S., C.M.T., and M.S. provided input on experimental design and provided material for CAR-T cell generation; W.W., T.K., and T.S. supervised the study and interpreted data throughout. The present manuscript was written by A.K. and approved by all listed co-authors prior to submission.

DECLARATION OF INTERESTS

The authors declare no competing interests.

SUPPLEMENTAL INFORMATION

Supplemental information can be found online at https://doi.org/10.1016/j.omton.2026.201134.

REFERENCES

1. Brown, C.E., Hibbard, J.C., Alizadeh, D., Blanchard, M.S., Natri, H.M., Wang, D., Ostberg, J.R., Aguilar, B., Wagner, J.R., Paul, J.A., et al. (2024). Locoregional delivery of IL-13Rα2-targeting CAR-T cells in recurrent high-grade glioma: a phase 1 trial. Nat. Med. *30*, 1001–1012. https://doi.org/10.1038/s41591-024-02875-1.
2. Choi, B.D., Gerstner, E.R., Frigault, M.J., Leick, M.B., Mount, C.W., Balaj, L., Nikiforow, S., Carter, B.S., Curry, W.T., Gallagher, K., and Maus, M.V. (2024). Intraventricular CARv3-TEAM-E T Cells in Recurrent Glioblastoma. N. Engl. J. Med. *390*, 1290–1298. https://doi.org/10.1056/NEJMoa2314390.
3. Goodwin, R.G., Alderson, M.R., Smith, C.A., Armitage, R.J., VandenBos, T., Jerzy, R., Tough, T.W., Schoenborn, M.A., Davis-Smith, T., Hennen, K., et al. (1993). Molecular and biological characterization of a ligand for CD27 defines a new family of cytokines with homology to tumor necrosis factor. Cell *73*, 447–456. https://doi.org/10.1016/0092-8674(93)90133-B.
4. Jin, L., Ge, H., Long, Y., Yang, C., Chang, Y.E., Mu, L., Sayour, E.J., De Leon, G., Wang, Q.J., Yang, J.C., et al. (2018). CD70, a novel target of CAR T-cell therapy for gliomas. Neuro Oncol. *20*, 55–65. https://doi.org/10.1093/neuonc/nox116.
5. Verhaak, R.G.W., Hoadley, K.A., Purdom, E., Wang, V., Qi, Y., Wilkerson, M.D., Miller, C.R., Ding, L., Golub, T., Mesirov, J.P., et al. (2010). Integrated Genomic Analysis Identifies Clinically Relevant Subtypes of Glioblastoma Characterized by Abnormalities in PDGFRA, IDH1, EGFR, and NF1. Cancer Cell *17*, 98–110. https://doi.org/10.1016/j.ccr.2009.12.020.
6. Ge, H., Mu, L., Jin, L., Yang, C., Chang, Y.E., Long, Y., DeLeon, G., Deleyrolle, L., Mitchell, D.A., Kubilis, P.S., et al. (2017). Tumor associated CD70 expression is involved in promoting tumor migration and macrophage infiltration in GBM. Int. J. Cancer *141*, 1434–1444. https://doi.org/10.1002/ijc.30830.
7. Seyfrid, M., Maich, W.T., Shaikh, V.M., Tatari, N., Upreti, D., Piyasena, D., Subapanditha, M., Savage, N., McKenna, D., Mikolajewicz, N., et al. (2022). CD70 as an actionable immunotherapeutic target in recurrent glioblastoma and its microenvironment. J. Immunother. Cancer *10*, e003289. https://doi.org/10.1136/jitc-2021-003289.
8. Wischhusen, J., Jung, G., Radovanovic, I., Beier, C., Steinbach, J.P., Rimner, A., Huang, H., Schulz, J.B., Ohgaki, H., Aguzzi, A., et al. (2002). Identification of CD70-mediated apoptosis of immune effector cells as a novel immune escape pathway of human glioblastoma. Cancer Res. *62*, 2592–2599.
9. Yoon, Y., Ao, Z., Cheng, Y., Schlossman, S.F., and Prasad, K.V. (1999). Murine Siva-1 and Siva-2, alternate splice forms of the mouse Siva gene, both bind to CD27 but differentially transduce apoptosis. Oncogene *18*, 7174–7179. https://doi.org/10.1038/sj.onc.1203144.
10. Prasad, K.V., Ao, Z., Yoon, Y., Wu, M.X., Rizk, M., Jacquot, S., and Schlossman, S.F. (1997). CD27, a member of the tumor necrosis factor receptor family, induces apoptosis and binds to Siva, a proapoptotic protein. Proc. Natl. Acad. Sci. USA *94*, 6346–6351. https://doi.org/10.1073/pnas.94.12.6346.
11. Wensveen, F.M., Unger, P.-P.A., Kragten, N.A.M., Derks, I.A.M., Ten Brinke, A., Arens, R., Van Lier, R.A.W., Eldering, E., and Van Gisbergen, K.P.J.M. (2012). CD70-Driven Costimulation Induces Survival or Fas-Mediated Apoptosis of T Cells Depending on Antigenic Load. J. Immunol. *188*, 4256–4267. https://doi.org/10.4049/jimmunol.1102889.
12. Jin, L., Tao, H., Karachi, A., Long, Y., Hou, A.Y., Na, M., Dyson, K.A., Grippin, A.J., Deleyrolle, L.P., Zhang, W., et al. (2019). CXCR1- or CXCR2-modified CAR T cells co-opt IL-8 for maximal antitumor efficacy in solid tumors. Nat. Commun. *10*, 4016. https://doi.org/10.1038/s41467-019-11869-4.
13. Zhao, Z., Condomines, M., van der Stegen, S.J.C., Perna, F., Kloss, C.C., Gunset, G., Plotkin, J., and Sadelain, M. (2015). Structural Design of Engineered Costimulation Determines Tumor Rejection Kinetics and Persistence of CAR T Cells. Cancer Cell *28*, 415–428. https://doi.org/10.1016/j.ccell.2015.09.004.
14. Philipson, B.I., O'Connor, R.S., May, M.J., June, C.H., Albelda, S.M., and Milone, M.C. (2020). 4-1BB costimulation promotes CAR T cell survival through noncanonical NF-κB signaling. Sci. Signal. *13*, eaay8248. https://doi.org/10.1126/scisignal.aay8248.
15. Kawalekar, O.U., O' Connor, R.S., Fraietta, J.A., Guo, L., McGettigan, S.E., Posey, A.D., Patel, P.R., Guedan, S., Scholler, J., Keith, B., et al. (2016). Distinct Signaling of Coreceptors Regulates Specific Metabolism Pathways and Impacts Memory Development in CAR T Cells. Immunity *44*, 712. https://doi.org/10.1016/j.immuni.2016.02.023.
16. Chih, Y.-C., Dietsch, A.C., Koopmann, P., Ma, X., Agardy, D.A., Zhao, B., De Roia, A., Kourtesakis, A., Kilian, M., Krämer, C., et al. (2025). Vaccine-induced T cell receptor T cell therapy targeting a glioblastoma stemness antigen. Nat. Commun. *16*, 1262. https://doi.org/10.1038/s41467-025-56547-w.
17. Nduom, E.K., Weller, M., and Heimberger, A.B. (2015). Immunosuppressive mechanisms in glioblastoma: Fig. 1. Neuro Oncol. *17*, vii9–vii14. https://doi.org/10.1093/neuonc/nov151.
18. Etzerodt, A., and Moestrup, S.K. (2013). CD163 and Inflammation: Biological, Diagnostic, and Therapeutic Aspects. Antioxid. Redox Signal. *18*, 2352–2363. https://doi.org/10.1089/ars.2012.4834.
19. Etzerodt, A., Tsalkitzi, K., Maniecki, M., Damsky, W., Delfini, M., Baudoin, E., Moulin, M., Bosenberg, M., Graversen, J.H., Auphan-Anezin, N., et al. (2019). Specific targeting of CD163+ TAMs mobilizes inflammatory monocytes and promotes T cell–mediated tumor regression. J. Exp. Med. *216*, 2394–2411. https://doi.org/10.1084/jem.20182124.
20. Grewal, I.S. (2008). CD70 as a therapeutic target in human malignancies. Expert Opin. Ther. Targets *12*, 341–351. https://doi.org/10.1517/14728222.12.3.341.
21. Tesselaar, K., Xiao, Y., Arens, R., Van Schijndel, G.M.W., Schuurhuis, D.H., Mebius, R.E., Borst, J., and Van Lier, R.A.W. (2003). Expression of the Murine CD27 Ligand CD70 In Vitro and In Vivo. J. Immunol. *170*, 33–40. https://doi.org/10.4049/jimmunol.170.1.33.
22. Arroyo Hornero, R., Georgiadis, C., Hua, P., Trzupek, D., He, L.-Z., Qasim, W., Todd, J.A., Ferreira, R.C., Wood, K.J., Issa, F., and Hester, J. (2020). CD70 expression

determines the therapeutic efficacy of expanded human regulatory T cells. Commun. Biol. *3*, 375. https://doi.org/10.1038/s42003-020-1097-8.

23. Abdelfattah, N., Kumar, P., Wang, C., Leu, J.-S., Flynn, W.F., Gao, R., Baskin, D.S., Pichumani, K., Ijare, O.B., Wood, S.L., et al. (2022). Single-cell analysis of human glioma and immune cells identifies S100A4 as an immunotherapy target. Nat. Commun. *13*, 767. https://doi.org/10.1038/s41467-022-28372-y.

24. Tarhan, L., Bistline, J., Chang, J., Galloway, B., Hanna, E., and Weitz, E. (2023). Single Cell Portal: an interactive home for single-cell genomics data. Preprint at bioRxiv. https://doi.org/10.1101/2023.07.13.548886.

25. Sánchez-Paulete, A.R., Mateus-Tique, J., Mollaoglu, G., Nielsen, S.R., Marks, A., Lakshmi, A., Khan, J.A., Wilk, C.M., Pia, L., Baccarini, A., et al. (2022). Targeting Macrophages with CAR T Cells Delays Solid Tumor Progression and Enhances Antitumor Immunity. Cancer Immunol. Res. *10*, 1354–1369. https://doi.org/10.1158/2326-6066.CIR-21-1075.

26. Rodriguez-Garcia, A., Lynn, R.C., Poussin, M., Eiva, M.A., Shaw, L.C., O'Connor, R.S., Minutolo, N.G., Casado-Medrano, V., Lopez, G., Matsuyama, T., and Powell, D.J., Jr. (2021). CAR-T cell-mediated depletion of immunosuppressive tumor-associated macrophages promotes endogenous antitumor immunity and augments adoptive immunotherapy. Nat. Commun. *12*, 877. https://doi.org/10.1038/s41467-021-20893-2.

27. Uhlén, M., Fagerberg, L., Hallström, B.M., Lindskog, C., Oksvold, P., Mardinoglu, A., Sivertsson, Å., Kampf, C., Sjöstedt, E., Asplund, A., et al. (2015). Tissue-based map of the human proteome. Science *347*, 1260419. https://doi.org/10.1126/science.1260419.

28. Wick, W., Dettmer, S., Berberich, A., Kessler, T., Karapanagiotou-Schenkel, I., Wick, A., Winkler, F., Pfaff, E., Brors, B., Debus, J., et al. (2019). N2M2 (NOA-20) phase I/II trial of molecularly matched targeted therapies plus radiotherapy in patients with newly diagnosed non-MGMT hypermethylated glioblastoma. Neuro Oncol. *21*, 95–105. https://doi.org/10.1093/neuonc/noy161.

29. Sauer, T., Parikh, K., Sharma, S., Omer, B., Sedloev, D., Chen, Q., Angenendt, L., Schliemann, C., Schmitt, M., Müller-Tidow, C., et al. (2021). CD70-specific CAR T cells have potent activity against acute myeloid leukemia without HSC toxicity. Blood *138*, 318–330. https://doi.org/10.1182/blood.2020008221.

30. Day, B.W., Stringer, B.W., Wilson, J., Jeffree, R.L., Jamieson, P.R., Ensbey, K.S., Bruce, Z.C., Inglis, P., Allan, S., Winter, C., et al. (2013). Glioma Surgical Aspirate: A Viable Source of Tumor Tissue for Experimental Research. Cancers *5*, 357–371. https://doi.org/10.3390/cancers5020357.

31. D'Souza, R.C.J., Offenhäuser, C., Straube, J., Baumgartner, U., Kordowski, A., Li, Y., Stringer, B.W., Alexander, H., Lwin, Z., Inglis, P.-L., et al. (2020). Q-Cell Glioblastoma Resource: Proteomics Analysis Reveals Unique Cell-States Are Maintained in 3D Culture. Cells *9*, 267. https://doi.org/10.3390/cells9020267.

32. Stringer, B.W., Day, B.W., D'Souza, R.C.J., Jamieson, P.R., Ensbey, K.S., Bruce, Z.C., Lim, Y.C., Goasdoué, K., Offenhäuser, C., Akgül, S., et al. (2019). A reference collection of patient-derived cell line and xenograft models of proneural, classical and mesenchymal glioblastoma. Sci. Rep. *9*, 4902. https://doi.org/10.1038/s41598-019-41277-z.

33. Wolfl, M., Kuball, J., Ho, W.Y., Nguyen, H., Manley, T.J., Bleakley, M., and Greenberg, P.D. (2007). Activation-induced expression of CD137 permits detection, isolation, and expansion of the full repertoire of CD8+ T cells responding to antigen without requiring knowledge of epitope specificities. Blood *110*, 201–210. https://doi.org/10.1182/blood-2006-11-056168.

34. Chambers, S.M., Fasano, C.A., Papapetrou, E.P., Tomishima, M., Sadelain, M., and Studer, L. (2009). Highly efficient neural conversion of human ES and iPS cells by dual inhibition of SMAD signaling. Nat. Biotechnol. *27*, 275–280. https://doi.org/10.1038/nbt.1529.

35. Hausmann, D., Hoffmann, D.C., Venkataramani, V., Jung, E., Horschitz, S., Tetzlaff, S.K., Jabali, A., Hai, L., Kessler, T., Azoŕin, D.D., et al. (2023). Autonomous rhythmic activity in glioma networks drives brain tumour growth. Nature *613*, 179–186. https://doi.org/10.1038/s41586-022-05520-4.

36. Feucht, J., and Sadelain, M. (2020). Function and evolution of the prototypic CD28ζ and 4-1BBζ chimeric antigen receptors. Immunooncol. Technol. *8*, 2–11. https://doi.org/10.1016/j.iotech.2020.09.001.

37. Gattinoni, L., Speiser, D.E., Lichterfeld, M., and Bonini, C. (2017). T memory stem cells in health and disease. Nat. Med. *23*, 18–27. https://doi.org/10.1038/nm.4241.

38. Vigano, S., Negron, J., Ouyang, Z., Rosenberg, E.S., Walker, B.D., Lichterfeld, M., and Yu, X.G. (2015). Prolonged Antiretroviral Therapy Preserves HIV-1-Specific CD8 T Cells with Stem Cell-Like Properties. J. Virol. *89*, 7829–7840. https://doi.org/10.1128/JVI.00789-15.

39. Oliveira, G., Ruggiero, E., Stanghellini, M.T.L., Cieri, N., D'Agostino, M., Fronza, R., Lulay, C., Dionisio, F., Mastaglio, S., Greco, R., et al. (2015). Tracking genetically engineered lymphocytes long-term reveals the dynamics of T cell immunological memory. Sci. Transl. Med. *7*, 317ra198. https://doi.org/10.1126/scitranslmed.aac8265.

40. Hambardzumyan, D., Gutmann, D.H., and Kettenmann, H. (2016). The role of microglia and macrophages in glioma maintenance and progression. Nat. Neurosci. *19*, 20–27. https://doi.org/10.1038/nn.4185.

41. Chang, A.L., Miska, J., Wainwright, D.A., Dey, M., Rivetta, C.V., Yu, D., Kanojia, D., Pituch, K.C., Qiao, J., Pytel, P., et al. (2016). CCL2 Produced by the Glioma Microenvironment Is Essential for the Recruitment of Regulatory T Cells and Myeloid-Derived Suppressor Cells. Cancer Res. *76*, 5671–5682. https://doi.org/10.1158/0008-5472.CAN-16-0144.

42. Jacob, F., Salinas, R.D., Zhang, D.Y., Nguyen, P.T.T., Schnoll, J.G., Wong, S.Z.H., Thokala, R., Sheikh, S., Saxena, D., Prokop, S., et al. (2020). A Patient-Derived Glioblastoma Organoid Model and Biobank Recapitulates Inter- and Intra-tumoral Heterogeneity. Cell *180*, 188–204.e22. https://doi.org/10.1016/j.cell.2019.11.036.

43. Jacob, F., Ming, G.L., and Song, H. (2020). Generation and biobanking of patient-derived glioblastoma organoids and their application in CAR T cell testing. Nat. Protoc. *15*, 4000–4033. https://doi.org/10.1038/s41596-020-0402-9.

44. Vigano, S., Bobisse, S., Coukos, G., Perreau, M., and Harari, A. (2020). Cancer and HIV-1 Infection: Patterns of Chronic Antigen Exposure. Front. Immunol. *11*, 1350. https://doi.org/10.3389/fimmu.2020.01350.

45. Vitanza, N.A., Johnson, A.J., Wilson, A.L., Brown, C., Yokoyama, J.K., Künkele, A., Chang, C.A., Rawlings-Rhea, S., Huang, W., Seidel, K., et al. (2021). Locoregional infusion of HER2-specific CAR T cells in children and young adults with recurrent or refractory CNS tumors: an interim analysis. Nat. Med. *27*, 1544–1552. https://doi.org/10.1038/s41591-021-01404-8.

46. Boulch, M., Cazaux, M., Loe-Mie, Y., Thibaut, R., Corre, B., Lemaître, F., Grandjean, C.L., Garcia, Z., and Bousso, P. (2021). A cross-talk between CAR T cell subsets and the tumor microenvironment is essential for sustained cytotoxic activity. Sci. Immunol. *6*, eabd4344. https://doi.org/10.1126/sciimmunol.abd4344.

47. Alizadeh, D., Wong, R.A., Gholamin, S., Maker, M., Aftabizadeh, M., Yang, X., Pecoraro, J.R., Jeppson, J.D., Wang, D., Aguilar, B., et al. (2021). IFNγ Is Critical for CAR T Cell–Mediated Myeloid Activation and Induction of Endogenous Immunity. Cancer Discov. *11*, 2248–2265. https://doi.org/10.1158/2159-8290.CD-20-1661.

48. Pituch, K.C., Miska, J., Krenciute, G., Panek, W.K., Li, G., Rodriguez-Cruz, T., Wu, M., Han, Y., Lesniak, M.S., Gottschalk, S., and Balyasnikova, I.V. (2018). Adoptive Transfer of IL13Rα2-Specific Chimeric Antigen Receptor T Cells Creates a Pro-inflammatory Environment in Glioblastoma. Mol. Ther. *26*, 986–995. https://doi.org/10.1016/j.ymthe.2018.02.001.

49. Kourtesakis, A., Bailey, E., Chow, H.N.H., Rohdjeß, H., Mussnig, N., Agardy, D.A., Hoffmann, D.C.F., Chih, Y.-C., Will, R., Kaulen, L., et al. (2025). Utilization of universal-targeting mSA2 CAR-T cells for the treatment of glioblastoma. OncoImmunology *14*, 2518631. https://doi.org/10.1080/2162402x.2025.2518631.

50. Hai, L., Hoffmann, D.C., Wagener, R.J., Azorin, D.D., Hausmann, D., Xie, R., Huppertz, M.-C., Hiblot, J., Sievers, P., Heuer, S., et al. (2024). A clinically applicable connectivity signature for glioblastoma includes the tumor network driver CHI3L1. Nat. Commun. *15*, 968. https://doi.org/10.1038/s41467-024-45067-8.

OMTON, Volume *34*

Supplemental information

A comparative analysis of CD70-directed CAR-T cells for glioblastoma treatment demonstrates a superior efficacy of the ligand-based construct

Alexandros Kourtesakis, Hiu Nam Hannah Chow, Eileen Bailey, Sandra Horschitz, Ammar Jabali, Rainer Will, Christoph Schifflers, Abigail K. Suwala, Hannah Rohdjess, Melissa Hahn, Yu-Chan Chih, Ling Hai, Denise Reibold, Sonja Pusch, Manuel Fischer, Ralph Sinn, Dennis Alexander Agardy, Dirk Carsten Frieder Hoffmann, Michael O. Breckwoldt, Robin Wagener, Leon Kaulen, Philipp Koch, Andreas von Deimling, Lukas Bunse, Michael Platten, Felix Sahm, Carsten Müller-Tidow, Michael Schmitt, Wolfgang Wick, Tim Sauer, and Tobias Kessler

Supplemental Figures

A)

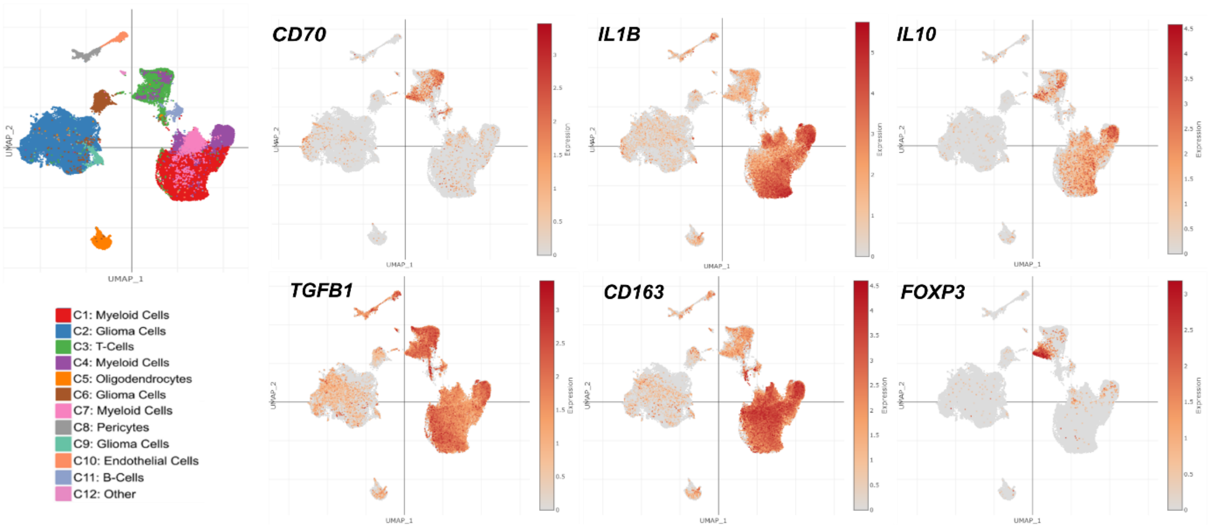

B)

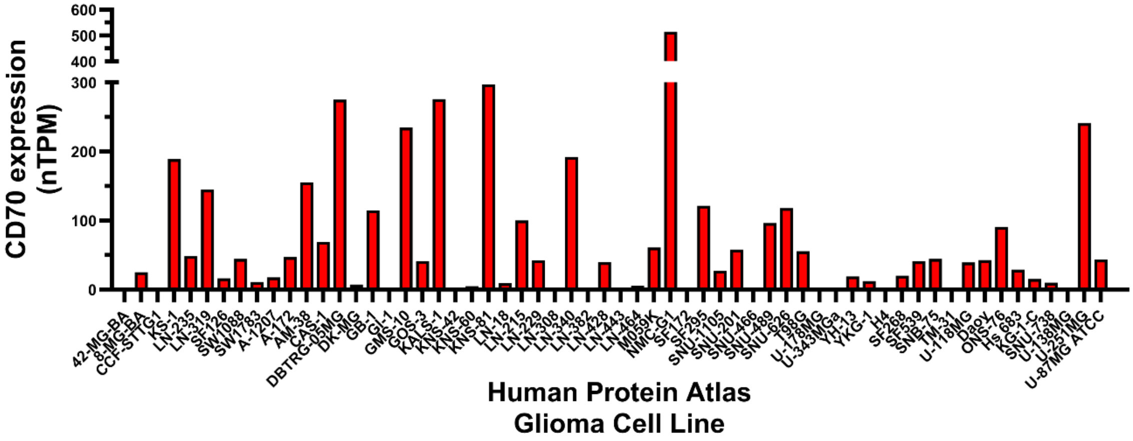

C)

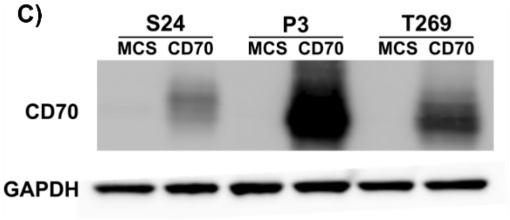

Figure S1: CD70 validation as a therapeutic target in GB. A) Expression of *CD70* and selected markers from a published single cell RNAseq dataset. **B)** *CD70* gene expression levels in selected glioma cell lines from Human Protein Atlas (version 24.0), determined by RNAseq. **C)** Investigation of CD70 expression in generated GB OE models by Western blot.

A)

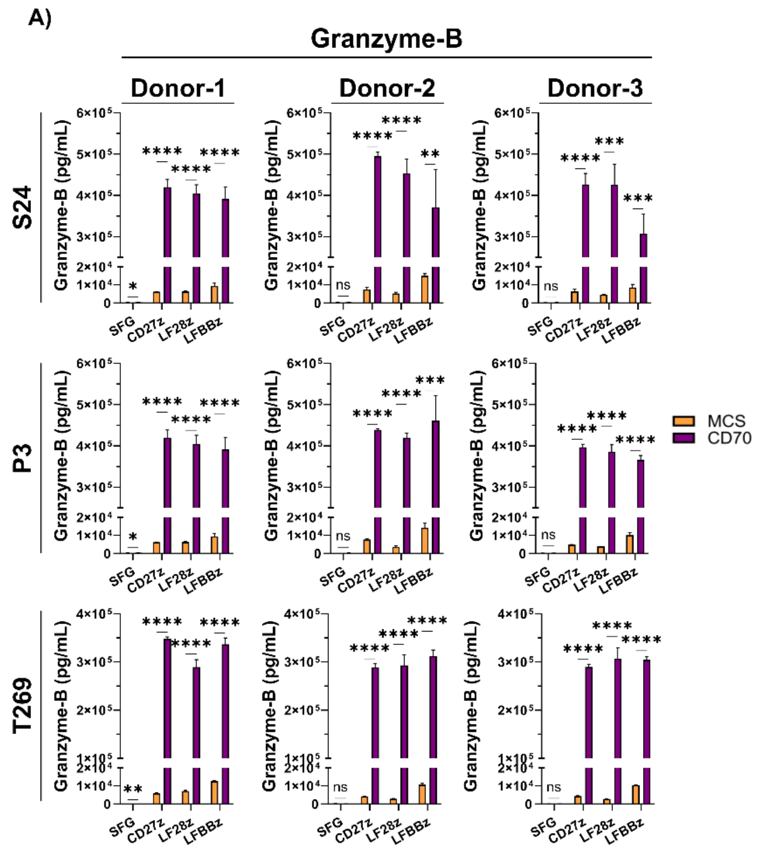

	S24/CD70			P3/CD70			T269/CD70		
	Donor-1	Donor-2	Donor-3	Donor-1	Donor-2	Donor-3	Donor-1	Donor-2	Donor-3
CD27z vs LF28z	ns	ns	ns	ns	ns	ns	***	ns	ns
CD27z vs LFBBz	*	*	*	ns	ns	*	ns	ns	ns
LF28z vs LFBBz	*	ns	*	ns	ns	ns	**	ns	ns

B)

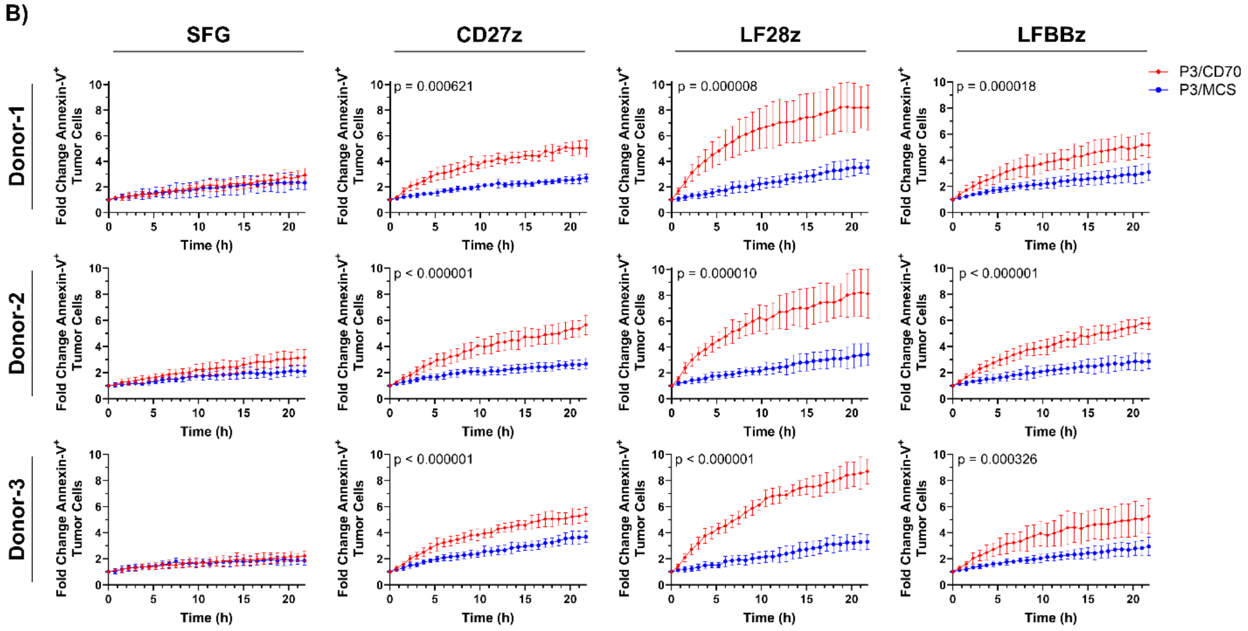

<u>Figure S2</u>: Assessment of Granzyme-B and apoptosis in co-culture between GB cells and CD70-directed CAR-T cells. A) Measurement of secreted Granzyme-B in the SN of GB/CAR-T cell co-cultures by ELISA. N = 3 biological replicates per group. For comparison between MCS and CD70 (upper barplots), an unpaired two-tailed t-test was used. For comparisons among constructs (bottom panel), a one-way ANOVA followed by a post-hoc Holm-Šídák multiple comparisons test was used. **B)** Quantification of Annexin-V binding by tumor cells during co-culture with CD70-directed CAR-T cells on the Incucyte platform. N = 2 biological replicates per group. Every biological replicate is the mean of N = 5 technical replicates. Timepoint intervals = 45 min. A two-tailed student's t-test was performed using the values of the last measured timepoint to determine statistical significance. For A) and B), data presented as mean (SD). *p <0.05, **p<0.01, ***p<0.001, ****p<0.0001; n.s., not significant.

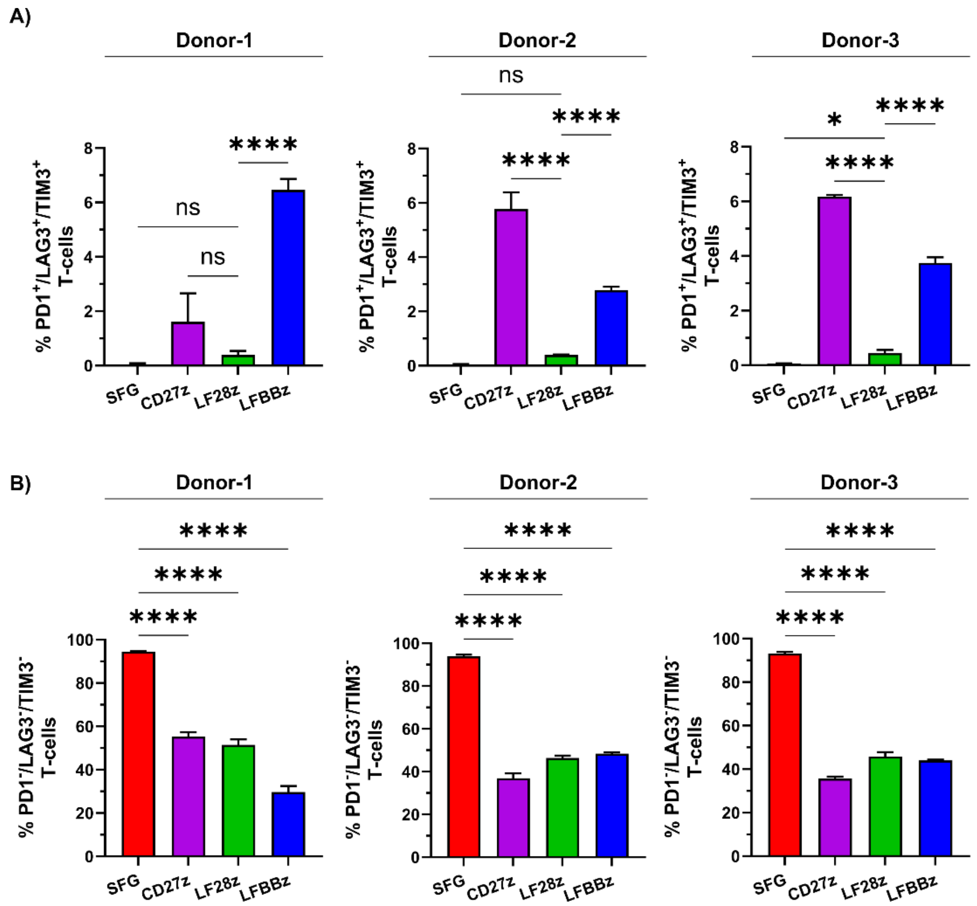

Figure S3: Assessment of exhaustion markers on the CAR-T cell surface after engagement with CD70-positive GB cells. A) Quantification of PD1/LAG3/TIM3 triple-positive effector cells after co-culture with P3/CD70 cells, determined by flow cytometry. **B)** Quantification of PD1/LAG3/TIM3 triple-negative effector cells after co-culture with P3/CD70 cells, determined by flow cytometry. For A) and B), isotype controls were used for gating. N=3 biological replicates per group. Data gated on single live $CD3^{+}$/$tdTomato^{+}$ cells. A one-way ANOVA followed by a Dunnett's multiple comparisons test was performed to assess significance. Data presented as mean (SD). *p <0.05, **p<0.01, ***p<0.001, ****p<0.0001; n.s., not significant.

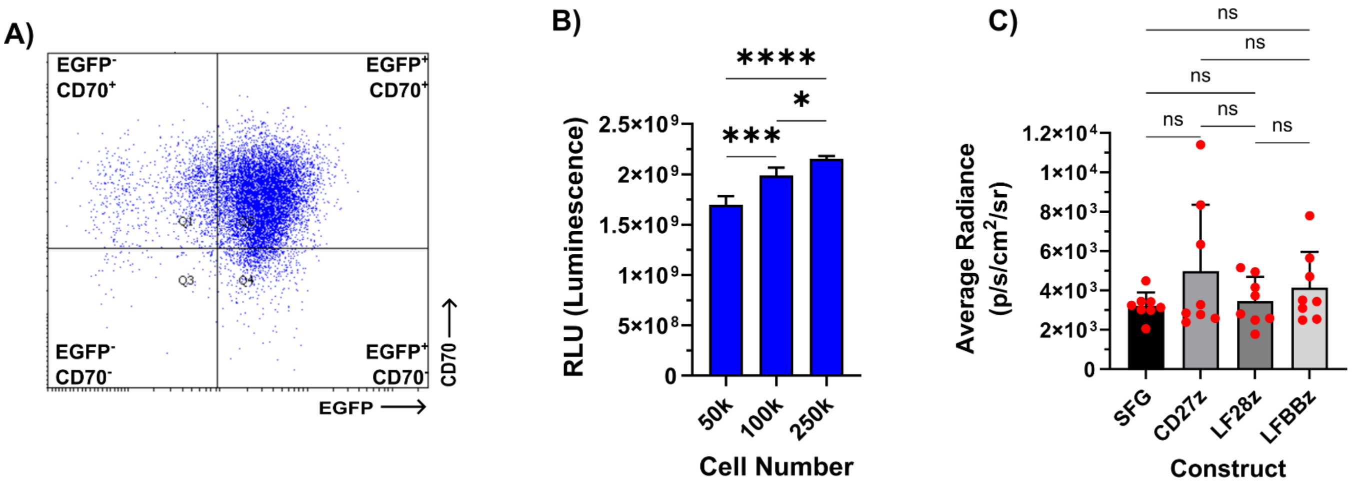

<u>Figure S4</u>: Verification of the NLuc/EGFP gene functionality before orthotopic implantation and animal stratification into treatment groups. A) Evaluation of the EGFP and CD70 signal of P3/CD70 GB cells transduced with the pCDH_NLuc/EGFP lentiviral construct by flow cytometry. An isotype and unstained control was used for gating. Data gated on single live cells. **B)** *In vitro* luminescence assay using GB cells from A). N = 3 biological replicates per group. A one-way ANOVA with a post-hoc Tukey's multiple comparisons test was performed for statistical significance. **C)** Comparison of tumor cell signal in NSG mice orthotopically implanted with GB cells one day before CAR-T cell treatment, stratified in treatment groups, determined by BLI. Each dot represents an animal. N = 8 animals per group. A Welch's ANOVA with a post-hoc Dunnett's T3 multiple comparisons test was perform to assess statistical significance. For B) and C), data presented as mean (SD). *p <0.05, **p<0.01, ***p<0.001, ****p<0.0001; n.s., not significant.

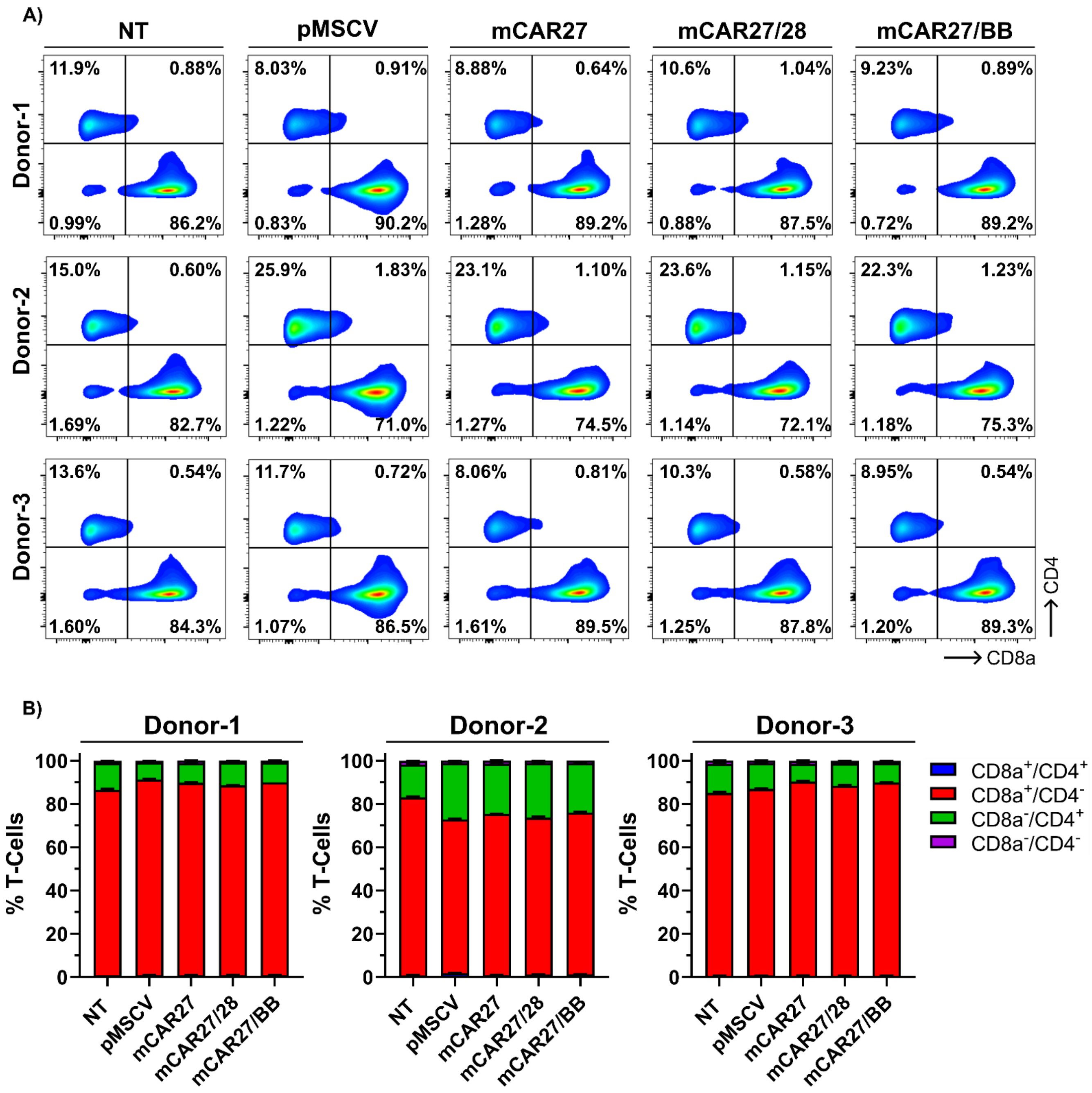

	Donor-1					Donor-2					Donor-3				
	NT	pMSCV	mCAR27	mCAR27/28	mCAR27/BB	NT	pMSCV	mCAR27	mCAR27/28	mCAR27/BB	NT	pMSCV	mCAR27	mCAR27/28	mCAR27/BB
$CD8a^+/CD4^-$ vs $CD8a^+/CD4^+$	****	****	****	****	****	****	****	****	****	****	****	****	****	****	****
$CD8a^+/CD4^-$ vs $CD8a^-/CD4^+$	****	****	****	****	****	****	****	****	****	****	****	****	****	****	****
$CD8a^+/CD4^-$ vs $CD8a^-/CD4^-$	****	****	****	****	****	****	****	****	****	****	****	****	****	****	****

<u>Figure S5</u>: Phenotyping of freshly produced mCD27-based mCD70-targeting CAR-T cells. A) Expression of mCD8a and mCD4 on transduced murine T-cells, determined by flow cytometry. Indicative results from one out of N=3 biological replicates per mouse donor per group. Isotype controls were used for gating. Data gated on single live $CD3^+$ cells (NT) or single live $CD3^+$/$tdTomato^+$ cells (pMSCV, mCAR27, mCAR27/28, mCAR27/BB). **B)** Quantification and comparison of different fractions from A). A one-way ANOVA followed by a Dunnett's multiple comparisons test was performed to assess significance. Data presented as mean (SD). *p <0.05, **p<0.01, ***p<0.001, ****p<0.0001; n.s., not significant.

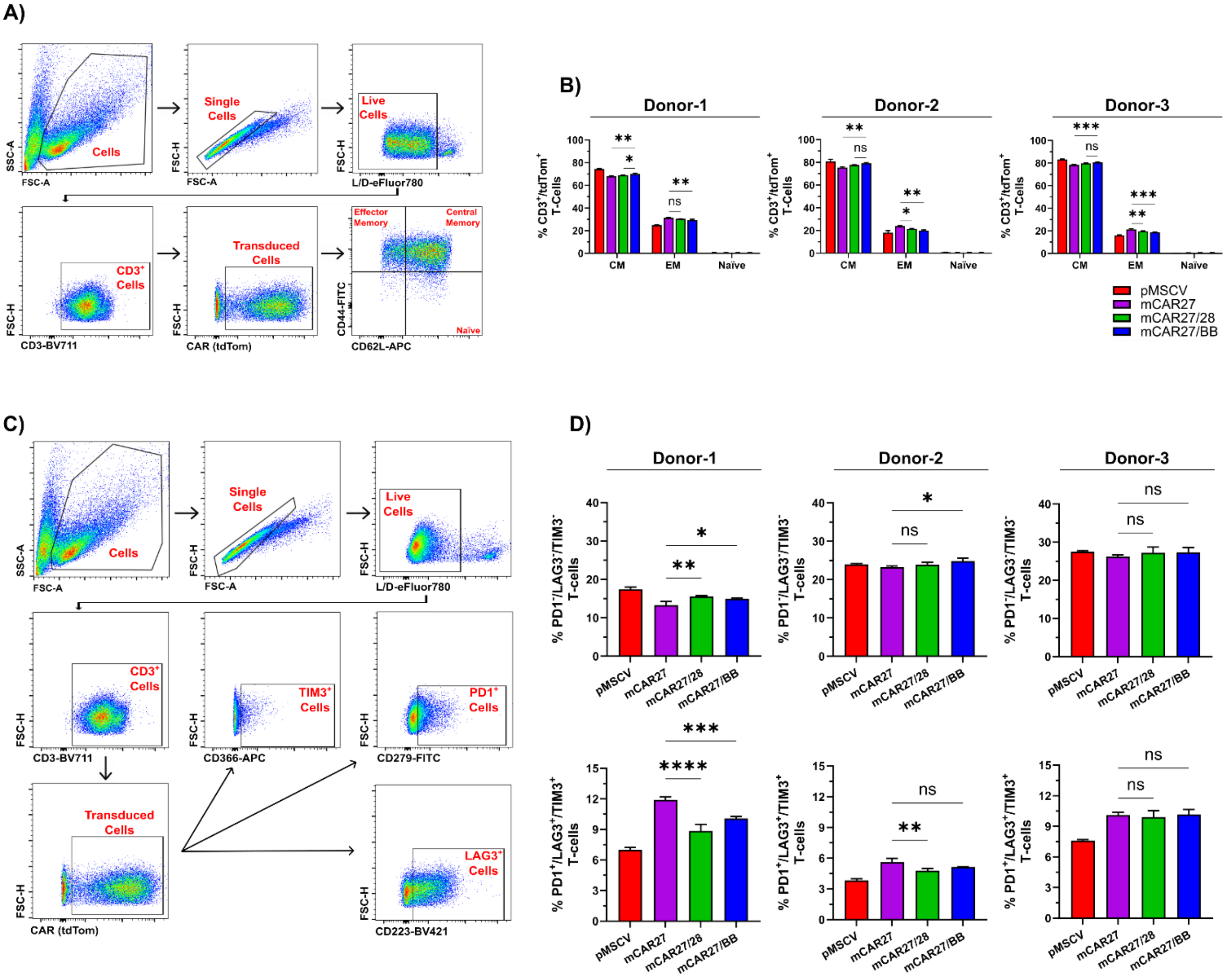

Figure S6: Assessment of memory markers and inhibitory receptors on mCAR-T cells. A) Gating strategy for immune cell memory marker quantification. **B)** Assessment of immune cell memory markers on the surface of mCAR-T cells by flow cytometry. **C)** Gating strategy for exhaustion marker quantification. **D)** Assessment of exhaustion markers on the surface of mCAR-T cells by flow cytometry. For B) and D), N=3 biological replicates per group. Data gated on single live CD3^{+}/tdTomato^{+} cells. Isotype control antibodies were used for gating. A one-way ANOVA with a post-hoc Holm-Šídák test was used for significance. Data presented as mean (SD). *p <0.05, **p<0.01, ***p<0.001, ****p<0.0001; n.s., not significant.

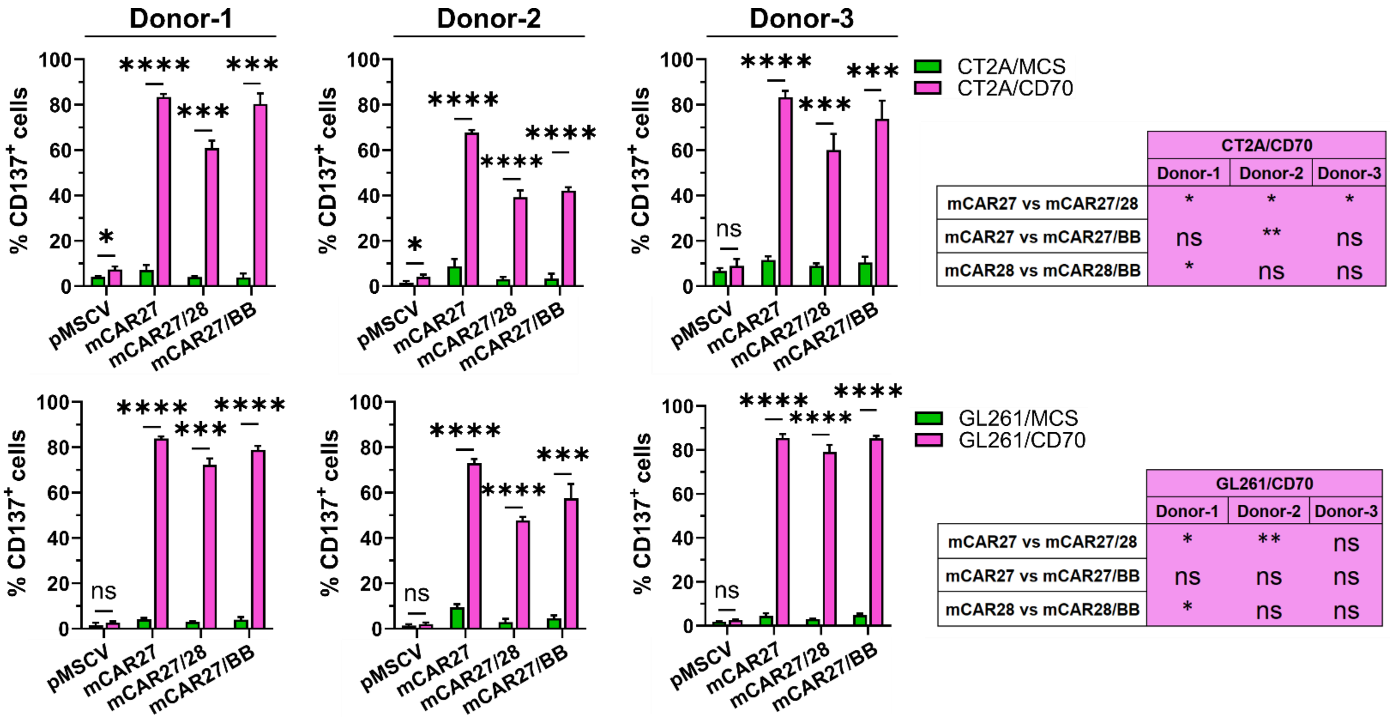

	CT2A/CD70		
	Donor-1	Donor-2	Donor-3
mCAR27 vs mCAR27/28	*	*	*
mCAR27 vs mCAR27/BB	ns	**	ns
mCAR28 vs mCAR28/BB	*	ns	ns

	GL261/CD70		
	Donor-1	Donor-2	Donor-3
mCAR27 vs mCAR27/28	*	**	ns
mCAR27 vs mCAR27/BB	ns	ns	ns
mCAR28 vs mCAR28/BB	*	ns	ns

<u>Figure S7</u>: Assessment of mCD70-targeting CAR-T cell activation upon co-culture with target murine GB cells. A) Measurement of activation marker mCD137 on the surface of murine CAR-T cells after O/N co-culture with the generated murine GB models by flow cytometry. N=3 biological replicates per group. Data gated on single live $CD3^+$ cells (NT) or single live $CD3^+$/$tdTomato^+$ cells (pMSCV, mCAR27, mCAR27/28, mCAR27/BB). Isotype control antibodies were used. An unpaired two-tailed t-test was used for comparisons. A one-way ANOVA with a post-hoc Holm-Šídák test was used for significance between selected construct pairs (upper right and bottom right panels). Data presented as mean (SD). *p <0.05, **p<0.01, ***p<0.001, ****p<0.0001; n.s., not significant.

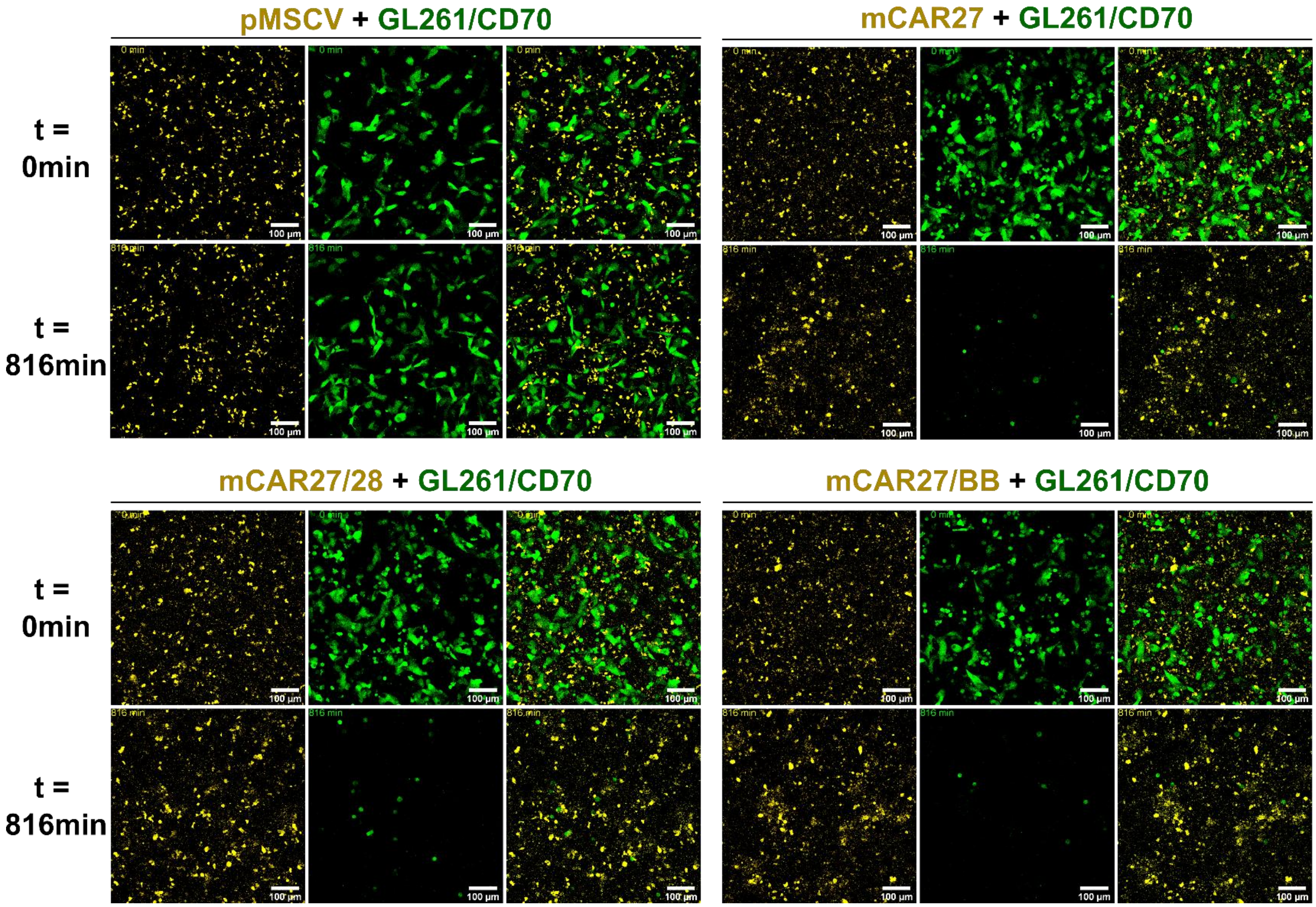

Figure S8: ***In vitro* potency of anti-mCD70 ligand-based CAR-T cells.** Confocal IF images of GL261/CD70_EGFP cells at the start (t=0 min) and the end (t=816 min) of co-culture with mCD27-based mCD70-targeting CAR-T cells or mock-transduced murine T-cells. Scale bar = 100 µm.

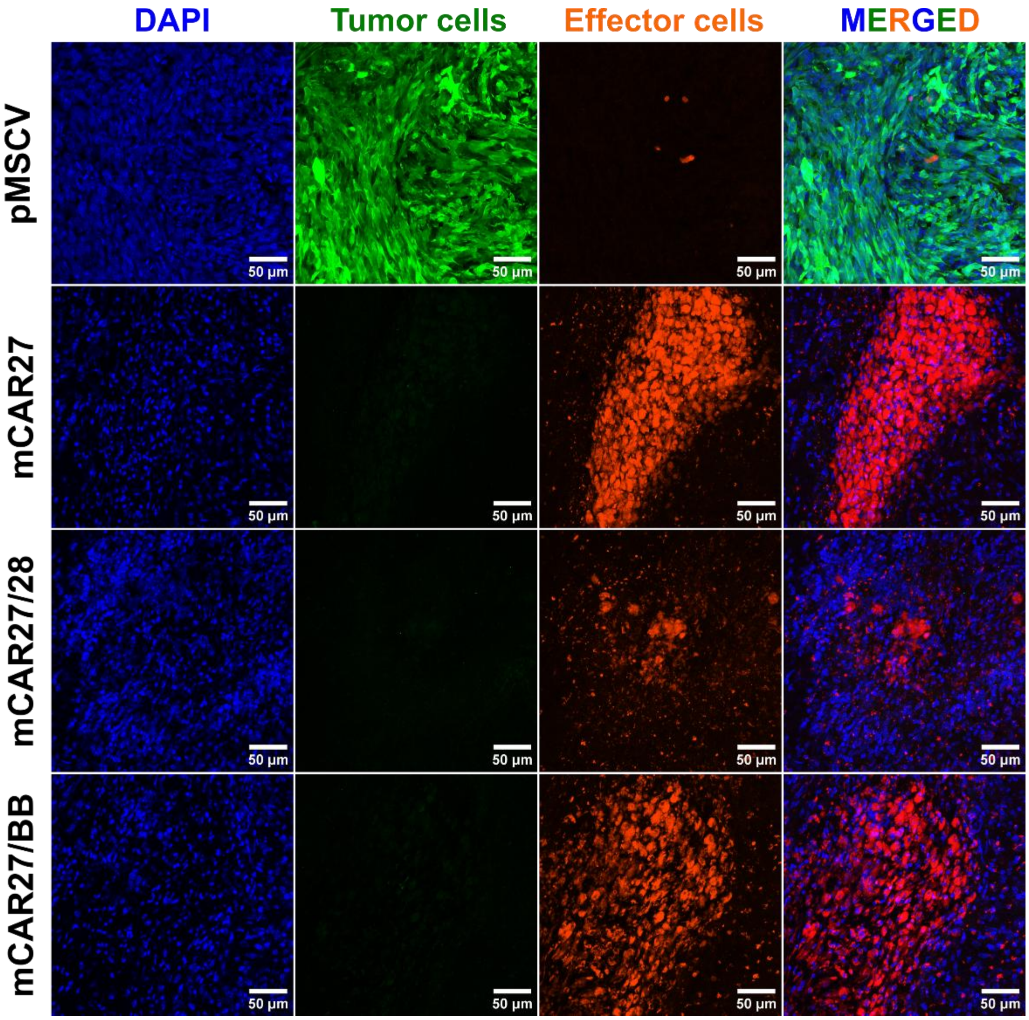

<u>Figure S9</u>: Detection of mCAR-T cells in the brains of treated C57BL/6J mice. Analysis of mCAR-T cell persistence in the brains of C57BL/6J mice treated with mCD70-targeting CAR-T cells from Figure 8A by IF. Representative images from N = 2 mice per treatment arm. Scale bar = 50 µm.

Supplemental Tables

Table S1: Glioblastoma patient characteristics

Sample ID	Age at diagnosis	Gender	Entity	MGMT promoter status	Prior therapy
D170-107	63	male	glioblastoma	methylated	none
D170-108	83	female	glioblastoma	methylated	none
D170-109	83	male	glioblastoma	non methylated	none
D170-112	78	female	glioblastoma	non methylated	none
D170-117	62	female	glioblastoma	non methylated	none
D170-21	63	male	glioblastoma	non methylated	none
D170-27	69	male	glioblastoma	methylated	none
D170-44	64	male	glioblastoma	methylated	none
D170-55	75	female	glioblastoma	non methylated	none
D170-56	80	male	glioblastoma	non methylated	none
D170-63	67	male	glioblastoma	non methylated	none
D170-69	79	female	glioblastoma	methylated	none
D170-83	78	male	glioblastoma	non methylated	none
D170-84	55	female	glioblastoma	non methylated	none
D170-93	42	male	glioblastoma	non methylated	none
D170-99	56	male	glioblastoma	non methylated	none

Supplemental Methods

Western Blot

Total protein from $2*10^6$ GB cells was extracted and quantified with the Pierce™ Bradford Protein Assay Kit (#23200, Thermo Fisher Scientific) according to the manufacturer's recommendations. Samples were prepared in NuPAGE™ LDS Sample Buffer (#NP0007, Invitrogen) and reduced in NuPAGE™ Sample Reducing Agent (#NP0009, Invitrogen). 20 µg of total protein extracted from GB cells was loaded on a 4-12 % (w/v) NuPAGE™ Bis-Tris Mini Protein Gel (#NP0321BOX, Invitrogen) and run at 200 V on the 1 h on the XCell SureLock Mini-Cell system (#EI0001, Invitrogen) in NuPAGE™ MOPS SDS Running Buffer (#NP0001, Invitrogen), in the presence of NuPAGE™ Antioxidant (#NP0005, Invitrogen). The transfer 0.45 µm Immobilon®-P Membrane (#IPVH85R, Merck Millipore) was activated with methanol (#M/4000/PC17, Fisher Chemical) and transfer was performed for 1 h at 200 mA in NuPAGE™ Transfer Buffer (#NP00061, Invitrogen). The membrane was then blocked in 5% (w/v) powdered milk (#T145.2, Carl Roth) for 1 h at RT and incubated with a rabbit anti-human CD70 antibody (1:1000; #69209S, Cell Signaling Technology) and mouse anti-human GAPDH (1:1000; #97166, Cell Signaling Technology) as loading control overnight (O/N) at 4 °C. Next, it was washed three times in TBS-T at RT for a total duration of 1 h. The membrane was incubated with an HRP-linked goat anti-rabbit secondary antibody (#NA9340V, GE Healthcare) diluted 1:2000 in blocking buffer supplemented with 0.2 % (v/v) Tween-20 at RT for 1 h. After a final wash step (performed as above), membrane was incubated with the Clarity Western ECL Substrate (#170-5060, Bio-Rad) following the manufacturer's recommendations. Signal was detected using the Chemidoc imaging station (#17001402, Bio-Rad). The PageRuler™ Plus Prestained Protein Ladder (#26619, Thermo Fisher Scientific) was used as a molecular weight control.

Live-cell confocal microscopy

The 488 nm laser was used to detect EGFP-positive GL261/CD70_EGFP cells and the 555 nm laser to detect tdTomato^{+} effector cells (pMSCV, mCAR27, mCAR27/28, mCAR27/BB). Pixel resolution was 1024x1024 (694.7591x694.7591 microns, with one micron corresponding to 1.4739 pixels). A given pixel comprised 16 bits, the z-range was 15 microns with an interval of 3 microns between two given stacks. The plate was imaged inside a specially-configurated incubator chamber (37 °C, 5% CO_2). Acquired images were analyzed with Fiji (version 1.53c, National Institute of Health). Channels were first split and maximum intensity projections were generated. Afterwards, the brightness and contrast for every channel were adjusted evenly across conditions. For quantification of Gl261/CD70_EGFP signal, the respective channel was converted to binary by using the "*Triangle*" threshold method with dark background. The %-area of the field of view occupied by tumor cells (EGFP signal) was measured for all timepoints and normalized against the first measurement (t=0 min).

Flow Cytometry

For assessment of mCAR-T cell immune cell memory the following mix was used: anti-mouse CD3-BV711 (#100241, Biolegend, RRID: AB_2563945), anti-mouse CD62L-APC (#161217, Biolegend, RRID: AB_3662372), and anti-mouse CD44-FITC (#103005, Biolegend, RRID: AB_312956). For assessment of mCAR-T cell proliferative exhaustion the following mix was used: anti-mouse CD3-BV711 (#100241, Biolegend, RRID: AB_2563945), anti-mouse CD223-BV421 (#125221, Biolegend, RRID: AB_2572080), anti-mouse CD279-FITC (#135214, Biolegend, RRID: AB_10680238), and anti-mouse CD366-APC (#119706, Biolegend, RRID: AB_2561656). The following isotype antibodies were used: PE Mouse IgG3, k Isotype Ctrl Antibody (#556659, BD Biosciences, RRID: AB_396523), BV711 Mouse IgG2a, κ Isotype Ctrl Antibody (#400271,

Biolegend, RRID: AB_3097679), BV421 Mouse IgG1, κ Isotype Ctrl Antibody (#400157, Biolegend, RRID: AB_10897939), BV510 Mouse IgG1, κ Isotype Ctrl Antibody (#400171, Biolegend, RRID: AB_2714004), APC Mouse IgG2b, κ Isotype Ctrl Antibody (#401209, Biolegend, RRID: AB_2941901), PacificBlue Mouse IgG1, κ Isotype Ctrl Antibody (#400151, Biolegend, RRID: AB_2923473), APC Mouse IgG1, κ Isotype Ctrl Antibody (#400119, Biolegend, RRID: AB_2888687), FITC Mouse IgG2a, κ Isotype Ctrl Antibody (#400209, Biolegend, RRID: AB_326458), APC Mouse IgG2b, κ Isotype Ctrl Antibody (#400319, Biolegend, RRID: AB_326500), BV605 Mouse IgG2a, κ Isotype Ctrl Antibody (#400270, Biolegend, AB_3097669), APC Mouse IgG2a, κ Isotype Ctrl Antibody (#40029, Biolegend, RRID: AB_326468), BV421 Mouse IgG1, κ Isotype Ctrl Antibody (#400157, Biolegend, RRID: AB_10897939), PE Rat IgG2b, κ Isotype Ctrl Antibody (#400607, Biolegend, RRID: AB_326551), BV711 Rat IgG2b, κ Isotype Ctrl Antibody (#400653, Biolegend, RRID: AB_3097684), APC Rat IgG2a, κ Isotype Ctrl Antibody (#400512, Biolegend, RRID: AB_2814702), FITC Rat IgG2b, κ Isotype Ctrl Antibody (#400633, Biolegend, RRID: AB_893678), BV421 Rat IgG1, κ Isotype Ctrl Antibody (#400429, Biolegend, RRID: AB_10900998), FITC Rat IgG2a, κ Isotype Ctrl Antibody (#400505, Biolegend, RRID: AB_2736919).

Animal housing, welfare, and experimentation measures

Animals were kept in a pathogen-free facility, maintaining a temperature of 22 ± 2 °C, a humidity level of 55 ± 10%, and a 12-hour light/dark cycle. They were housed in individually ventilated cages (max = 5 animals per cage) and were provided an *ad libitum* standard diet according to the protocols of the German Cancer Research Center (DKFZ). To ensure ethical compliance, only the minimum number of animals necessary to achieve statistical significance was used and every effort was made to reduce animal distress, according to the 3R principles. Animals were monitored daily

throughout the course of all experiments and were terminated upon reaching pre-determined neurological signs, or a body weight loss of 20%, or at the end of the legally approved experimental period. All animal procedures took place at the preclinical research facility of DKFZ. To prevent corneal injury, protective eye cream was applied to the animals' eyes during the orthotopic implantation procedure, bioluminescence imaging, and MRI. To maintain physiological body temperature, all animals undergoing anesthesia were placed on a warming mat with a temperature set at 37°C. Post-surgery pain relief was provided via subcutaneous (s.c.) injection of carprofen (#15, Cp Pharma) at a dose of 10 mg/kg body weight, diluted in a 0.9% (w/v) NaCl solution (#630138, Braun) with a second dose given 24 h later. Carprofen was then administered to the animals via drinking water for 48 h following any surgical intervention. For assessment of human CD70-targeting constructs *in* vivo, a total of 32 NSG animals were used (N=8 animals per treatment group). For assessment of murine CD70-targeting constructs *in vitro* and *in vivo*, a total of 24 C57BL/6J animals were used (N=5 animals per treatment group *in vivo* + N=4 animals as murine T-cell donors for mCAR-T cell production for experimental use). In total, this study utilized N=56 animals. One animal served as one experimental unit. No experimental units or data points were excluded from the analysis. No tumor presence 4 weeks after orthotopic GB cell implantation, based on bioluminescence imaging (BLI), for NSG mice, or 14 days after orthotopic implantation, based on MRI, for C57BL/6J mice, served as an *a priori* exclusion criterion. All procedures on animals of different groups were carried out on the same day, with minimum delay between two given animals. To further reduce bias, we used the same instrumentation, reagents of the same batch, and applied the same data analysis pipelines and parameters for all animals.
